# *Parvimonas micra* is associated with tumour immune profiles in molecular subtypes of colorectal cancer

**DOI:** 10.1007/s00262-022-03179-4

**Published:** 2022-03-17

**Authors:** Thyra Löwenmark, Xingru Li, Anna Löfgren-Burström, Carl Zingmark, Agnes Ling, Therese G. Kellgren, Pär Larsson, Ingrid Ljuslinder, Sun Nyunt Wai, Sofia Edin, Richard Palmqvist

**Affiliations:** 1 Department of Medical Biosciences, Pathology, Umeå University, Building 6M, 90185 Umeå, Sweden; 2 Northern Register Centre, Department of Public Health and Clinical Medicine, Umeå University, Umeå, Sweden; 3 Department of Radiation Sciences, Oncology, Umeå University, Umeå, Sweden; 4 Department of Molecular Biology, Umeå University, Umeå, Sweden

**Keywords:** Mucosal microbiota, *P. micra*, *F. nucleatum*, Immunity, Colorectal cancer

## Abstract

The importance of the tumour microbiome in different aspects of colorectal cancer (CRC) has been increasingly recognised, but many questions remain. The aim of this study was to explore the effect of specific CRC associated microbes on the tumour immune response, which has a considerable prognostic value in CRC. We applied specific qPCR to detect *Parvimonas micra* and *Fusobacterium nucleatum* in tumour tissues from an immunologically well-characterised cohort of 69 CRC patients. This cohort included detailed analyses of immune profiles based on flow cytometry and transcriptomics in tumour tissue and blood, along with comprehensive analyses of molecular subtypes. *P. micra* and *F. nucleatum* were detected in 24% and 64% of tumour tissues, respectively. We found a significant association of *P. micra* with high-grade tumours and tumours of CMS1 subtype. *F. nucleatum* was significantly associated with right-sided tumours, microsatellite instability, and CMS1 tumours. The immunological analyses revealed significant associations of *P. micra* with activated CD69^+^ T lymphocytes and increased antigen-presenting HLA-DR^+^ B lymphocytes. *P. micra* was also positively associated with M1 and M2 macrophage traits. The impact of *P. micra* tumour colonisation on the immune response was further assessed using transcriptomics in validation of our findings. No associations were found between *F. nucleatum* and immune profiles in this study. Our findings support novel associations between *P. micra* and the immune response in CRC. A better understanding of these interactions might help to identify important predictive and prognostic tools as well as new targets for therapy.

**Supplementary Information:**

The online version contains supplementary material available at 10.1007/s00262-022-03179-4.

## Introduction

Colorectal cancer (CRC) is a heterogeneous disease that evolves through a complex interplay between environmental and genetic components. The role of the tumour microbiome in CRC progression has been increasingly recognised as a contributing factor to patient prognosis and response to therapy [1]. Still, relatively little is known about the functional mechanisms behind different aspects of clinical outcome, and thus, further studies elucidating the spatio-temporal presence of certain bacteria along with their interactions with each other and the evolving tumour are crucial. A driver-passenger theory has been proposed, where indigenous intestinal bacteria, often toxin producing, can induce epithelial DNA damage, inflammation, and the initiation of CRC (bacterial drivers). Along with tumorigenesis, intestinal niche alterations favour colonisation of certain opportunistic bacteria (bacterial passengers). The bacterial passengers outcompete the initial driver bacteria, and might further accelerate tumour progression through, for example, inflammatory processes [2].

Metagenomic studies have shown alterations (dysbiosis) of both the gut and mucosal microbiota associated with CRC [1, 3, 4]. Interestingly, several of the bacteria associated with CRC are commonly found in the oral flora, including the anaerobic bacteria *Fusobacterium nucleatum* and *Parvimonas micra,* and both these bacteria have been associated with various inflammatory conditions in, for example, the oral cavity and gut, as well as CRC [3–11]. In a previous study using specific qPCR assays, we confirmed in two independent cohorts a greater abundance of *F. nucleatum* and *P. micra* in the faeces of CRC patients compared to healthy controls [12]. The pathogenesis of *F. nucleatum* in CRC has been quite extensively studied and found to be associated with a worsened prognosis [13–15]. For instance, *F. nucleatum* has been shown to express a unique adhesion molecule, FadA, which enables it to adhere to and invade colonic epithelial cells, and to induce E-cadherin-mediated activation of Wnt/β-catenin signalling [16]. In contrast, very little is known about the mechanisms through which *P. micra* influences CRC progression.

In parallel with environmental factors such as the contribution of the gut microbiome to CRC, tumour formation has been associated with both genetic and epigenetic changes. The most robust classification today of CRC in terms of biological and clinical behaviour is the transcriptomics-based consensus molecular subtypes (CMSs) [17]. The CMS subgroups include CMS1 (hypermutation, microsatellite instability (MSI), *BRAF* mutation, CIMP high, immune activation), CMS2 (epithelial, significant WNT and MYC signalling), CMS3 (epithelial and evident metabolic dysregulation, *KRAS* mutation), and CMS4 (mesenchymal, prominent TGF-β activation, stromal invasion and angiogenesis).

The immune response is an important regulator of CRC progression and patients with tumours highly infiltrated by activated immune cells have a prognostic advantage [18]. Patients with MSI tumours (mainly found in CMS1) show increased local tumour immune cell infiltration and improved survival compared to patients with other tumour molecular subtypes [19]. The important prognostic role of the immune system in CRC, further suggests immunotherapy as a potential treatment modality. Cytotoxic T cells are negatively regulated by the immune checkpoint molecules, cytotoxic T lymphocyte-associated protein 4 (CTLA-4) and programmed cell death protein (PD-1/PDCD-1), and PD-1 and CTLA-4 inhibitors have been approved for CRC patients with metastatic MSI tumours and are associated with prolonged survival [20]. Notably, recent studies have proposed that the response to immunotherapy in CRC is partly affected by the gut microbiota [21].

In this study, we used specific qPCR assays to investigate the association between tumour colonisation of *P. micra* and *F. nucleatum* and immune events in an immunologically well-characterised cohort of CRC patients. A better understanding of the interactions between gut microbes and the immune response might lead to important improvements in future cancer care.

## Materials and methods

### Study cohort

Patients included in the study were from the Uppsala-Umeå Comprehensive Cancer Consortium (U-CAN) project [22]. Since 2010, the project has collected fresh frozen tissue, formalin-fixed paraffin-embedded tissue, blood, and clinical data from all patients diagnosed with CRC at the Umeå University Hospital, Umeå, Sweden. Between November 2015 and July 2017, U-CAN patients were included in the Umeå Immune Profiling of Colorectal Cancer Project (UIP-CRC) that is described in detail elsewhere [23]. In brief, a total of 69 patients were included in UIP-CRC. Of these, immune activity profiles were available from tumour tissue of 64 patients and blood from 49 patients. Non-fasting plasma samples, taken at the time of diagnosis, were also available from 63 patients. Patients with rectal cancer who had undergone irradiation therapy prior to surgery were excluded. The UIP-CRC cohort has been well-characterised regarding molecular determinants, including MSI status, *BRAF* and *KRAS* mutations status, and CMS subtype, as previously described [23].

### Analyses of immune activity profiles in isolated mononuclear immune cells using flow cytometry

The analysis of immune activity profiles from UIP-CRC has been previously described [23]. In brief, immune markers were analysed on isolated mononuclear immune cells from tumour tissues of 64 patients and blood samples from 49 patients using flow cytometry. Gating on the mononuclear cell population was done in the FSC/SSC window; thereafter, a gate was set to identify populations of T helper cells (CD3^+^/CD4^+^), cytotoxic T cells (CD3^+^/CD8^+^), monocytes/macrophages (CD14^+^), NK cells (CD56^+^/CD16^+^/CD3^−^), or B cells (CD19^+^). Second gates were set using FMO (fluorescence minus one) controls to evaluate the proportions of a population of gated cells expressing a specific marker (CD28, CD69, PD-1, CTLA-4, NKG2D, CD80, CD86, CD163, HLA-DR, or PD-L1). T regulatory cells (Tregs) were defined as CD4^+^CD25^+^CD127^−^.

### Detection of microbial markers in tissue using quantitative real‑time PCR (qPCR)

Microbial factors were analysed by qPCR in fresh frozen tumour tissues and adjacent non-malignant tissues from 67 of the patients. Two patients were excluded from qPCR analyses due to a lack of fresh frozen tissues. DNA was extracted from a 2–3 mm cube of fresh frozen tissue using the AllPrep DNA/RNA/miRNA Universal kit (Qiagen). Prior to extraction, the tumour tissue was homogenised using a Precellys 24 homogenizer (Bertin Technologies) with 1.4 mm ceramic beads. The qPCR assays for *P. micra*, *F. nucleatum* and the universal qPCR assay for the 16S rRNA gene, have previously been established and described with references to the relevant literature [12]. The *PGT* gene assay was acquired from Flanagan et al. [13] and Castellarin et al. [24]. Primers and probes of the different assays are listed in Supplementary Table 1.

All reactions were run in duplicates utilising the Quant-Studio™ 6 Flex Real-Time PCR System (Applied Biosystems). Cycle conditions for all assays used were: 2 min at 50 °C, 10 min at 95 °C, and 40 cycles of: 95 °C for 15 s, and 60 °C for 1 min. Markers not amplified within 38 cycles were defined as negative. In cases where there was a discrepancy in quantification cycle (*C*q) values between the duplicates (SD > 0.5), the sample was rerun in duplicates to obtain a stable duplicate. Samples with discrepancies in *C*q values between duplicates after three runs were excluded. These exclusions included one tumour tissue sample and two adjacent non-malignant tissue samples for *F. nucleatum* and one adjacent non-malignant tissue sample for the universal 16 s rRNA assay. In the analysis of *PGT*, one non-malignant tissue sample was excluded due to limited amounts of DNA. No exclusions were made for *P. micra.* The level of *P. micra* and *F. nucleatum* was presented as a relative quantification with the human gene *PGT* or the 16S rRNA gene as references and calculated using the 2^—ΔCq^ method.

### Transcriptomic-based analyses

The RNA sequencing analysis for the UIP-CRC cohort has been previously described [23]. DESeq2 was used for differential gene expression analysis [25]. The DESeq2 analysis was modelled in three different ways, (1) as a function of *P. micra* positive samples, (2) as a function of *F. nucleatum* positive samples, and (3) as a function of four groups; *P. micra* positives, *F. nucleatum* positives, samples positive for both *P. micra* and *F. nucleatum*, and samples negative for both *P. micra* and *F. nucleatum*. A *P*-adjusted value < 0.05 was used to filter significantly differentially expressed genes. GO enrichment analysis for biological processes [26, 27] was performed to functionally annotate differentially expressed genes using the R package clusterProfiler [28]. Principal component analyses (PCA) were used to illustrate clusters of samples based on similarities according to *P. micra* and *F.nucleatum* positivity. CIBERSORT_x_ was utilised to classify immune infiltration based on transcriptomics in *P. micra* positive and *P. micra* negative samples, enabling the analysis of immune cell type abundances in mixed tissues. The abundance of immune cells was computed using the leukocyte signature matrix LM22 [29]. The statistical programming language R, version 4.0.0 and 4.0.4 were used for the bioinformatics and statistical analyses [30]. For illustrations, the ggplot2 packages were used [31].

### Plasma analyses

Non-fasting EDTA plasma samples were collected at the time of diagnosis and stored at − 80 °C. Plasma samples were analysed using the Olink Immuno-Oncology panel (v3.111), Olink Biosciences, Uppsala, Sweden), which includes detection of 92 proteins by Proximity Extension Assay technology. The analysis was performed by SciLifeLab, Uppsala, Sweden, and targeted proteins were detected through qPCR [32]. The qPCR results were analysed as normalised protein expression (NPX) values on a log2-scale. A detailed description of assay characteristics, including quality control, detection limits, performance, and validation, can be found at https://www.olink.com.

### Statistical methods

Statistical analyses were performed using IBM SPSS Statistics 26 (SPSS Inc.). Fisher's exact test was used to compare categorical variables, and the Mann–Whitney *U* test was used to compare differences in continuous variables between two groups. For more than two groups, the Kruskall–Wallis test was used for comparisons of continuous variables. The Wilcoxon signed-rank test was used for pairwise dependent continuous variables, and correlations between continuous variables were analysed using the Spearman's rank correlation test. *P* values < 0.05 were considered statistically significant.

### Results

### Distribution of *P. micra* and *F. nucleatum* in tumour tissue and adjacent non-malignant tissue of CRC patients

The clinical characteristics of the 67 study patients are presented in Table 1. The levels of *P.micra* and *F. nucleatum* were determined by qPCR in fresh frozen tumour tissue and adjacent non-malignant tissue. To find the optimal reference gene for relative quantification, we initially used both a universal qPCR assay for the 16S rRNA gene and a qPCR assay for the human gene *PGT*. Spearman’s rank correlation tests revealed that the relative levels using either the 16S rRNA gene or *PGT* as reference were highly correlated for both *P. micra* and *F. nucleatum* (Spearman’s rank correlation coefficient (*r*_s_) = 0.992, *P* < 0.001 for *P. micra*, *r*_s_ = 0.923, *P* < 0.001 for *F. nucleatum*). Nonetheless, a significant positive correlation was found for non-normalised levels of *P. micra* and *F. nucleatum* with the 16S rRNA gene (*r*_s_ = 0.541, *P* = 0.030 and *r*_s_ = 0.453, *P* = 0.003, respectively), whereas no significant correlation was found for *P. micra* and *F. nucleatum* with human *PGT* (*r*_s_ = − 0.247, *P* = 0.356 and *r*_s_ = − 0.103 and *P* = 0.518, respectively). Therefore, for subsequent analyses, *P. micra* and *F. nucleatum* were quantified relative to *PGT*.

**Table 1:** Clinical characteristics of study patients in relation to *P. micra* and *F. nucleatum* in tumour tissue

	*P. micra*			*P* value	*F. nucleatum*			*P* value
	Total	Positive	Negative		Total	Positive	Negative	
	*n* = 67	*n* = 16	*n* = 51		*n* = 66	*n* = 42	*n* = 24	
Age, *n* (%)								
≤ 59	8 (11.9)	0 (0.0)	8 (15.7)	0.154/0.126*	7 (10.6)	4 (9.5)	3 (12.5)	0.864/0.784*
60–69	12 (17.9)	2 (12.5)	10 (19.6)		12 (18.2)	8 (19.0)	4 (16.7)	
70–79	30 (44.8)	7 (43.8)	23 (45.1)		30 (45.5)	18 (42.9)	12 (50.0)	
≥ 80	17 (25.4)	7 (43.8)	10 (19.6)		17 (25.8)	12 (28.6)	5 (20.8)	
Gender, *n* (%)								
Female	28 (41.8)	5 (31.3)	23 (45.1)	0.393/0.405*	28 (42.4)	21 (50.0)	7 (29.2)	0.125/0.064*
Male	39 (58.2)	11 (68.8)	28 (54.9)		38 (57.6)	21 (50.0)	17 (70.8)	
Location, *n* (%)								
Right colon	35 (52.2)	6 (37.5)	29 (56.9)	0.381/0.569*	34 (51.5)	26 (61.9)	8 (33.3)	0.071/0.004*
left colon	14 (20.9)	4 (25.0)	10 (19.6)		14 (21.2)	6 (14.3)	8 (33.3)	
Rectum	18 (26.9)	6 (37.5)	12 (23.5)		18 (27.3)	10 (23.8)	8 (33.3)	
Stage, *n* (%)								
I	10 (14.9)	1 (6.3)	9 (17.6)	0.346/0.377*	10 (15.2)	3 (7.1)	7 (29.2)	0.112/0.298*
II	27 (40.3)	5 (31.1)	22 (43.1)		27 (40.9)	18 (42.9)	9 (37.5)	
III	25 (37.3)	9 (56.3)	16 (31.4)		24 (36.4)	17 (40.5)	7 (29.2)	
IV	5 (7.5)	1 (6.3)	4 (7.8)		5 (7.6)	4 (9.5)	1 (4.2)	
Tumour grade, *n* (%)								
High grade	19 (28.4)	8 (50.0)	11 (21.6)	0.053/0.034*	18 (27.3)	13 (31.0)	5 (20.8)	0.566/0.430*
Low grade	48 (71.6)	8 (50.0)	40 (78.4)		48 (72.7)	29 (69.0)	19 (79.2)	
Tumour type, *n* (%)								
Non-mucinous	58 (86.6)	12 (75.0)	46 (90.2)	0.201/0.184*	57 (86.4)	35 (83.3)	22 (91.7)	0.469/0.598*
Mucinous	9 (13.4)	4 (25.0)	5 (9.8)		9 (13.6)	7 (16.7)	2 (8.3)	

Fisher’s exact test was used to compare categorical variables

*Calculations based on the relative levels of *P. micra* and *F. nucleatum*, using the Mann–Whitney *U* test to compare two independent samples and the Kruskal–Wallis test to compare several independent samples

**Fig. 1:**
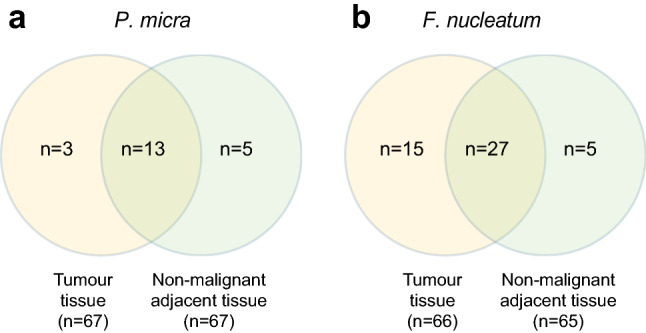
The distribution of *P. micra* and *F. nucleatum* in tumour tissue and adjacent non-malignant tissue of patients with CRC. Venn diagrams are used to illustrate the presence of **a** *P. micra* or **b** *F. nucleatum* in the indicated tissues

*P. micra* was detected in the tumour tissue of 16 (23.9%) patients and in the adjacent non-malignant tissue of 18 (29.6%) patients. Of the 16 patients positive for *P. micra* in tumour tissue, 13 (81.3%) patients were also positive for *P. micra* in non-malignant tissue (Fig. 1a). *F. nucleatum* was detected in the tumour tissue of 42 (63.6%) patients and in the adjacent non-malignant tissues of 32 (49.2%) patients. A total of 27 (42.2%) patients were found to be positive for *F. nucleatum* in both the tumour tissue and the adjacent non-malignant tissue (Fig. 1b).

**Fig. 2:**
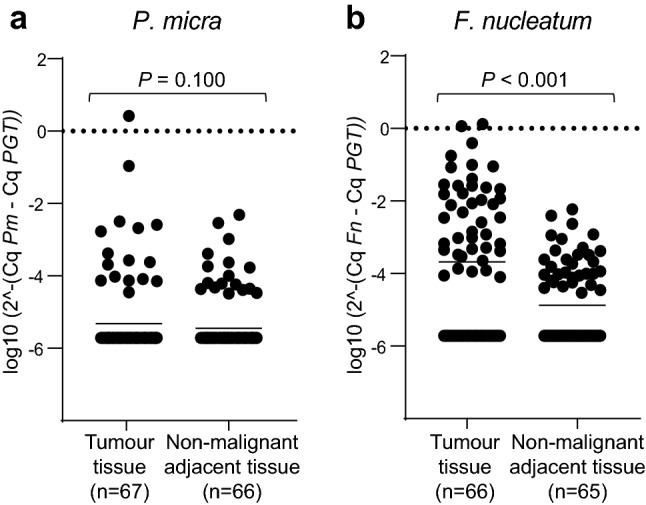
The levels of *P. micra* and *F. nucleatum* in tumour tissue and non-malignant adjacent tissue of patients with CRC. Scatter plots are used to illustrate the relative levels of **a** *P. micra* (*Pm*) and **b** *F. nucleatum* (*Fn*) in the tumour tissues compared to the adjacent non-malignant tissues. Horisontal lines indicate mean relative expression calculated by the 2^−ΔCq^ method using *PGT* as the reference

**Fig. 3:**
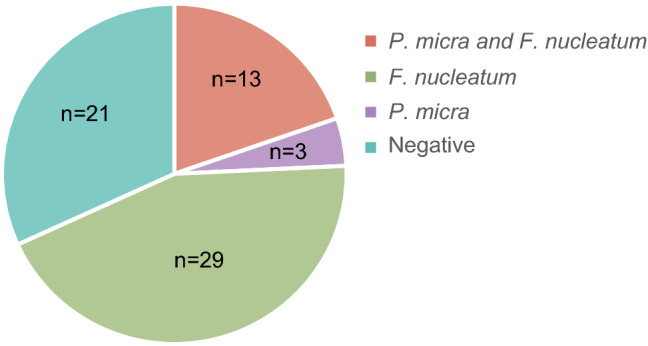
The distribution of *P. micra* and *F. nucleatum* in the tumour tissue of patients with CRC. Circle diagrams are used to illustrate the presence or absence of *P. micra* and/or *F. nucleatum* in the tumour tissues of patients analysed for both microbial markers (*n* = 66)

When comparing the levels of microbial markers between tumour tissue and adjacent non-malignant tissue, no significant difference was found for *P. micra* (*P* = 0.100) (Fig. 2a). In contrast, *F. nucleatum* was found at higher levels in tumour tissue than in adjacent non-malignant tissue (*P* < 0.001) (Fig. 2b). Interestingly, of the 16 patients with *P. micra* positive tumours, 13 patients had tumours that were also positive for *F. nucleatum* (Fig. 3). We further found a trend towards a correlation between the levels of *P. micra* and *F. nucleatum* in tumour tissue (*r*_s_ = 0.232, *P* = 0.060).

### Associations between *P. micra* and *F. nucleatum* and clinicopathological and molecular parameters

We next analysed the associations of *P. micra* and *F. nucleatum* with the clinicopathological and molecular characteristics of the study patients (Tables 1 and 2, respectively). *P. micra* was significantly associated with high-grade tumours (Table 1) and tumours of the CMS1 subtype (Table 2). *F. nucleatum* was significantly associated with right-sided tumours (Table 1) and tumours of the MSI subtype, with 14 of the 16 (87.5%) MSI tumours being positive for *F. nucleatum* (Table 2). Similarly to *P. micra*, the level of *F. nucleatum* was also significantly associated with CMS1 tumours (Table 2). No correlations between *P. micra* or *F. nucleatum* and age, gender, stage, tumour type (mucinous/non-mucinous), and *KRAS* or *BRAF* mutation were found (Tables 1 and 2).

**Table 2:** Molecular characteristics of study patients in relation to *P. micra* and *F. nucleatum* in tumour tissue

	*P. micra*					*F. nucleatum*			
	Total		Positive	Negative	*P* value	Total	Positive	Negative	*P* value
	*n* = 67		*n* = 16	*n* = 51		*n* = 66	*n* = 42	*n* = 24	
*BRAF* mutation status, *n* (%)									
Wild type	44 (66.7)		10 (62.5)	34 (68.0)	0.764/0.691*	43 (66.2)	25 (61.0)	18 (75.0)	0.289/0.093*
Mutant	22 (33.3)		6 (37.5)	16 (32.0)		22 (33.8)	16 (39.0)	6 (25.0)	
*KRAS* mutation status, *n* (%)									
Wild type	45 (72.6)		12 (75.0)	33 (71.7)	1.000/0.967*	44 (72.1)	29 (76.3)	15 (65.2)	0.388/0.399*
Mutant	17 (27.4)		4 (25.0)	13 (28.3)		17 (27.9)	9 (23.7)	8 (34.8)	
MSI status, *n* (%)									
MSS	49 (75.4)		10 (62.5)	39 (79.6)	0.193/0.099*	48 (75.0)	26 (65.0)	22 (91.7)	0.019/0.006*
MSI	16 (24.6)		6 (37.5)	10 (20.4)		16 (25.0)	14 (35.0)	2 (8.3)	
CMS status, *n* (%)									
CMS 1	20 (37.0)		8 (72.7)	12 (27.9)	0.002/0.009*	20 (37.0)	16 (47.1)	4 (20.0)	0.072/0.013*
CMS 2	23 (42.6)		0 (0.0)	23 (53.5)		23 (42.6)	10 (29.4)	13 (65.0)	
CMS 3	6 (11.1)		2 (18.2)	4 (9.3)		6 (11.1)	4 (11.8)	2 (10.0)	
CMS 4	5 (9.3)		1 (9.1)	4 (9.3)		5 (9.3)	4 (11.8)	1 (5.0)	

Fisher’s exact test was used to compare categorical variables

*MSI* microsatellite instability, *MSS* microsatellite stable, *CMS* consensus molecular subtype

*Calculations based on the relative levels of *P. micra* and *F. nucleatum*, using the Mann–Whitney *U* test to compare two independent samples and the Kruskal–Wallis test to compare several independent samples

### *P. micra* is correlated with tumour immune profiles

The tumour infiltration of T helper cells (CD4^+^), cytotoxic T cells (CD8^+^), B lymphocytes (CD19^+^), NK cells (CD56^+^/CD16^+^), and macrophages (CD14^+^), along with markers for their activation/inhibition, was previously analysed in this cohort [23]. Tumour tissue colonisation of *P. micra* was related to several of the analysed immune markers (Table 3). A tendency was found for a correlation between a higher level of *P. micra* and an increased percentage of infiltrating T cells (*P* = 0.051 for CD4; *P* = 0.076 for CD8), and for the fraction of activated CD69^+^ cytotoxic T cells this correlation was significant (*P* = 0.003). However, no correlation was found with the fractions of PD-1^+^ or CTLA-4^+^ cytotoxic T cells (Table 3). A high level of *P. micra* was also positively correlated with the fraction of antigen-presenting human leukocyte antigen (HLA)-DR^+^ B cells (*P* = 0.005), but not with the overall percentage of B cells. Also for macrophages, no correlation was found for *P. micra* to the overall percentage of macrophages. However, a high level of *P. micra* was positively correlated with the fraction of HLA-DR^+^ (*P* = 0.003), as well as with the fractions of CD163^+^ macrophages (*P* = 0.019) and PD-L1^+^ macrophages (*P* = 0.051). No correlation was found between the level of *F. nucleatum* in tumour tissue and the tumour immune activity profile (Supplementary Table 2).

**Table 3:** The correlation between levels of *P. micra* in tumour tissue and immune markers

	*P. micra*	
	*r*_s_	*P* value
T helper cells		
**CD4**	0.265	0.051
CD28	− 0.104	0.449
CD69	0.239	0.079
PD-1	0.198	0.147
CTLA-4	− 0.054	0.694
Treg	0.005	0.972
Cytotoxic T cells		
**CD8**	0.241	0.076
CD28	0.155	0.259
CD69	0.398	0.003*
PD-1	0.150	0.273
CTLA-4	− 0.068	0.620
NKG2D	0.024	0.869
NK cells		
**CD56/CD16**	− 0.042	0.754
NKG2D	0.058	0.663
CD69	0.137	0.301
B cells		
**CD19**	0.103	0.495
CD86	0.120	0.425
CD80	− 0.012	0.939
HLA-DR	0.410	0.005*
CD69	0.123	0.414
Macrophages		
**CD14**	− 0.057	0.699
HLA-DR	0.422	0.003*
CD163	0.341	0.019*
PD-L1	0.286	0.051

Correlations were calculated using the relative levels of *P. micra*. Immune markers in bold are presented as the percentage of positive cells within tumour isolated mononuclear cells. Remaining immune markers (not in bold) are defined as the percentage of cells (in bold) expressing a specific marker

*r*_s_ Spearman’s rank correlation coefficient

*P value < 0.05

**Fig. 4:**
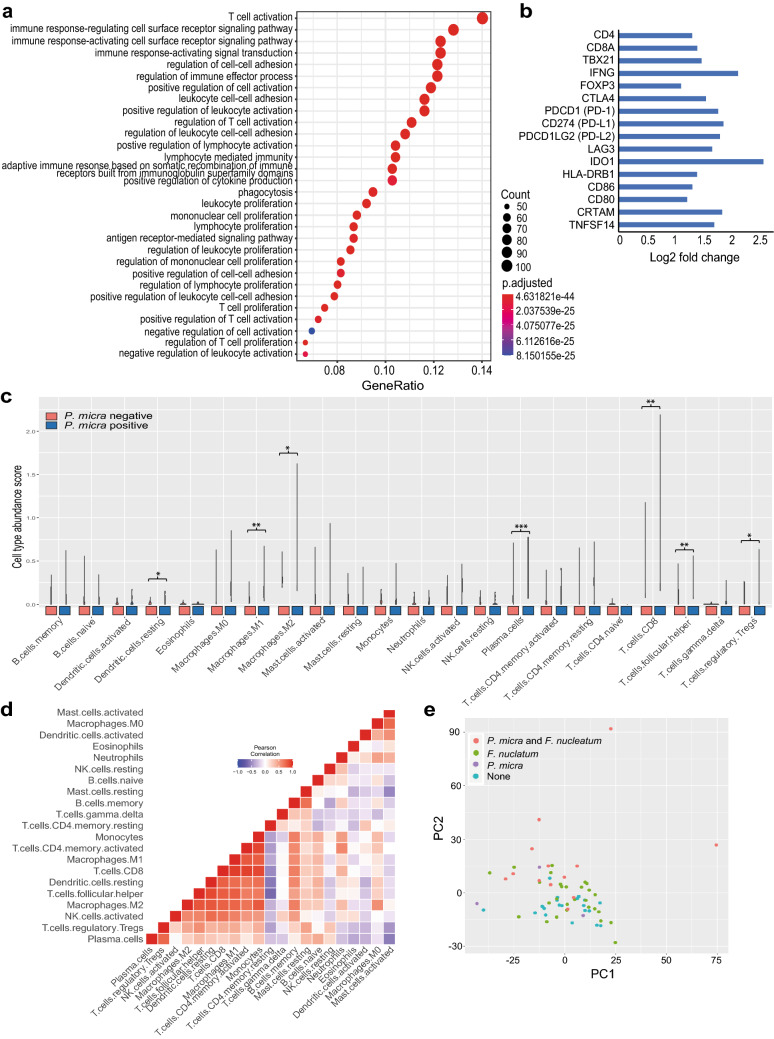
Transcriptomic-based classification of immune profiles according to microbial content in tumour tissues of CRC patients. **a** GO enrichment analysis for biological processes of differentially expressed genes between *P. micra* positive and *P. micra* negative tumours based on clusterProfiler, **b** selected genes included in the top GO term T cell activation, **c** classification of immune cells according to *P. micra* positivity by CIBERSORTx, **d** Correlations of immune cells in *P. micra* positive tumour tissues, and (E) PCA similarity plot according to *P. micra* and *F. nucleatum* positivity. *P values < 0.05, **P values < 0.01 and ***P values < 0.001. Results from figures **a**, **b** are based on the DESeq2 model 1, and results from figure E are based on DESeq2 model 3

GO enrichment analysis of differentially expressed genes in *P. micra* positive tumours compared to *P. micra* negative tumours revealed significantly enriched biological processes related mostly to the immune response and included both innate and adaptive immune events (Fig. 4a). At the top of the list of biological processes that marked *P. micra* positive tumours was T cell activation (Fig. 4a), which included increased levels of CD4, CD8a, T-box transcription factor (Tbx) 21 (expressed by Th1 cells), IFN-γ, immunomodulatory molecules such as CTLA-4, PD-1 and PD-L1, but also costimulatory molecules such as HLA-DR, CD86 and CD80 (Fig. 4b). The complete list of differentially expressed genes according to *P. micra* and *F. nucleatum* can be found in Supplementary Table 3 and 4, respectively. Only one significantly enriched biological process was found for *F. nucleatum* positive tumours and that was bicarbonate transport (Supplementary Fig. 1). We further assessed the role of *P. micra* in CRC immunity using transcriptomic analyses of immune cell abundances by CIBERSORT_x_. Indeed, *P. micra* showed significant associations to T cells (mainly CD8^+^), as well as M1 and M2 macrophages, validating the findings from our flow cytometry analyses (Fig. 4c). Furthermore, the strongest correlations of immune cells found in *P. micra* positive tumours were those of T cells and macrophages (Fig. 4d). Since 13 out of 16 *P. micra* positive tumours were also positive for *F. nucleatum*, we assessed tumour transcriptomes in relation to *P. micra* and *F. nucleatum* positivity based on similarities. Even though the sample size was too low to draw any definite conclusions, samples positive for both *P. micra* and *F. nucleatum* diverged the most from single positive or negative samples. This observation indicates a changed transcriptomic profile for double positive samples. (Fig. 4e).

### *P. micra* is correlated to systemic immune markers

The correlations of *P. micra* to tumour immune activity profiles were not mirrored in the cellular fraction of blood (Supplementary Table 5). To evaluate the correlation of *P. micra* to systemic immune markers, plasma from 63 of the patients was analysed using the OLINK Immuno-Oncology Panel of 92 systemic markers (Supplementary Table 6). A higher level of *P. micra* was correlated with a higher level of interleukin-8 (IL-8) (*P* = 0.010), carbonic anhydrase IX (CAIX) (*P* = 0.015), and tumour necrosis factor (TNF) superfamily member 14 (TNFSF14) (*P* = 0.022). Furthermore, a higher level of *P. micra* was correlated with lower levels of the cytotoxic and regulatory T cell molecule (CRTAM) (*P* = 0.016), and with lower levels of the apoptosis regulators Fas ligand (FASL) (*P* = 0.025) and TNF receptor superfamily member 21 (TNFRSF21) (*P* = 0.040). Interestingly, CRTAM and TNFSF14 were found by GO enrichment analyses to be linked to T cell activation and to be more highly expressed in *P. micra* positive tumours (Fig. 4b). No correlations were found for *F. nucleatum* with the plasma markers that were analysed.

## Discussion

In this study, we investigated the tumour colonisation of two CRC associated microbes from the oral microflora, *P. micra* and *F. nucleatum,* and their relations to tumour molecular determinants and the tumour immune response in CRC. We found associations with tumour molecular traits for both *P. micra* and *F. nucleatum*, including associations with tumours of CMS1 subtype. Furthermore, we found novel associations between *P. micra* and tumour immunity.

Summarising the associations found for the investigated microbes with tumour molecular characteristics, a high level of *P. micra* was associated with high-grade tumours, which has not been previously described. No association with tumour grade was found for *F. nucleatum*. *F. nucleatum* was more often found colonising right-sided tumours, which is in line with previous findings [33]. In a study by Azadeh et al., a difference in crypt mucosa associated bacteria between left and right-sided colon cancers was recognised, with *F. nucleatum* more often being present in right-sided tumours whereas *P. micra* was associated with left-sided tumours [34]. No association of *P. micra* with left-sided tumours was found in the present study, nor in our previous study using faecal samples [12]. Instead, both *P. micra* and *F. nucleatum* were linked to CMS1 tumours, often associated with a right-sided tumour location and an MSI subtype [35]. A similar association of *P. micra* and *F. nucleatum* with CMS1 tumours was previously shown by Purcell et al. [36]. For *F. nucleatum,* a significant association was also seen with tumours of the MSI subtype, an association previously established in the literature [15].

Because tumours of the CMS1 subtype are defined by increased immune infiltration [35], we next investigated the tumour colonisation of *P. micra* and *F. nucleatum* in relation to tumour immune activity profiles. Interestingly, a high level of *P. micra* in tumour tissue was found to be linked to a higher fraction of activated immune components, including CD69^+^ cytotoxic T cells and antigen-presenting HLA-DR^+^ B cells. High levels of *P. micra* were further positively correlated to the fractions of both activated M1 macrophages (HLA-DR^+^) and inhibitory M2 macrophages (CD163^+^ and PD-L1^+^). These findings suggest an initial activation of the macrophage response that is subsequently skewed towards immune suppression, which would be in line with the reported plasticity of macrophage subsets [37]. *F. nucleatum* has been suggested in a previous study to promote M2 macrophage polarisation [38]. Our findings of a potential role of *P. micra* in tumour immunity were strengthened by GO enrichment analyses, demonstrating associations to many immune-related events. A more detailed analysis of immune signatures using transcriptomic data further validated our findings. Surprisingly, *F. nucleatum* was not found to be linked to tumour immune profiles in our study. *F. nucleatum* was shown in a preclinical mouse study to induce a proinflammatory tumour microenvironment and to recruit tumour-infiltrating immune cells, including M2-like tumour associated macrophages and dendritic cells, along with Tregs, and T helper 17 cells, which can promote tumour progression [7]. Mima et al. further concluded that human CRC tumours enriched for *F. nucleatum,* presented with lower densities of CD3^+^ T cells [33]. *F. nucleatum* has also been shown to interact with the immune inhibitory receptors TIGIT and CEACAM1, thus protecting CRC cells from cytotoxicity by NK cells and tumour-infiltrating lymphocytes [39, 40]. The reasons for the discrepancies between these studies and ours are not clear, but may partly reflect the relatively small sample size in our study, or the different methodologies used in the studies. Taken together, our findings suggest a novel role for *P. micra* in tumour immunity in CRC, which may potentially be stronger or at least dissimilar from the effects induced by *F. nucleatum*. Further studies using larger cohorts are needed to address the relative contributions of *P. micra* and *F. nucleatum* in CRC immunity.

In this study, we found a trend towards a correlation between the level of *P. micra* and *F. nucleatum*, which was also evidenced in our previous study [12], as well as in a study by Jun Yu et al. [11], analysing the presence of these bacteria in faecal samples. Additionally, *P. micra* and *F. nucleatum* display synergistic effects in bacterial biofilm formation [41]. In a study by Drewens et al., they found a considerable enrichment of the human oral microbiota in right-sided tumours, including both *P. micra* and *F. nucleatum* [6], and bacterial biofilms in the gut mucosa have been shown to be a consistent feature of these tumours [42]. The tumour colonisation of *P. micra* may thus actually be a marker for a higher degree of dysbiosis associated with CRC disease. Thus, the more specific role of *P. micra* in tumour progression and immunity needs to be addressed in future mechanistic studies.

Recent studies have reported that the effectiveness of immunotherapy might partly depend on the gut microbiota [21]. In melanoma, the gut microbiota has been shown to modulate the response to anti-PD-1 immunotherapy [43]. Here, no significant correlation of *P. micra* or *F. nucleatum* was found with the fraction of cytotoxic T cells expressing the immune checkpoint molecules CTLA-4 and PD-1 using flow cytometry. However, gene expression profiling showed that CTLA-4 and PD-1 expression was significantly greater in tumours colonised by *P. micra* (Fig. 4b). Further studies in larger cohorts are needed to investigate the possible involvement of *P. micra* in response to immunotherapy.

None of the described associations of *P. micra* with cellular immunity found in the tumour compartment, were found in the cellular blood fraction. This finding is supported by transcriptomics studies, suggesting that markers from blood do not in general translate well to tissues, including tumours [44, 45]. However, non-cellular systemic markers may still be potential candidates for future therapeutic decisions. In this study, we found an association between *P. micra* and six of the plasma markers analysed, including a positive correlation with IL8, CAIX, and TNFSF14 and a negative correlation with CRTAM, FASL, and TNFRSF21. The relevance of these markers in clinical decisions regarding *P. micra* needs to be further evaluated. No associations were found for *F. nucleatum* with systemic markers*,* further suggesting a greater impact of *P. micra* on immunity in CRC.

A strength of this study is the very detailed immune and molecular analyses. However, even though our results are biologically and clinically relevant, they should be interpreted with some caution due to the relatively small sample size and the large number of statistical tests performed. Additional limitations of the study include the lack of a validation cohort. Thus, further studies on larger patient cohorts are needed to better elucidate the role of *P. micra* in tumour progression and immunity in CRC.

In conclusion, our findings suggest novel associations between tumour colonisation of *P. micra* and tumour immunity in CRC. Further studies on the role of *P. micra* and *F. nucleatum,* as well as other microbial species, in CRC progression are needed. An improved understanding of the spatio-temporal presence of tumour microbes, and the mechanism by which they regulate tumour progression, may lead to the identification of important biomarkers for CRC disease and outcome, as well as putative targets for future therapy.

## Supplementary Information

Below is the link to the electronic supplementary material.

Supplementary file1 (PDF 1137 kb)

## Data Availability

The data that support the findings of this study are available from the corresponding author upon reasonable request.
