# Supplementary material for: Parvimonas micra is associated with tumour immune profiles in molecular subtypes of colorectal cancer
Source: Cancer Immunol Immunother. 2022 Mar 17;71(10):2565–75. doi: 10.1007/s00262-022-03179-4 (PMC9463256; doi:10.1007/s00262-022-03179-4)
Supplement: Supplementary file 1 — Supplementary file1 (PDF 1137 kb) [file 262_2022_3179_MOESM1_ESM.pdf]

Supplementary Table 1. Primers and probes used for quantitative real-time PCR.

	Forward	Reverse	Probe
P. micra	5’- AAGAATGGAGAGAG TTGTTAGAGAAAGAA - 3’	5’- TTGTGATAATTGTG AAGAACCGAAGA - 3’	5’- FAM - AACTCAAGATCCAGA CCTTGCTACGCCTCA - BHQ1 - 3’
F. nucleatum	5’- CAACCATTACTTTA ACTCTACCATGTTCA –3’	5’- GTTGACTTTACAGAAG GAGATTATGTAAAAATC -3’	5’- FAM - TCAGCAACTTGTCCTTCT TGATCTTTAAATGAACC - BHQ-1 -3’
16S rRNA	5’- GGTGAATACGTTCC CGG - 3’	5’- TACGGCTACCTTGTTA CGACTT - 3’	SYBR Green I
PGT	5’-ATCCCCAAAGCACCT GGTTT-3’	5’-AGAGGCCAAGATAGTCC TGGTAA-3’	5’-FAM-CCATCCATGTCCTCATCTC – BHQ-1 – 3´

Supplementary Table 2. The correlation between levels of *F. nucleatum* in tumour tissue and immune markers.

		F. nucleatum	
		r_s	*P*-value
T helper cells	**CD4**	-0.027	0.844
	CD28	0.018	0.895
	CD69	0.030	0.829
	PD-1	-0.058	0.678
	CTLA-4	-0.079	0.571
	Treg	-0.038	0.777
Cytotoxic T cells	**CD8**	-0.013	0.923
	CD28	-0.112	0.421
	CD69	-0.031	0.825
	PD-1	-0.145	0.295
	CTLA-4	-0.084	0.546
	NKG2D	-0.058	0.692
NK cells	**CD56/CD16**	-0.142	0.284
	NKG2D	0.189	0.151
	CD69	0.065	0.623
B cells	**CD19**	0.142	0.346
	CD86	0.105	0.487
	CD80	0.101	0.506
	HLA-DR	-0.113	0.456
	CD69	0.042	0.783
Macrophages	**CD14**	0.040	0.785
	HLA-DR	0.013	0.932
	CD163	0.211	0.154
	PD-L1	-0.222	0.134

Correlations were calculated using the relative levels of *F. nucleatum.* Immune markers in bold are presented as the percentage of positive cells within tumour isolated mononuclear cells. Remaining immune markers (not in bold) are defined as the percentage of cells (in bold) expressing a specific marker. Abbreviations: r_s, Spearman´s rank correlation coefficient.

Supplementary Table 3. Differentially expressed genes in tumour tissues according to *P. micra*

Gene ID	Gene symbol	Base mean	Log2 fold change	LfcSE	Stat	*P*-value	*P*-adjusted
ENSG00000002549	LAP3	2020.61451836466	0.568687468938137	0.18162404367912	3.13112436777837	0.00174138398301744	0.048560603752026
ENSG00000003393	ALS2	359.207709330485	0.396896569351534	0.122027378862758	3.25252064782868	0.00114386284885945	0.0367942437577118
ENSG00000004399	PLXND1	1484.85012195244	1.00037295143011	0.282579328242183	3.54014908894096	0.000399901052784313	0.0180998693890639
ENSG00000004468	CD38	64.9539424107838	1.50676270181429	0.425578489968854	3.54050483595767	0.000399362316416532	0.0180998693890639
ENSG00000004848	ARX	31.1641205020099	2.83043426182693	0.801692369071786	3.53057403440681	0.00041465895077577	0.0186663769841114
ENSG00000005844	ITGAL	159.759486166112	1.35479016028663	0.350399371807651	3.86641720645075	0.000110445934797443	0.00762260009662794
ENSG00000005961	ITGA2B	8.58294457063292	1.49378237226697	0.453091935782259	3.29686373624808	0.000977709234734282	0.032920603127495
ENSG00000007129	CEACAM21	17.8267704082821	1.37274322336356	0.363858917624384	3.77273486197927	0.000161467850863829	0.00991425265639193
ENSG00000007264	MATK	36.0032880206671	1.17113441176524	0.366453234498271	3.19586321394788	0.00139413187912624	0.0424574558316833
ENSG00000007306	CEACAM7	4645.98756142561	-2.73909591064156	0.656939361301871	-4.16948058221605	0.0000305294624259343	0.00348286798744083
ENSG00000008118	CAMK1G	6.38854040633893	1.46459437498824	0.426323924265999	3.43540273398879	0.000591673816257239	0.0233821032123943
ENSG00000008513	ST3GAL1	679.24189009257	1.36949956110906	0.361139850292329	3.79215852252388	0.000149343528521042	0.00939848543216552
ENSG00000008517	IL32	6678.0839061446	0.767030330859585	0.245344313775993	3.12634240041897	0.00176995343542468	0.0490351685946927
ENSG00000009790	TRAF3IP3	83.7192614611323	1.19614546462781	0.378380606874432	3.16122296675939	0.00157108186442258	0.0455493456721898
ENSG00000010610	CD4	287.793563370153	1.29999346536561	0.308671384680594	4.21157752187109	0.0000253593495655042	0.00310577445855176
ENSG00000011600	TYROBP	328.785959949116	1.29352799515559	0.36415493610989	3.55213637627377	0.000382116757368506	0.0175326676812251
ENSG00000011677	GABRA3	4.28209079018535	3.85234860984152	1.21865928914991	3.16113670501685	0.00157154722900367	0.0455493456721898
ENSG00000013725	CD6	129.703718817511	1.08834266387576	0.303864430699073	3.58167180466601	0.000341402510546473	0.0163089872342124
ENSG00000015285	WAS	123.183139829085	1.52442929027065	0.345186226284956	4.4162517916118	0.0000100442709632526	0.00177674143500766
ENSG00000016391	CHDH	1284.57450587789	-0.718176455842048	0.203906065579191	-3.52209461647	0.000428151304095686	0.0189998795408999
ENSG00000016602	CLCA4	391.409868090609	-3.12503188774441	0.734754591920254	-4.25316414774251	0.0000210770957869223	0.00272844643077963
ENSG00000019582	CD74	14854.6480404132	1.28229613165664	0.372561190366199	3.44184033338588	0.000577771168650422	0.0230591611462654
ENSG00000020256	ZFP64	497.764132251601	-0.472164523205195	0.137951950100392	-3.42267378505041	0.000620084455770186	0.0240188992914145
ENSG00000023445	BIRC3	1034.28361073015	1.52586462877471	0.339549910461872	4.49378598480309	6.99679481596581E-06	0.00145673268068408
ENSG00000023902	PLEKHO1	481.272605008649	1.11480807983382	0.280413595613847	3.97558498329375	0.0000702064669535313	0.00555337623903331
ENSG00000025293	PHF20	977.398158925903	-0.50820746650415	0.162523526788406	-3.12697783851195	0.00176613239894759	0.0490351685946927
ENSG00000025708	TYMP	2344.99801512857	1.38424926812976	0.276432298477587	5.00755257527186	5.51264964545273E-07	0.000275456077483982
ENSG00000026751	SLAMF7	169.108517894869	1.98208704438445	0.383926357559121	5.16267509473931	2.43445478617952E-07	0.000168951162160858
ENSG00000027075	PRKCH	209.722643647883	0.787151436521589	0.231964666645086	3.39341093583859	0.000690280012443317	0.0256637191645867
ENSG00000029153	ARNTL2	683.492920146863	0.981708725874976	0.267774961754923	3.66617072575094	0.000246209585036025	0.0130932240415779
ENSG00000034533	ASTE1	134.251920448953	-0.56426006547293	0.169427230796079	-3.33039773371535	0.000867220128642646	0.0302676034639377
ENSG00000039068	CDH1	10427.1073573756	-0.736502681849722	0.169819105983336	-4.33698362492847	0.0000144451381674559	0.00210168826764897
ENSG00000043462	LCP2	342.765407264269	1.37241833679181	0.345624458066966	3.97083685705454	0.0000716206123269535	0.00559177930742689
ENSG00000044012	GUCA2B	15.7342549263051	-2.15030283940982	0.669458473341342	-3.21200332065023	0.00131812851734851	0.0412166744398437
ENSG00000047457	CP	40.9663498219854	1.74087014006572	0.552731369095855	3.14957724023046	0.00163506879950158	0.046368649613897
ENSG00000049249	TNFRSF9	56.9472610992171	1.12778081722551	0.298934093504419	3.77267378238621	0.000161507397980768	0.00991425265639193
ENSG00000049768	FOXP3	42.3483586190037	1.10412551516569	0.328280802835844	3.36335693597596	0.000770007350534684	0.0276406086864347
ENSG00000056558	TRAF1	227.694482678138	0.836628839167948	0.241780479044435	3.46028282545585	0.000539608305533179	0.0221009408285917
ENSG00000056736	IL17RB	461.68935760598	-0.843506045481734	0.255310700701987	-3.30384133200246	0.000953697924506018	0.0323738980242641
ENSG00000057019	DCBLD2	991.484003810583	1.99174151016897	0.377977359169011	5.26947305666096	1.36815950622506E-07	0.000110657821713214
ENSG00000057657	PRDM1	399.211119869034	0.848114410768352	0.230105523802108	3.68576293499905	0.000228018530338115	0.0124857376831952
ENSG00000059804	SLC2A3	907.238828678117	1.28057759576635	0.404704389424359	3.16422956911292	0.00155494091880899	0.0453309730636218
ENSG00000066294	CD84	99.4912971334111	1.52651845433488	0.353411821503927	4.31937575783079	0.0000156471168109654	0.00222117935457478
ENSG00000066336	SPI1	320.356806357639	1.41541352961028	0.334258318369157	4.23449006898639	0.0000229070907290945	0.00289045835756034
ENSG00000066405	CLDN18	18.0130340810606	2.84496759653402	0.766607907308649	3.71111172923056	0.000206350993308145	0.0115593569883648
ENSG00000066697	MSANTD3	321.560806027978	0.660943805545681	0.169595211215029	3.89718436511555	0.0000973175337524783	0.00686830865331O5
ENSG00000067066	SP100	969.105375869254	0.520604894622057	0.148836422215281	3.49783263312418	0.000469055386504965	0.0201487509159901
ENSG00000070388	FGF22	5.94939885128974	1.05752537862077	0.331000445417815	3.19493644603977	0.00139861635714799	0.0424574558316833
ENSG00000071246	VASH1	296.381356890759	1.07791224939491	0.307587090793092	3.50441316186445	0.000457614784985563	0.0197461965251802
ENSG00000072071	ADGRL1	1141.14217851411	-0.789740042913883	0.236954870227438	-3.33287111657955	0.000859547499619863	0.0302038463157562
ENSG00000072694	FCGR2B	93.1242047977268	1.74316631712887	0.44352974646876	3.93021286848828	0.0000848706902248032	0.00629201579993022
ENSG00000072818	ACAP1	199.83050417959	0.993338794138011	0.311298283404172	3.19095493645339	0.00141803395860904	0.0427957962569227
ENSG00000073861	TBX21	15.9235930285927	1.46672318351543	0.427732750210245	3.42906448663211	0.000605566560279184	0.0237076277771692
ENSG00000074660	SCARF1	155.862861228891	1.0245820744459	0.26992717020331	3.79577229544614	0.000147184546530917	0.00933657741613086
ENSG00000075043	KCNQ2	24.1981910620184	3.3026232167792	0.846138734739399	3.90316987177802	0.0000949410182183645	0.00675785298908153
ENSG00000075213	SEMA3A	369.75528742555	1.31238132933511	0.394301319909193	3.32837163628403	0.000873552507396662	0.0303544309385232
ENSG00000077420	APBB1IP	75.2254407944842	1.20336657743835	0.305604596918034	3.93765862678139	0.0000822805380074133	0.00617326414888052
ENSG00000077984	CST7	89.5268184030592	1.88175698416899	0.40157928437432	4.68589157207364	2.78743813493625E-06	0.000800475337508591
ENSG00000078142	PIK3C3	416.045914690376	0.672541333003545	0.184580225958114	3.64362612253038	0.000268823831669537	0.0137348924739792
ENSG00000079215	SLC1A3	57.0064424021395	1.51918103168242	0.425602663328618	3.56948196658587	0.000357687837507788	0.0167349680380091
ENSG00000079263	SP140	43.4532591365791	1.28007595843239	0.308874880174375	4.14431875363166	0.0000340825395309275	0.00368221921613172
ENSG00000080189	SLC35C2	1294.66156530561	-0.487109514825197	0.15007655011304	-3.24574035356156	0.00117145629347251	0.0374350363080741
ENSG00000080839	RBL1	398.955458230433	-0.899769076063514	0.227774958430547	-3.95025459454916	0.0000780681065978547	0.00598298642711903
ENSG00000081237	PTPRC	449.976357590749	1.32523571137962	0.352894040559605	3.75533604726823	0.000173109052163827	0.0102749009547565
ENSG00000082074	FYB1	168.488789591143	1.24761774609537	0.31201848332972	3.99853794807716	0.0000637349635377277	0.00520377231708036
ENSG00000085265	FCN1	42.0121459111027	1.85981121769941	0.567486024658518	3.27728109043485	0.00104811969351927	0.0344555558191561
ENSG00000086300	SNX10	329.875417378507	1.08678653740173	0.293474302420959	3.70317444640466	0.000212918363275294	0.0118400248872546
ENSG00000086730	LAT2	216.160238575726	1.91005488623369	0.396860297311925	4.81291502115774	1.48744549489155E-06	0.000509073126635213
ENSG00000087128	TMPRSS11E	3.46384357401286	4.02215513442986	0.948973605727453	4.23842676988535	0.0000225091579022637	0.00286453445850951
ENSG00000087589	CASS4	18.3696511910841	1.33433958649224	0.353869365543586	3.77071234872942	0.00016276822210751	0.00996553722974346
ENSG00000088726	TMEM40	6.2705636149449	2.48217683666167	0.659845790118308	3.76175293347201	0.000168726675960751	0.0101354053354664
ENSG00000088827	SIGLEC1	87.0431598335352	1.25697087162213	0.403456844092899	3.11550266162472	0.00183631691461439	0.0498547522664581
ENSG00000089012	SIRPG	32.1881922988234	1.30965113441977	0.410549281624107	3.18999738408717	0.00142274081542955	0.0427957962569227
ENSG00000089041	P2RX7	45.7540708026856	1.21498893527658	0.359499097410267	3.37967172665809	0.000725724540973197	0.0265080437597578
ENSG00000089692	LAG3	65.0047979676006	1.65390142796271	0.360222723554604	4.59133008501614	4.40430127190137E-06	0.00108947587106123
ENSG00000090104	RGS1	693.870413397062	1.4319939912674	0.381327029938751	3.75529107259301	0.000173140141768832	0.0102749009547565
ENSG00000090339	ICAM1	879.985359084657	1.23224476539398	0.26065431998815	4.72750563063753	2.27294762844229E-06	0.000687577357793675
ENSG00000090659	CD209	44.7595284167746	1.17120910387388	0.350487052710697	3.341661538752	0.000832785417670165	0.0295544188566355
ENSG00000091129	NRCAM	55.8478671797979	1.1478428670336	0.361517316861776	3.1750702206956	0.00149800245517346	0.0441159353872439
ENSG00000091138	SLC26A3	1412.84090513449	-3.22163175659498	0.622660492960319	-5.17397810366667	2.2916169160151E-07	0.000163582162942061
ENSG00000091844	RGS17	58.2883845504844	1.75293669921261	0.383547130500221	4.57032932804486	0.00000486958364988	0.00112649701767252
ENSG00000092054	MYH7	2.6260022934299	2.78579879045154	0.66560827076994	4.18534281016232	0.0000284736053563	0.00331635301364921
ENSG00000093072	ADA2	206.041376937805	1.23635193779411	0.317637310584753	3.89233851501278	0.0000992825826751426	0.00692870403786526
ENSG00000093217	XYLB	194.338638984781	-0.52053113845729	0.15668464315819	-3.32215798539847	0.000893241026952515	0.0308668517529483
ENSG00000094963	FMO2	8.42938495183112	2.20306211878391	0.538495975214937	4.09113943313053	0.0000429258938302901	0.00422285555691326
ENSG00000095370	SH2D3C	121.007189398398	1.06782370674314	0.301372063297585	3.54320733998735	0.000395291783505616	0.017956308943826
ENSG00000095627	TDRD1	3.3354345364015	3.3841240557307	0.795529240818593	4.25392792884452	0.0000210052933350375	0.00272844643077963
ENSG00000095932	SMIM24	573.013825346701	-1.31122707247151	0.38413936402957	-3.4134150135433	0.000641541643889683	0.0243590827217931
ENSG00000096968	JAK2	350.086972714881	0.84286081364627	0.222663485133027	3.78535714170968	0.000153487987945922	0.00958685972710 23
ENSG00000096996	IL12RB1	71.4816591222727	1.29169546633843	0.320308926020579	4.03265523189148	0.0000551501 61311963	0.00476772190386881
ENSG00000099250	NRP1	650.774920990859	1.02503539367527	0.295131274207218	3.4731507070158	0.000514386427899546	0.0213664358695024
ENSG00000099381	SETD1A	803.9426703866	-0.490442807356625	0.143007630798713	-3.42948697644629	0.000604723453898505	0.0237076277771692
ENSG00000099822	HCN2	16.6571487155186	1.15079657429995	0.369218173181166	3.11684705112087	0.0018279638230434	0.0497024505499483
ENSG00000099834	CDHR5	2623.59963074877	-1.50394210060156	0.377025004129151	-3.98897177675352	0.0000663603156735959	0.00535977185764146

ENSG00000099985	OSM	149.694142997882	2.01485179137501	0.485047595622194	4.15392594368076	0.000032681898607194	0.00362475419726277
ENSG00000100034	PPM1F	540.602459598699	0.471923291496669	0.146031625348728	3.23165129724264	0.00123077148367499	0.038874329643661
ENSG00000100055	CYTH4	174.951800160283	1.46087030593236	0.371421069584243	3.93319180187493	0.0000838252971544473	0.00627033300630752
ENSG00000100336	APOL4	212.177053484559	1.2865855672272	0.287875637133561	4.46924088484183	7.84976885161084E-06	0.00155649702371941
ENSG00000100342	APOL1	2476.97981514855	1.32261975491439	0.358993801436829	3.68424120310926	0.000229384950231612	0.0124857376831952
ENSG00000100351	GRAP2	58.3295495013398	1.48033377618395	0.396105872430149	3.73721744416956	0.000186068002416929	0.0108361840448125
ENSG00000100365	NCF4	174.526745303315	1.28654757935239	0.31595720109684	4.07190459621166	0.0000466302793111457	0.00437974021920927
ENSG00000100368	CSF2RB	186.645288167779	1.59758597600619	0.335682034696301	4.75922394074964	1.94338724158151E-06	0.000606919835545906
ENSG00000100385	IL2RB	153.933138843744	1.33775111139645	0.313929800983977	4.26130653159852	0.0000203235248670411	0.0026584447396762
ENSG00000100450	GZMH	54.2453642443164	1.89641938768088	0.502163954973754	3.77649444747582	0.000159051103567619	0.0098359722067658
ENSG00000100625	SIX4	70.3404550795158	1.40668552136347	0.298649065367252	4.71016214175541	2.47519797469906E-06	0.000736194597617635
ENSG00000100644	HIF1A	2598.78874589581	0.775212013797425	0.19240176893516	4.02913142684605	0.0000559833148650608	0.0047900244472215
ENSG00000100906	NFKBIA	1904.84926349238	0.991888697932198	0.217884769153116	4.55235444766293	5.30488613595732E-06	0.00118336852875677
ENSG00000100983	GSS	1924.8732973972	-0.647728793131821	0.150869177552935	-4.29331427159503	0.0000176025569074114	0.00245688425572495
ENSG00000100991	TRPC4AP	1833.8929010636	-0.442750354538961	0.106663588293938	-4.15090436784156	0.0000331164125648389	0.00364484780405258
ENSG00000101017	CD40	171.147056184452	1.11453185403303	0.353727119596093	3.15082387605922	0.00162810635028005	0.0463030693713705
ENSG00000101019	UQCC1	894.364843193977	-0.596342208862997	0.186949644923102	-3.18985472857299	0.00142344327127372	0.0427957962569227
ENSG00000101057	MYBL2	1632.22729207001	-1.0079764981554	0.277781845891066	-3.6286622508466	0.000284893674773015	0.0142640953317214
ENSG00000101076	HNF4A	4375.34558504034	-1.0882251145632	0.225348459710794	-4.82907722537708	1.37167208632664E-06	0.000496664571083836
ENSG00000101082	SLA2	34.056040357649	1.27855718975609	0.346997479040718	3.68462962120268	0.000229035446523266	0.0124857376831952
ENSG00000101126	ADNP	2102.8925074901	-0.399479734502291	0.128165484411972	-3.11690574365727	0.00182759994554799	0.0497024505499483
ENSG00000101138	CSTF1	608.031843228667	-0.514730178540841	0.148139250675707	-3.47463738471071	0.000511544340107611	0.0213362667666921
ENSG00000101158	NELFCD	1920.28556613161	-0.705499753274194	0.205634435118371	-3.43084441508098	0.000601705599937392	0.0236368124352764
ENSG00000101161	PRPF6	2523.95121657928	-0.543510474439201	0.173153367010478	-3.13889636582297	0.00169585412069608	0.0477572664603769
ENSG00000101188	NTSR1	223.331682939225	2.73823308579102	0.795191827945471	3.44348745744252	0.000574263230035282	0.0230241777896857
ENSG00000101191	DIDO1	1801.11677003927	-0.620857140109545	0.19280370665249	-3.22015147368811	0.00128122879078904	0.0401633878407444
ENSG00000101210	EEF1A2	108.579057907059	2.77213059534271	0.613190714518005	4.52082937609668	6.15978222172512E-06	0.00131535041903915
ENSG00000101331	CCM2L	35.4847175672537	0.999708779233209	0.317164954816519	3.15201526540517	0.00162147796025514	0.0462454399075507
ENSG00000101333	PLCB4	1632.4272063081	-1.34499381254902	0.343293920046368	-3.91790746648631	0.0000893209791485698	0.00648719576467403
ENSG00000101336	HCK	168.929954255819	1.36745989561575	0.299648124199804	4.56355233081298	5.02952657797849E-06	0.00114234265476559
ENSG00000101337	TM9SF4	2792.44838142254	-0.598190996903285	0.160986019588171	-3.7157946909524	0.000202566019384279	0.0114759850981788
ENSG00000101346	POFUT1	2343.59878197953	-0.98887616705803	0.227146217805159	-4.35347846252172	0.0000133994265017048	0.00205726877171705
ENSG00000101350	KIF3B	1573.81111134097	-0.60825024211731	0.161736596966372	-3.76074588884651	0.000169407457269757	0.0101354053354664
ENSG00000101407	TTI1	533.860315012793	-0.725181220471975	0.185038255013227	-3.91908808489433	0.0000888846285672917	0.00648719576467403
ENSG00000101413	RPRD1B	948.947896298266	-0.756472282930671	0.152840223208924	-4.94943194303383	7.44303979946895E-07	0.000326240186578829
ENSG00000101417	PXMP4	382.652564380462	-0.639702977086813	0.200346085089081	-3.19298965488832	0.00140807990972559	0.0425901555261309
ENSG00000101444	AHCY	6464.7823867281	-0.683220847014799	0.199410777900188	-3.42619819354386	0.00061209357350715	0.023820164860596
ENSG00000101445	PPP1R16B	106.397187949328	1.08993512993735	0.328249983703931	3.32044229717444	0.00089874936051855	0.0309736918875359
ENSG00000101452	DHX35	339.157921894765	-0.698520456138437	0.173497489789978	-4.02611275231712	0.0000567065055826792	0.00483534244190326
ENSG00000101464	PIGU	875.231126459197	-0.76536443971122	0.175296880095882	-4.36610417306109	0.0000126482175110899	0.00200001940694348
ENSG00000101470	TNNC2	69.3279205687405	-1.67906899261909	0.510253914345357	-3.29065382040878	0.000999548327204823	0.0330764457314609
ENSG00000101624	CEP76	143.900205500922	0.540530309308676	0.169616629495127	3.18677662041506	0.00143867840665132	0.043031911545288
ENSG00000101745	ANKRD12	668.295205015723	0.617703575728325	0.181150209858567	3.40989710257912	0.000649873932992377	0.0244893670314956
ENSG00000101916	TLR8	21.201354484292	1.94075692786038	0.383797195340753	5.05672514395862	4.26517264482151E-07	0.000242184257632319
ENSG00000102096	PIM2	494.749935604325	0.982176223545979	0.220845166710351	4.44735213442163	8.69352619306673E-06	0.00163307562712466
ENSG00000102524	TNFSF13B	99.2226328385263	1.30766169074563	0.342960623259169	3.8128624747613	0.000137366555856767	0.00887784921164335
ENSG00000102575	ACP5	594.75560175204	0.987796642382306	0.253320473526228	3.89939521520764	0.0000964332592379744	0.00682518002493358
ENSG00000102755	FLT1	629.847940634323	0.932918857899957	0.295225452710359	3.16002177093867	0.00157757350422892	0.0455654293984454
ENSG00000102924	CBLN1	26.2223127863798	-2.00554240701865	0.586330234488877	-3.42049972703002	0.000625061984590753	0.0240254594200237
ENSG00000102970	CCL17	12.5990663519151	1.84310110873192	0.578255005012747	3.18735003200066	0.00143582895603779	0.0430128904528156
ENSG00000103375	AQP8	38.2011056983619	-2.29734411364829	0.6388159459951	-3.59625355010457	0.000322833147708397	0.0156919520668221
ENSG00000103522	IL21R	56.5634874795183	1.66147997221885	0.371961015415246	4.46681212106067	7.93937965144452E-06	0.00156179450487111
ENSG00000103647	CORO2B	23.6275756995905	2.03147869587052	0.459724293744898	4.41890655662803	0.0000099201524267117	0.00177674143500766
ENSG00000104044	OCA2	13.5060776337285	-3.00237820184025	0.727049683140049	-4.12953649724913	0.0000363495298836646	0.00384812141785372
ENSG00000104327	CALB1	118.106671740445	2.82274883694126	0.583362058083943	4.83875973389939	1.30651874081534E-06	0.000480030356184273
ENSG00000104518	GSDMD	2053.04263021648	0.560839578285553	0.172438011860695	3.25241269157423	0.00114429744635535	0.0367942437577118
ENSG00000104814	MAP4K1	96.3183721141613	1.44179939094772	0.368692668071061	3.91057245182273	0.0000920776347677802	0.0065915977852098
ENSG00000104892	KLC3	29.6259953986915	1.54906416077781	0.437148773104573	3.54356286940154	0.000394759176954653	0.017956308943826
ENSG00000104903	LYL1	69.2090833792205	0.852164932423812	0.261461141002793	3.25924123621379	0.00111710635117638	0.0360126259068267
ENSG00000104951	IL4I1	95.4944666731715	1.39805005660105	0.327450950933765	4.26949456892505	0.0000195916419626496	0.00257619780418335
ENSG00000104972	LILRB1	80.9236388577537	1.71988602394032	0.383801910281124	4.48118151022374	7.42309494454847E-06	0.00153271564434033
ENSG00000105122	RASAL3	173.263840946441	1.28401268308054	0.353851476656368	3.62867691047526	0.000284877500209908	0.0142640953317214
ENSG00000105135	ILVBL	1795.934639547	-0.553928317629387	0.155424249271885	-3.56397615059665	0.000365279158568094	0.0169946638690228
ENSG00000105374	NKG7	104.606482220234	1.74170371138247	0.419236217946453	4.15446861894202	0.0000326044355816944	0.00362475419726277
ENSG00000105398	SULT2A1	7.18365932184504	-3.72286353948346	0.867681000668942	-4.29059013233354	0.0000178198941479069	0.00245973610713429
ENSG00000105639	JAK3	281.258987270895	1.00912102486234	0.292379872707103	3.45140387236316	0.000557678374121138	0.022545366503305
ENSG00000105967	TFEC	59.2201268922069	1.47180132344856	0.396445234924532	3.71249593586044	0.000205225357266921	0.0115593569883648
ENSG00000105971	CAV2	541.785839354113	1.30784744791449	0.328017887999501	3.98712233619553	0.0000668795445907452	0.00537272843104559
ENSG00000105974	CAV1	641.466851560884	0.902004984566301	0.266866390106604	3.37997720710383	0.000724892037779286	0.0265080437597578
ENSG00000105982	RNF32	87.8357641146031	-0.849049008159897	0.235409120128938	-3.60669547422316	0.000310121256818244	0.0152505547330096
ENSG00000106123	EPHB6	205.936888645792	2.37223299434557	0.647366146446822	3.66443782605249	0.000247882443630577	0.0130932240415779
ENSG00000106384	MOGAT3	370.346260450511	-1.11704252902354	0.350589510090738	-3.18618353763221	0.00144163104240338	0.043031911545288
ENSG00000106404	CLDN15	659.475739247808	-1.70392264288766	0.420274970454734	-4.05430435470384	0.0000502837139515497	0.0045849938298011
ENSG00000106809	OGN	13.5693831356516	-2.10363374773004	0.673032036750131	-3.12560715220609	0.00177438413655694	0.0490389527297992
ENSG00000108001	EBF3	14.2218647036427	1.2392701342492	0.356315627917938	3.47801229345633	0.000505146757002471	0.0212110698772675
ENSG00000108576	SLC6A4	140.367023041674	-3.03973304125838	0.611792676516641	-4.96856722536412	6.74494170424694E-07	0.000312065969516492
ENSG00000108622	ICAM2	224.385761991206	0.862683062546644	0.254539225310515	3.38919497179363	0.000700981493238622	0.0259841567167266
ENSG00000108688	CCL7	8.3334175107799	2.72779338689238	0.70646582430316	3.86118237153652	0.000112839635871289	0.00770269252078768
ENSG00000108691	CCL2	208.904042314003	1.43694729859531	0.424408690168747	3.38576313794135	0.000709806211367576	0.0261701212318705
ENSG00000108700	CCL8	25.9769400408376	2.225827132989	0.546415776589143	4.07350451497418	0.0000463109533114072	0.00437974021920927
ENSG00000108950	FAM20A	73.3358188933362	1.93426081258464	0.378259677226463	5.11357918657189	3.16111150212951E-07	0.000200067637467126
ENSG00000109790	KLHL5	254.061443549665	0.977639064271952	0.284893161497859	3.43159891635131	0.000600034261432844	0.0236082771458869
ENSG00000109861	CTSC	2961.02410805087	0.834931582773341	0.202769376560011	4.11764141576988	0.0000382769653174208	0.00398951790871951
ENSG00000109943	CRTAM	15.8923838855688	1.83107829293454	0.505200462324197	3.62445886235058	0.000289567104045785	0.0143604116434913
ENSG00000109956	B3GAT1	13.8600598699255	1.97824671496598	0.505374372982398	3.9144183415784	0.0000906223994595972	0.00656263776260457
ENSG00000110031	LPXN	197.678231835028	1.26272825659316	0.24746246755345	5.10270615611809	3.34830682506904E-07	0.000200067637467126
ENSG00000110077	MS4A6A	223.55936660417	1.1829062858158	0.32637767603474	3.6243480258432	0.000289691301165531	0.0143604116434913
ENSG00000110079	MS4A4A	78.1328995750159	1.31753390678666	0.355823712533547	3.70277151403292	0.000213256932407323	0.0118400248872546
ENSG00000110218	PANX1	283.639141734471	0.602484044528412	0.131187501735981	4.59254148875348	4.37880421324371E-06	0.00108947587106123
ENSG00000110324	IL10RA	190.800468995457	1.3176921228717	0.300097090574542	4.39088603074741	0.0000112889703111912	0.00186783863744901
ENSG00000110665	C11orf21	15.753419233535	1.68542060398024	0.456915466838495	3.68869238689173	0.000225409548991431	0.012404476149784
ENSG00000110756	HPS5	306.354806698546	0.493081438993894	0.127701792956155	3.86119433078898	0.000112834111986228	0.00770269252078768
ENSG00000110848	CD69	71.1068904218046	1.32125336072847	0.400439443177922	3.2995085355301	0.000968542821316363	0.0327000997942811
ENSG00000110852	CLEC2B	145.917928885175	1.25703117861531	0.373575849017852	3.36486200036782	0.000765819513726309	0.0275695024941471
ENSG00000110876	SELPLG	207.206625527864	1.14128867315583	0.312031465215399	3.65760764661342	0.00025458035165726	0.0133622594659768
ENSG00000110944	IL23A	48.618764563023	1.57930807001217	0.420273466213184	3.75781056140019	0.000171406565641475	0.0102205766968654
ENSG00000111058	ACSS3	56.9029056898888	1.7352427195612	0.427145501928962	4.06241599577886	0.0000485674275581149	0.00447752254653854
ENSG00000111252	SH2B3	521.899922465962	0.926698489342175	0.187199993876541	4.9503126049958	7.40943711803241E-07	0.000326240186578829

ENSG00000111537	IFNG	13.8034036256016	2.11192975600396	0.576269120486255	3.66483242104299	0.000247500058570724	0.0130932240415779
ENSG00000111729	CLEC4A	35.1580806314732	1.3189830482656	0.322690121577071	4.08746026007671	0.0000436121352355 25	0.00422498284918415
ENSG00000111817	DSE	209.956969369976	1.05320939609836	0.252149726073793	4.17692064353119	0.0000295482053512448	0.00340391320622509
ENSG00000112096	SOD2	8182.6609070215	0.909942038352914	0.215591066693639	4.22068526450574	0.0000243560793943673	0.00299759747580725
ENSG00000112208	BAG2	341.771440833339	1.24954218162053	0.295720539863393	4.2254155974345	0.0000238500205196424	0.00297934456331373
ENSG00000113088	GZMK	57.5806230403908	1.70347811179255	0.539219403145008	3.20922819486744	0.00133091816689338	0.041512683497708
ENSG00000113231	PDE8B	57.4730775789709	0.906315032820421	0.224329628159708	4.04010402128061	0.0000534274996789516	0.00468362334027694
ENSG00000113263	ITK	59.6302903711057	1.21174796777696	0.368226626035928	3.29076683243087	0.000999146926899358	0.0330764457314609
ENSG00000113303	BTNL8	202.414093634855	-1.63397048126343	0.496167726992586	-3.29318170524188	0.000990604324920845	0.0330136987358669
ENSG00000113532	ST8SIA4	126.550263669048	1.11566441273971	0.268629002815737	4.15317929577761	0.0000327887627514164	0.00362475419726277
ENSG00000113749	HRH2	27.2866994764206	1.35094458783572	0.394246591760655	3.42664874235837	0.000611078981797769	0.0238177804699461
ENSG00000114013	CD86	82.2520247991628	1.3047236686152	0.295460924446277	4.41589246043409	0.0000100594088519721	0.00177674143500766
ENSG00000114113	RBP2	76.6954137730057	-2.7964160106746	0.830005619938831	-3.3691531039039	0.000753995342287713	0.0272222827047922
ENSG00000114650	SCAP	1630.54316228217	-0.429605979578127	0.131353669827658	-3.27060507819682	0.00107317664150045	0.0350945617948261
ENSG00000115008	IL1A	53.2405033312345	1.58152599795509	0.412361261368113	3.83529236647491	0.000125415018156045	0.0082893354857424
ENSG00000115085	ZAP70	146.730459756204	1.39044235924129	0.418171444987815	3.32505333854588	0.000884016211116077	0.0306328169466353
ENSG00000115165	CYTIP	155.923067594132	0.985663129302421	0.310841527748057	3.17095060123794	0.0015194097897179	0.0446599225721318
ENSG00000115232	ITGA4	113.658717481981	1.019788178328	0.290952674282111	3.50499675194325	0.00045661285391695	0.0197461965251802
ENSG00000115295	CLIP4	69.2934151354364	1.1916815419828	0.378500579859123	3.14842725584818	0.00164151573692662	0.0464984457725335
ENSG00000115355	CCDC88A	258.109826428518	0.955069938176478	0.261265626609945	3.65555144229648	0.000256629738898469	0.0133854642936103
ENSG00000115386	REG1A	7928.88062525336	3.7714754021586	0.957226370645574	3.94000365829353	0.0000814803665822723	0.00615016760933985
ENSG00000115415	STAT1	3671.87578380023	1.2288946372996	0.225279388844568	5.45498034064482	4.89782639503179E-08	0.0000489469178613897
ENSG00000115523	GNLY	171.060303904192	1.70379606120296	0.425367582455764	4.00546758021964	0.0000618949229345964	0.00512047269734423
ENSG00000115919	KYNU	309.780794819363	1.6121853981642	0.448676345087558	3.59320346574006	0.000326637330802 54	0.0157847332161908
ENSG00000115956	PLEK	271.362830400208	2.02304163302639	0.409150481945022	4.94449285116136	7.63423428526646E-07	0.000328851223074306
ENSG00000116141	MARK1	71.4273214566746	1.91725712107001	0.536094748731451	3.57634005109501	0.000348438345593983	0.0164793224025849
ENSG00000116701	NCF2	188.16256619012	1.35349181149172	0.339842928790892	3.98269817267414	0.0000681372669316559	0.00545622268275798
ENSG00000116741	RGS2	398.686026071861	0.985788861911988	0.309864488138801	3.18135477257533	0.00146587997486928	0.0435994586811121
ENSG00000116793	PHTF1	198.202316206071	0.572163810601019	0.180064794725287	3.17754401394193	0.00148528143837136	0.0439671462752014
ENSG00000116824	CD2	133.919496172956	1.17136418736793	0.370381599253833	3.16258742261428	0.00156373775830874	0.0454284001785879
ENSG00000117090	SLAMF1	30.9310330310446	1.23731366644418	0.375517559024397	3.29495555323365	0.000984372480496326	0.0330114926882151
ENSG00000117091	CD48	136.09887811974	1.24437477014035	0.398348660741136	3.12383319633903	0.00178511623384395	0.0491693731874959
ENSG00000117115	PADI2	762.54310591012	-1.82259327099648	0.487218628404829	-3.7408119573829	0.000183426721425735	0.0107247177025167
ENSG00000117228	GBP1	1038.03461757356	1.47609541756305	0.301839054294994	4.89033939299461	1.00662263178964E-06	0.000405636448913425
ENSG00000117525	F3	1899.52426779404	1.41554618870492	0.346322299372271	4.08736656712744	0.0000436297460410467	0.00422498284918415
ENSG00000117560	FASLG	15.0569113473255	1.5100550177535	0.478859294635277	3.15344201244676	0.00161357282321713	0.0461252899488063
ENSG00000117834	SLC5A9	59.7350356265547	-1.74723983983459	0.407920437262669	-4.28328585730923	0.0000184153331469154	0.00248696585590559
ENSG00000118242	MREG	240.786619931378	0.648030167876889	0.180796246024776	3.58431207575009	0.000337967797657452	0.0161758380396049
ENSG00000118292	C1orf54	123.248505794131	1.05694432491689	0.316519909956411	3.33926647793638	0.00083999942848884	0.0297259854410272
ENSG00000118503	TNFAIP3	1023.38500219223	0.860326569715588	0.241394420428361	3.56398697280954	0.000365264090430657	0.0169946638690228
ENSG00000118705	RPN2	8252.82416875894	-0.558930901283886	0.165072299361172	-3.38597634761824	0.000709254964321205	0.0261701212318705
ENSG00000118985	ELL2	529.48881998669	0.862406537303118	0.209456244897999	4.11735891533381	0.0000383238992192076	0.00398951790871951
ENSG00000119139	TJP2	2565.57886857086	-0.402163367014919	0.118038822408336	-3.40704319824286	0.000656707324739456	0.0247096021103774
ENSG00000120217	CD274	82.4880889620337	1.85316557173865	0.314271797235625	5.89669702480251	3.70849885904327E-09	9.99982434254387E-06
ENSG00000120279	MYCT1	69.4275804524214	0.923574027145971	0.288028282026609	3.2065393740072	0.00134341918754839	0.0417982378352541
ENSG00000120280	CXorf21	13.6865452148768	1.17045225092854	0.365888630370507	3.19893036780976	0.00137938480832558	0.0423372850751919
ENSG00000121057	AKAP1	3527.07295498283	-0.558852917251017	0.158878366712585	-3.51748906295088	0.000435650303181703	0.0191962736767049
ENSG00000121064	SCPEP1	470.231967171274	0.716494524723709	0.207980142860085	3.44501410024378	0.000571029603271449	0.0229694376377272
ENSG00000121210	TMEM131L	132.064654480329	0.721161977992662	0.204293700943178	3.53002552043073	0.000415519584124966	0.0186714771398888
ENSG00000121552	CSTA	33.8517903913017	1.27407610704196	0.404008966579643	3.15358373807478	0.00161278950799456	0.0461252899488063
ENSG00000121594	CD80	14.0837249243403	1.21318714888696	0.387317367963842	3.13228181649788	0.00173453294283277	0.0485014419087505
ENSG00000121742	GJB6	6.91700003456606	2.91403224712168	0.828749079235337	3.51618158032872	0.000437801454126984	0.0192570977639236
ENSG00000121743	GJA3	12.3021284932774	2.72369293126406	0.608538221375321	4.47579599044478	7.61271511114891E-06	0.00153383930916891
ENSG00000121807	CCR2	17.906785763044	2.01450638925171	0.451372675707426	4.46306676870598	8.07948629172422E-06	0.00156478981017394
ENSG00000122122	SASH3	150.814301578433	1.13644882462329	0.362332636404209	3.13647932988156	0.00170989489484637	0.0479461437181165
ENSG00000122133	PAEP	25.27617240183	3.09026744037651	0.71085663484512	4.34724428090878	0.0000137858653030 14	0.00207149779433149
ENSG00000122188	LAX1	18.7424116070434	1.39969448211953	0.414265911299106	3.37873439243454	0.000728203260938603	0.0265381642260818
ENSG00000122223	CD244	20.7564443611492	1.35596447658133	0.433977979741381	3.12450064261183	0.00178107132814823	0.0491693731874959
ENSG00000122548	KIAA0087	4.34406660055394	1.59605158718777	0.512441690990371	3.1146013590408	0.0018419365987621	0.0498580108163297
ENSG00000122862	SRGN	958.638351560645	1.34882752469129	0.342431555835273	3.93896970564285	0.0000818322609982644	0.00615812412283325
ENSG00000123329	ARHGAP9	163.59258780532	1.29407512050286	0.343361338630232	3.76884341628355	0.000164005727176388	0.00996963281697053
ENSG00000123338	NCKAP1L	188.557829472348	1.3160206317389	0.325200429033405	4.04679857173164	0.0000519228844168965	0.00463300480097051
ENSG00000123364	HOXC13	6.81309292085342	3.5072568308799	1.05781073618663	3.3155806713812	0.000914529412126527	0.0313854434513313
ENSG00000123454	DBH	14.4601432455679	3.15838324854994	0.64159666185747	4.92269276995018	8.53614101449103E-07	0.000361469401874651
ENSG00000123561	SERPINA7	5.93771337337016	-4.49514605423252	1.04920168458092	-4.28434887237911	0.0000183275136364697	0.00248696585590559
ENSG00000123685	BATF3	51.1496649447913	1.73098589590167	0.311728196781114	5.55286917826402	2.81018388825699E-08	0.0000358535908990 19
ENSG00000124126	PREX1	416.50869149599	0.929828400710473	0.272147400966355	3.41663524034693	0.000634001645130426	0.0242200261497532
ENSG00000124217	MOCS3	356.330672907565	-0.833785104218121	0.199623314745231	-4.17679220126285	0.0000295648881584552	0.00340391320622509
ENSG00000124228	DDX27	1945.15706662323	-0.703469514038487	0.21986606832613	-3.19953651508891	0.00137648749324539	0.0423003241466701
ENSG00000124253	PCK1	1692.4226343767	-1.71274629049604	0.519755914946839	-3.29528965662896	0.000983202785866849	0.0330114926882151
ENSG00000124469	CEACAM8	15.3368159254309	-1.72364853636481	0.488979183901748	-3.52499368707512	0.000423492883634771	0.0189276318510396
ENSG00000124491	F13A1	101.201023530457	1.43582702441691	0.438279918889612	3.27605021935434	0.00105269840557603	0.0345606004795157
ENSG00000124508	BTN2A2	208.20813829333	0.644283128670586	0.194967882074796	3.30456032970301	0.000951255001061101	0.03233490468913
ENSG00000125046	SSUH2	66.2463721907777	-1.57829782134168	0.483335736208309	-3.26542753433291	0.00109298968009065	0.035463479254351
ENSG00000125347	IRF1	2076.84221981139	1.0723741221801	0.234745300305732	4.56824533135887	0.00000491824168639 92	0.00112731514030273
ENSG00000125510	OPRL1	10.8082896941993	1.26938227174731	0.335026433911927	3.7889018395516	0.000151314683178235	0.00947480211660404
ENSG00000125538	IL1B	405.823076819825	1.69041762662072	0.453595174455659	3.72670989864325	0.00019399555251488 3	0.0111677055306875
ENSG00000125730	C3	2884.42728064888	1.99278813646977	0.459435636579431	4.33746966453536	0.0000144132419721 26	0.00210168826764897
ENSG00000125735	TNFSF14	31.677888299009	1.69097635119405	0.357277683504584	4.73294703464391	2.21283251056836E-06	0.000682535894370862
ENSG00000125967	NECAB3	574.829143558115	-0.606653269287813	0.184885223640978	-3.28124258575608	0.00103350813692615	0.0340648645025898
ENSG00000125991	ERGIC3	5055.83110149366	-0.654726803933243	0.124264410798431	-5.26881992781727	1.37303573121241E-07	0.000110657821713214
ENSG00000126001	CEP250	891.605566411802	-0.728228307909419	0.19597400683141	-3.7159433523033	0.000202446939620589	0.0114759850981788
ENSG00000126003	PLAGL2	1128.59974526679	-1.11400894332613	0.267949285737266	-4.1575365288283	0.0000321697848210832	0.00362475419726277
ENSG00000126246	IGFLR1	98.0242128337333	0.703259477392337	0.222504034272086	3.16065944463895	0.00157412424624093	0.0455604941032831
ENSG00000126259	KIRREL2	2.32824385216016	2.33756542115755	0.616107426707014	3.79408739422511	0.000148187477003882	0.00934928264006307
ENSG00000126264	HCST	55.7574541803614	1.56221997654956	0.310542570645169	5.03061455730196	4.88910157625965E-07	0.000259892156981428
ENSG00000126860	EVI2A	99.5446139836963	1.44169429462868	0.36559606345812	3.94340759851706	0.0000803319513145282	0.00610034489860843
ENSG00000126882	FAM78A	93.17270193996	1.26712589597207	0.282879914287024	4.47937740353801	7.48610713474873E-06	0.00153305656274231
ENSG00000127863	TNFRSF19	77.6854429708861	1.88016135051245	0.542142143249538	3.46802286803047	0.000524302669935081	0.0215801942432588
ENSG00000127951	FGL2	330.210265046275	1.49684805281144	0.36584350753909	4.09149820063843	0.0000428595272415 35	0.00422285555691326
ENSG00000128284	APOL3	381.819249185522	1.18071155383814	0.325368027311244	3.62884934821418	0.000284687307227329	0.0142640953317214
ENSG00000128335	APOL2	766.248251614979	0.98089551666743	0.261699491362411	3.74817509793723	0.000178125888314035	0.0105144781900598
ENSG00000128340	RAC2	478.36397273984	1.18360177115678	0.26139770779493	4.52797303060259	5.95521800152569E-06	0.00129378405695755
ENSG00000128394	APOBEC3F	66.2268185685745	0.890402604369063	0.277447865599104	3.20920096312322	0.00133076648298912	0.041512683497708
ENSG00000128512	DOCK4	167.91747287122	1.15420105789318	0.282811305449181	4.08117015004049	0.0000448095357815 84	0.00427298260292785
ENSG00000128791	TWSG1	799.220050494071	0.713701494469283	0.228302570828943	3.12612114650267	0.00177128566924713	0.0490351685946927
ENSG00000129173	E2F8	250.257569955611	-0.775854056932856	0.245912341006871	-3.15500252551855	0.00160496716058078	0.0460373129046501
ENSG00000129988	LBP	6.12091822022854	1.9041286368599	0.600842532553761	3.16909761492214	0.00152913029266263	0.0448382187020299

ENSG00000130054	FAM155B	31.9633136517913	-3.12731763953336	0.812534153063408	-3.84884454117132	0.000118676289634582	0.00797045274255485
ENSG00000130561	SAG	11.0999844500696	-1.30331282330458	0.387876775588981	-3.36012080466929	0.000779083979646204	0.0278862953402303
ENSG00000130584	ZBTB46	136.901491884562	0.942008643934604	0.238828112901191	3.94429547045969	0.0000800349283477051	0.00609631905438739
ENSG00000130592	LSP1	460.157806376585	1.08551606775073	0.305068053402258	3.55827513121929	0.000373298183442254	0.017207531024209
ENSG00000130699	TAF4	796.772623537311	-0.677109157884165	0.192333920188998	-3.52048747937338	0.000430754329085429	0.019081500279912
ENSG00000130755	GMFG	201.066139553236	1.13811053352167	0.277054008208332	4.10790134703938	0.0000399270569001574	0.00411222139431798
ENSG00000130775	THEMIS2	207.658706332577	0.99856615175226	0.307299056616944	3.24949305977532	0.00115610899246636	0.0370785970061353
ENSG00000131042	LILRB2	121.911160942016	1.57960097626566	0.359591746429616	4.39276204737596	0.000011191960375168	0.00186413292008799
ENSG00000131043	AAR2	771.910036674505	-0.682711195601748	0.149866642763756	-4.55545799259643	5.22715614389952E-06	0.00117653395584852
ENSG00000131069	ACSS2	2119.97372654082	-0.7215051106016	0.225500231231696	-3.19957592353984	0.00137629931978282	0.0423003241466701
ENSG00000131203	IDO1	285.229772731854	2.56175323920895	0.555429361171157	4.61220349210085	3.98422654145363E-06	0.00102620531867709
ENSG00000131401	NAPSB	46.9962741425642	1.29911616776267	0.388858983537527	3.34084134033463	0.000835249389528596	0.0295998166638049
ENSG00000131446	MGAT1	2363.42870716722	0.562015264399998	0.166000914051482	3.38561548056115	0.000710188208261616	0.0261701212318705
ENSG00000131730	CKMT2	190.58995668599	-3.44355612972424	0.748535493520713	-4.60039124334317	0.000004216981459657	0.00106421277563708
ENSG00000132514	CLEC10A	34.764474762756	2.83719343220312	0.46141795501096	6.14885788771641	7.80428635826058E-10	3.16705823601175E-06
ENSG00000132825	PPP1R3D	298.401831666788	-0.884627903127979	0.242060099938605	-3.65457959966286	0.000257603734047515	0.0134082743571732
ENSG00000132965	ALOX5AP	177.549743173768	1.11883435625579	0.346380866749711	3.23006974015178	0.00123760025611403	0.0390406626246881
ENSG00000133055	MYBPH	2.10611387135794	2.81293567952913	0.722660367535962	3.89247259970868	0.0000992277094742839	0.00692870403786526
ENSG00000133246	PRAM1	26.4620036563387	1.33361863616709	0.405222619659551	3.291076488493	0.000998047720786647	0.0330764457314609
ENSG00000133561	GIMAP6	156.039082384429	1.09792270541371	0.302748542524863	3.62651689833833	0.000287270028049242	0.0143256574466712
ENSG00000133574	GIMAP4	169.343825347576	1.10895448127559	0.276146185289564	4.01582401043399	0.0000592384504828868	0.00501699473513371
ENSG00000133739	LRRCC1	514.318238376947	-0.709020004973408	0.207917627593183	-3.41010049595552	0.000649389460837471	0.0244893670314956
ENSG00000133805	AMPD3	243.523818449295	0.59863177899919	0.191188949163121	3.13110031526164	0.00174152661550654	0.048560603752026
ENSG00000134049	IER3IP1	1068.66933166176	0.781290663087724	0.20259044259843	3.85650306631877	0.000115020655911243	0.00778774001974659
ENSG00000134258	VTCN1	10.9219369121094	3.50926373644012	0.928339230796675	3.78015236243821	0.00015673241731658	0.00974080277173492
ENSG00000134265	NAPG	702.487508643345	0.658996959902065	0.151859914094188	4.33950568082986	0.0000142803575501771	0.00210168826764897
ENSG00000134291	TMEM106C	2408.86935592241	-0.58412984464817	0.167652461530299	-3.48417100063038	0.000493663973225478	0.0209400691121653
ENSG00000134339	SAA2	128.714600224533	2.80497778828743	0.613650404651624	4.57097032288254	4.85471037817733E-06	0.00112649701767252
ENSG00000134460	IL2RA	77.6671652044741	1.41611867645624	0.352817018240996	4.01374821293042	0.0000597620952752654	0.00502725989345869
ENSG00000134470	IL15RA	385.680634231816	0.696796045672953	0.193269037819042	3.60531647249863	0.000311772758834703	0.0152731972680906
ENSG00000134516	DOCK2	129.091607446031	1.27160441104079	0.314004722762744	4.04963466744284	0.0000512976517673621	0.00463300480097051
ENSG00000134575	ACP2	690.886725462205	0.475003053812249	0.139107407210412	3.41464961023798	0.000638641093108543	0.0243228796802193
ENSG00000134594	RAB33A	9.38346154681117	1.09265153005584	0.294514428825216	3.71001018325078	0.000207250910882551	0.0115837958780529
ENSG00000134668	SPOCD1	58.6300102232025	1.22375201968281	0.388312883719116	3.15145871020856	0.00162457130235948	0.0462808317196684
ENSG00000134954	ETS1	1163.54349144475	0.832290921943779	0.223003522653274	3.73218733068098	0.000189824245063373	0.0109850117153476
ENSG00000135046	ANXA1	1890.52602714939	1.50626289562147	0.362555503911898	4.15457186380903	0.0000325897181554904	0.00362475419726277
ENSG00000135047	CTSL	996.685337398703	1.10958725267698	0.271134513673564	4.09239860782554	0.0000426956039955054	0.00422228555691326
ENSG00000135077	HAVCR2	105.890599140165	1.38397269319514	0.302024108837064	4.58232522736045	4.59833987148442E-06	0.00109414212713492
ENSG00000135093	USP30	298.277950386465	-0.580110716633177	0.166992559792464	-3.47387163448558	0.00051300639478375	0.0213615862787954
ENSG00000135220	UGT2A3	299.488953168629	-2.45887487425186	0.773264639834258	-3.17986203892485	0.00147345183651331	0.0436687078095476
ENSG00000135363	LMO2	127.086163922824	1.15766582407059	0.28206776918344	4.10421164893079	0.0000405695929093409	0.0041202874359633
ENSG00000135604	STX11	29.7601188971117	1.45478152334465	0.317048657542674	4.58851185373286	4.46416962744283E-06	0.00109345896051011
ENSG00000135905	DOCK10	143.323529515559	1.20000436732144	0.360179097811784	3.33168797026782	0.000863209824994095	0.0302676034639377
ENSG00000136040	PLXNC1	192.566183225407	1.06146900397834	0.303848626812595	3.49341385910892	0.000476886810834072	0.0203928445450998
ENSG00000136048	DRAM1	520.889162257159	0.91360641106017	0.2564945335149	3.56189427720142	0.000368188656482173	0.0170348618399086
ENSG00000136286	MYO1G	164.668434422172	1.37795200368301	0.330629789129543	4.167658356831	0.0000307744712565473	0.00349486086306172
ENSG00000136404	TM6SF1	23.8148514740917	1.18144815141691	0.375113437578097	3.14957565648642	0.00163507766209747	0.046368649613897
ENSG00000136490	LIMD2	321.385656703352	1.33938569613355	0.296481641324101	4.51760078685408	6.25442602823737E-06	0.00132424220245324
ENSG00000136634	IL10	11.3602996707064	1.57032973482669	0.314598665107183	4.99153336932208	5.99018311759568E-07	0.000287805259634636
ENSG00000136881	BAAT	19.0795575124704	-3.0395009410851	0.698451748089932	-4.35176939480397	0.0000135043259110469	0.00205726877171705
ENSG00000137265	IRF4	63.9595378134197	1.97807970944007	0.428068537494459	4.62094159271322	3.82002318254275E-06	0.0010046258862384
ENSG00000137285	TUBB2B	77.8894516093404	1.52928471735718	0.397737588663837	3.84495899041042	0.00012057260125764	0.00805450767331783
ENSG00000137338	PGBD1	45.0445236206452	0.696257332722805	0.198310828900058	3.51093955173621	0.000446525922630221	0.0194355464303021
ENSG00000137473	TTC29	4.83449765599527	4.56567558197963	1.18609176290824	3.84934431277458	0.000118434431504038	0.00797045274255485
ENSG00000137496	IL18BP	180.131875169936	1.45574829647096	0.252962172861518	5.75480626215171	8.67412149689818E-09	0.000018059520956542
ENSG00000137672	TRPC6	32.6287103593502	1.51301866594665	0.315358842143029	4.79776833167224	1.60443193705937E-06	0.000537042885265508
ENSG00000137710	RDX	375.453631714147	1.15416780318749	0.311877493254656	3.70070886213351	0.000214998034767372	0.0119102237264479
ENSG00000137841	PLCB2	236.543914983676	0.980954071101071	0.307472411394529	3.19038077807369	0.00142085451274244	0.0427957962569227
ENSG00000138074	SLC5A6	1286.65432923492	-1.0640102798693	0.23018740967231	-4.62236523441489	3.79389279385863E-06	0.0010046258862384
ENSG00000138100	TRIM54	58.6334184132039	-5.32281541023161	0.971282903985756	-5.48019056897729	4.24867960880367E-08	0.000045879821239805
ENSG00000138166	DUSP5	624.518159593747	1.0612359693712	0.307607302607992	3.44997001167952	0.000560648829478297	0.0226288374082161
ENSG00000138411	HECW2	128.641982913435	0.979831472884737	0.306844598621362	3.19324986422141	0.00140681159604422	0.0425901555261309
ENSG00000138755	CXCL9	666.41229194507	1.99354740411447	0.520079148043237	3.83316157091677	0.000126506802866242	0.0083394352580744
ENSG00000138964	PARVG	128.873970279439	1.22494513505098	0.344870171617499	3.55190223992345	0.000382456927884552	0.0175326676812251
ENSG00000139209	SLC38A4	36.3630007491583	-1.85634897123091	0.586443266853464	-3.16543658381666	0.00154850412605304	0.045248920567613
ENSG00000139517	LNX2	955.88777611988	-0.747254296106133	0.21364421074808	-3.49765759385478	0.00046936331384511	0.0201487509159901
ENSG00000139572	GPR84	15.8845023854672	1.78775613863796	0.487402470446868	3.66792588679102	0.000244526035308754	0.0130741668007767
ENSG00000139626	ITGB7	144.611096686145	1.61882505642752	0.299852207423563	5.39874316863311	6.7109362887893E-08	0.0000620985304589303
ENSG00000140030	GPR65	42.8131039442892	1.03197063248024	0.268889680114585	3.83789601758042	0.000124093005869007	0.00825622677011305
ENSG00000140093	SERPINA10	40.4472471323771	-2.59484789631736	0.616873653845083	-4.20644953815617	0.0000259413973895731	0.00314773102583305
ENSG00000140105	WARS	3572.72734941509	1.38862904495885	0.26289400385372	5.28208717050663	1.27720398361682E-07	0.000110033325264423
ENSG00000140368	PSTPIP1	88.3465589652562	1.18523670490564	0.325967790524154	3.63605466355982	0.00027684560827358	0.0139731528830447
ENSG00000140379	BCL2A1	141.796807154197	2.08045771321167	0.429266120530034	4.84654533333036	1.25629891283498E-06	0.000475566243004078
ENSG00000140511	HAPLN3	354.641711401491	1.24984801686272	0.347594363492404	3.59570852733356	0.000323509863807844	0.0156943115288838
ENSG00000140749	IGSF6	182.497033047922	1.12095599807779	0.345386546846288	3.24551146624903	0.00117239842978644	0.0374350363080741
ENSG00000140992	PDPK1	1520.95665350913	-0.755147655848831	0.182288688804389	-4.14259195565978	0.0000343402608612989	0.00368221921613172
ENSG00000141506	PIK3R5	90.9879191805918	1.31056397327427	0.39264612985537	3.33777382132103	0.000844524638417554	0.0298438522860314
ENSG00000141837	CACNA1A	49.6533486535946	0.94236314613013	0.296697761826529	3.17617207601689	0.00149233402417638	0.044035814498124
ENSG00000141968	VAV1	115.955927933068	1.03350564936978	0.316738729449108	3.26295950977541	0.00110255271139519	0.03568157634909
ENSG00000142149	HUNK	323.971109616046	-1.6719069735652	0.476578868135836	-3.50814332180728	0.000451245876947314	0.0195727899125897
ENSG00000142512	SIGLEC10	76.6037857693469	1.42024590569316	0.371715949576342	3.82078279748788	0.000133028770957154	0.00867778280311625
ENSG00000142677	IL22RA1	725.102888870724	-0.889651769956914	0.257254537870188	-3.45825491484949	0.000543686691250124	0.0221933492328264
ENSG00000143119	CD53	277.133393462077	1.39813120335527	0.316673446133528	4.41505664723696	0.000010098354297594	0.00177674143500766
ENSG00000143158	MPC2	2144.76524703302	-0.668649658681377	0.18148670085921	-3.68429011886711	0.000229340907656243	0.0124857376831952
ENSG00000143226	FCGR2A	411.379092045744	1.2424577829196	0.304225236756679	4.08400629798284	0.0000442658301803808	0.00425360577394859
ENSG00000143258	USP21	400.588781042514	-0.528661722392481	0.139307067659373	-3.79493827036212	0.000147680194994056	0.00934086580185186
ENSG00000143315	PIGM	445.399877105236	-0.566669291275189	0.171814589041057	-3.29814420555274	0.000973261366037879	0.0328150633860869
ENSG00000143382	ADAMTSL4	214.657162166519	2.09666427894045	0.370316177818062	5.66182199031702	1.4977415014266E-08	0.0000220115139244954
ENSG00000143546	S100A8	191.321529241046	1.65875398513738	0.503769877692717	3.29268195378122	0.000992366624665227	0.0330136987358669
ENSG00000143556	S100A7	3.79736179673215	3.99843664388257	0.97995688980614	4.08021688043192	0.0000449937008571252	0.00427423050271641
ENSG00000143603	KCNN3	56.4775297335036	1.70520204936483	0.333066851237704	5.119699072508	3.0602358027337E-07	0.000200067637467126
ENSG00000143627	PKLR	38.358816731414	-4.00418187538307	0.731530762762776	-5.47370265094588	4.40728350046158E-08	0.000045879821239805
ENSG00000143702	CEP170	429.249981456163	0.947737293626431	0.235152975581099	4.03030108925145	0.0000557054517271048	0.0047900244472215
ENSG00000144354	CDCA7	2568.14179500797	-0.779074129363462	0.227730802090078	-3.42103098137469	0.000623842251731005	0.0240254594200237
ENSG00000144834	TAGLN3	8.30763070793649	2.56524087657903	0.779239394066144	3.29198048265163	0.000994845155965983	0.0330521427881039
ENSG00000144837	PLA1A	50.357181961616	1.70202188058425	0.419779921089821	4.05455762668569	0.0000502292672332923	0.004584993298011
ENSG00000144843	ADPRH	100.543061497076	0.692483100192857	0.22174140512095	3.12293096463035	0.00179059742534366	0.0492148361658812
ENSG00000144847	IGSF11	2.3271145646873	2.64539737220752	0.750808021100514	3.52340052032206	0.000426046999761913	0.0189401392207324

ENSG00000144852	NR1I2	664.668614505302	-1.16717150260422	0.275428846952038	-4.23765163133935	0.0000225869872048666	0.00286453445850951
ENSG00000145198	VWA5B2	17.2136137208022	1.57646151597089	0.497344021888829	3.16976066181263	0.00152564548102295	0.0447905131584928
ENSG00000145416	MARCH1	106.985062851582	0.997793993119655	0.268823073726945	3.71171261189119	0.000205861645429956	0.0115593569883648
ENSG00000145632	PLK2	721.66786205962	1.21651939305149	0.346192029752677	3.51400173458812	0.000441409930433935	0.0193816971914964
ENSG00000145649	GZMA	152.451845600637	1.59599502396417	0.388631372192732	4.1067066072388	0.0000401340479573676	0.00411222139431798
ENSG00000145681	HAPLN1	67.9407699898941	1.86310793590669	0.440333713934404	4.23112715867183	0.0000232523168283779	0.00291927579718703
ENSG00000146070	PLA2G7	95.8306274166739	1.13029461749953	0.300548898632015	3.76076779067966	0.000169392623767901	0.0101354053354664
ENSG00000146112	PPP1R18	761.36001456683	0.929943401869121	0.264842141653198	3.51131204446629	0.000445900655274234	0.0194355464303021
ENSG00000146192	FGD2	87.4472059289136	1.09186681548447	0.320001133771746	3.41207170929367	0.000644711507424579	0.0244179746381093
ENSG00000147113	DIPK2B	124.342041483905	1.0107493763977	0.315534448981396	3.20329326848649	0.00135865547111964	0.0420823418223686
ENSG00000147443	DOK2	116.474767847135	1.80087201790999	0.366862262465844	4.90885054735673	9.16117577077732E-07	0.000375217730257542
ENSG00000147488	ST18	36.9029657010024	2.74709600760401	0.601045695004733	4.57052771600398	0.0000048649757139263	0.00112649701767252
ENSG00000147614	ATP6V0D2	12.8982652028903	1.26813198213725	0.338373048175454	3.74773342314102	0.000178439751544402	0.0105144781900598
ENSG00000149474	KAT14	388.04877842564	-0.652466623392727	0.199244108419316	-3.27470975462717	0.00105770584148884	0.0346794261729096
ENSG00000149636	DSN1	541.897133517108	-0.91913414616315	0.196552693792605	-4.67627346350687	2.92134984095875E-06	0.000829397777574015
ENSG00000150337	FCGR1A	101.797910358595	1.74422061545445	0.459163493917877	3.79869183538883	0.00014546181856205	0.00927096447692414
ENSG00000150630	VEGFC	58.224728862988	1.14041628540305	0.364104287350475	3.13211441068619	0.00173552229692695	0.0485014419087505
ENSG00000150637	CD226	57.9914496161184	1.78181066042652	0.37278536895374	4.77972262008928	1.75537206073836E-06	0.000569561241110222
ENSG00000150681	RGS18	18.3917757740765	1.18919262257125	0.320333568172329	3.71235718240901	0.000205337930850671	0.0115593569883648
ENSG00000151117	TMEM86A	60.2357472748941	0.926998320925926	0.249124434601829	3.72102528765406	0.000198415566373543	0.0113178413476635
ENSG00000151702	FLI1	140.910093578754	1.08476791050449	0.308372521255901	3.51771910832582	0.000435272840664506	0.0191962736767049
ENSG00000151743	AMN1	122.752135967215	0.776136749757514	0.189552913615432	4.09456512671788	0.0000422961513507561	0.00422228555691326
ENSG00000152128	TMEM163	33.2892272819483	2.14133747063525	0.441123840285185	4.85427735950722	1.20826529437437E-06	0.000464420001763836
ENSG00000152207	CYSLTR2	9.54953999976848	1.59707323772751	0.422305765248131	3.78179359400677	0.000155702442858439	0.00970092227524998
ENSG00000152315	KCNK13	9.27445496994602	1.446124411158	0.344195349452328	4.20146412047409	0.0000265194281860542	0.00317015020957119
ENSG00000152689	RASGRP3	164.307469668389	1.13706768845961	0.251507498614663	4.52100909405379	6.15455438635868E-06	0.00131535041903915
ENSG00000152782	PANK1	459.355761089422	-0.6601235456336	0.207569874101258	-3.18024736726282	0.00147149382280918	0.0436624722910505
ENSG00000153237	CCDC148	13.0233754459797	1.38426785176374	0.443785216091191	3.11922930636628	0.0018132478637973	0.0496190412147991
ENSG00000153283	CD96	105.742581288504	1.43693346268932	0.361945955541454	3.9700221557656	0.0000718659488009007	0.00559345440760655
ENSG00000153563	CD8A	180.462694222882	1.39098053357043	0.443875567782429	3.13371727243215	0.00172607078559815	0.0483454624522243
ENSG00000153707	PTPRD	710.308895964661	-1.65687222157949	0.531587493022418	-3.11683823138709	0.00182801850870717	0.0497024505499483
ENSG00000154102	C16orf74	55.8613545842006	1.61687074999209	0.433241579830913	3.7320304081227	0.000189942565683245	0.0109850117153476
ENSG00000154451	GBP5	277.754756698464	2.15881743802595	0.439770063244585	4.9089686144127	9.15566279177902E-07	0.000375217730257542
ENSG00000154589	LY96	67.3218520689726	1.45170335394016	0.418006844482414	3.47291766415377	0.000514833269029796	0.0213664358695024
ENSG00000154930	ACSS1	1344.50046694507	-0.839669433318093	0.252738190368146	-3.32228948895696	0.000892820119675152	0.0308668517529483
ENSG00000155269	GPR78	14.2467037271578	2.43199882556801	0.670746847815005	3.62580731238675	0.000288060096830982	0.0143364411538352
ENSG00000155307	SAMSN1	133.952197523294	1.26213299941597	0.346758978283056	3.63979904908389	0.000272850891457806	0.0138837203099426
ENSG00000155659	VSIG4	110.069006926719	1.75506730182255	0.422292063419907	4.15605088006921	0.0000323795744587783	0.00362475419726277
ENSG00000155850	SLC26A2	2039.01207822878	-2.5853435792805	0.493829668030666	-5.23529416446472	1.64722304713997E-07	0.000126717885508766
ENSG00000155926	SLA	188.308718013544	1.08909968467467	0.312005172519327	3.49064624756246	0.000481853806894452	0.0205437466065717
ENSG00000155962	CLIC2	92.1614920745493	1.23712680988692	0.266164123593876	4.64798483425429	3.35193551143121E-06	0.000920187855501581
ENSG00000156076	WIF1	47.0975603181002	-5.45048370918534	1.23787818915365	-4.40308566460154	0.0000106722000784999	0.00183751667366846
ENSG00000156103	MMP16	30.4793754485187	1.22545282024473	0.355708788546988	3.4451013292376	0.000570845354037332	0.0229694376377272
ENSG00000156273	BACH1	863.897683219899	0.54061190275045	0.163304546949611	3.31045223693166	0.000931453472210776	0.0318360558632408
ENSG00000156414	TDRD9	6.2089414004855	1.59918295207168	0.491848876520174	3.2513705498036	0.00114850063229004	0.0368819277598128
ENSG00000156574	NODAL	5.86860374749638	-2.14822307226163	0.683841250411361	-3.14140609530264	0.00168138717635015	0.0474664149309967
ENSG00000156587	UBE2L6	1198.8942396326	0.890458598448946	0.281449603059868	3.16382964753918	0.00155707905709044	0.0453403999561159
ENSG00000157017	GHRL	11.1335356636212	2.12666724839568	0.491945370338739	4.32297441264936	0.0000153939593180191	0.00220859202853756
ENSG00000157388	CACNA1D	332.845515083291	-0.747809109738568	0.234057274218988	-3.19498341691745	0.00139838875315835	0.0424574558316833
ENSG00000158122	PRXL2C	160.839270239884	0.683126490108203	0.198674240465058	3.43842507468073	0.000585108431959349	0.0232037286731308
ENSG00000158296	SLC13A3	87.2368634410105	-2.858975730911	0.590272741646333	-4.84348256186964	1.27582923274978E-06	0.000475751008224187
ENSG00000158428	CATIP	4.05121219376266	1.63376168003297	0.404582671970259	4.03814051668795	0.0000538765784224236	0.00469113593938469
ENSG00000158480	SPATA2	636.831371269136	-0.65137529488489	0.177998222278405	-3.65944831665837	0.000252758812492372	0.013294581413283
ENSG00000158485	CD1B	3.08862028708533	2.09794774475779	0.586430213078644	3.57748918450838	0.000346910568821958	0.0164775924932468
ENSG00000158488	CD1E	5.47573526538685	1.58253738250576	0.484173272591923	3.26853519615813	0.00108105731078407	0.0352139972002989
ENSG00000158517	NCF1	112.0380507819	1.42838120945388	0.353727147404073	4.03808760491372	0.0000538887293709337	0.00469113593938469
ENSG00000158683	PKD1L1	63.5119627654587	-1.82641038658641	0.522866550764029	-3.49307176738998	0.00047749816117849	0.0203928445450998
ENSG00000158714	SLAMF8	159.333272914868	1.64855200020983	0.354753220280232	4.64703885959818	3.36733751016429E-06	0.000920187855501581
ENSG00000158764	ITLN2	8.81766479240232	3.53840562665057	1.02884834084095	3.43919068164934	0.000583456115790101	0.0231749882303655
ENSG00000158869	FCER1G	265.26258168559	1.3879631447226	0.318496239259129	4.3578635275293	0.0000131338233864723	0.00203810834464363
ENSG00000159189	C1QC	946.326491641526	1.28868601083916	0.341866965084531	3.76955407352833	0.000163539475832325	0.00996553722974346
ENSG00000159339	PADI4	7.66015258636288	2.0946445868872	0.605901292439315	3.45707232023604	0.000546078276829366	0.0221933492328264
ENSG00000159753	CARMIL2	115.391050501328	1.38308676674102	0.398894162955418	3.46730259599112	0.000525709720122452	0.0216025191571371
ENSG00000160219	GAB3	36.882496318312	1.37555324176281	0.349940734631099	3.93081772321504	0.0000846574376609066	0.00629201579993022
ENSG00000160255	ITGB2	685.687449851159	1.31870151039638	0.367347924881606	3.58978891964993	0.000330945839452559	0.0159313118552654
ENSG00000160326	SLC2A6	96.7207820086815	0.986638103057929	0.250920625501263	3.93207254719266	0.0000842166404043332	0.00628080162346824
ENSG00000160396	HIPK4	1.17965282666987	2.20937926133151	0.508337786659561	4.34628178213939	0.0000138464670050176	0.00207149779433149
ENSG00000160593	JAML	122.45957658582	1.35286549253773	0.330561019200815	4.0926346839942	0.0000426499368965177	0.00422228555691326
ENSG00000160791	CCR5	84.169612614775	1.44091576880336	0.36987030424517	3.8957325101943	0.0000979023912757884	0.00687076781919746
ENSG00000160867	FGFR4	1730.46101926784	-0.851032907427449	0.266346015074933	-3.19521546882548	0.00139726481410027	0.0424574558316833
ENSG00000160883	HK3	112.556532833222	1.81820045259677	0.412977158646397	4.40266589696202	0.0000106928767856811	0.00183751667366846
ENSG00000160886	LY6K	8.81267865144248	2.40251759409679	0.600262856370549	4.00244254429378	0.0000626918951638662	0.00515228391044056
ENSG00000161267	BDH1	1178.39698452926	-0.56603767718999	0.181421314345172	-3.12001750859906	0.00180840290632017	0.0495407217231395
ENSG00000161642	ZNF385A	254.498747123818	1.31016286944396	0.384022782989208	3.41168005514083	0.000645638452824163	0.0244179746381093
ENSG00000161929	SCIMP	35.4198112372914	1.20145652028675	0.318717680135661	3.76965758465408	0.000163471667913767	0.00996553722974346
ENSG00000161944	ASGR2	4.25250131348366	1.29528505410231	0.378176360820046	3.42508202071009	0.000614613841224058	0.0238810454263482
ENSG00000162460	TMEM82	34.2345689980551	-2.11986827966495	0.549193503376893	-3.85996605318572	0.000113402777968243	0.0077200408849008
ENSG00000162511	LAPTM5	1316.21080769543	1.13276136634069	0.278788572154184	4.06315566519786	0.0000484137197757213	0.00447752254653854
ENSG00000162618	ADGRL4	226.863435304919	0.977426195902049	0.301829761026011	3.23833604936597	0.00120229101203616	0.0381752289520448
ENSG00000162620	LRRIQ3	13.2058466130555	-1.28721916384205	0.375498588393323	-3.42802663879451	0.000607985797836935	0.0237342455830594
ENSG00000162654	GBP4	696.651349802585	1.56740677250524	0.364266012008539	4.30291797981005	0.0000168563264052392	0.00237931332716665
ENSG00000162946	DISC1	71.1803354362913	1.13374959230286	0.256585959170361	4.41859560814902	9.93443315095606E-06	0.00177674143500766
ENSG00000163050	COQ8A	1278.55381305258	-0.66709690886735	0.179167767426033	-3.72330870921162	0.000196628852445936	0.0112415909599754
ENSG00000163075	CFAP221	2.90134619800003	2.63572513090665	0.834842523361678	3.15715246546537	0.00159318021062653	0.0458043893927425
ENSG00000163121	NEURL3	35.3428644286913	1.23986809397184	0.376493419119856	3.29319991002851	0.000990540183160442	0.0330136987358669
ENSG00000163154	TNFAIP8L2	33.267610469911	1.33272802193322	0.323515990746243	4.11951204903061	0.0000379675571306219	0.00398563633340949
ENSG00000163216	SPRR2D	24.1020309246552	6.23574727540019	1.15355859711271	5.40566148179026	6.45696465252325E-08	0.0000620464634148619
ENSG00000163219	ARHGAP25	85.5191421719612	0.904152638930509	0.215436390160411	4.19684268872725	0.0000270661794155026	0.00320484088396643
ENSG00000163220	S100A9	786.237538774838	1.99571753965206	0.419282398163225	4.75984097685669	1.93745550391903E-06	0.000606919835545906
ENSG00000163421	PROK2	17.9762061877846	2.99879389419991	0.625447134555945	4.79464007190471	1.62967162489515E-06	0.000537042885265508
ENSG00000163508	EOMES	17.4820281056536	1.64637330347994	0.468739724572379	3.51234004112174	0.000444179294266481	0.0194349833414251
ENSG00000163536	SERPINI1	128.512736891845	2.35450270676851	0.428382017734353	5.4962687724875	3.87911147870519E-08	0.0000440526005381684
ENSG00000163563	MNDA	77.5972404002405	1.40289956279869	0.391824360783733	3.58042965984194	0.000343029680197721	0.0163554456680532
ENSG00000163564	PYHIN1	17.4198047786852	1.04874376339591	0.314396952705626	3.33573132427228	0.000850753414914981	0.0299791584178221
ENSG00000163565	IFI16	916.284179353792	1.10410873497384	0.331961037523349	3.32601905094414	0.000880959071806734	0.0305692797916937
ENSG00000163599	CTLA4	41.4824044609635	1.54537490747687	0.380752893729849	4.05873450451921	0.0000493393701423697	0.00453196626337119
ENSG00000163638	ADAMTS9	228.527801858327	0.867867274854925	0.260610358666164	3.33013345784402	0.000868034367761069	0.0302676034639377
ENSG00000163661	PTX3	22.0846007986276	1.70586510910814	0.453579429300184	3.76089610531958	0.000169305744377739	0.0101354053354664

ENSG00000163808	KIF15	364.31252762677	-0.733761590691521	0.200001141549772	-3.66878701294271	0.000243703997984284	0.0130658812996552
ENSG00000163823	CCR1	93.002020078208	1.10663012679433	0.313159585070022	3.53375780130403	0.000409696321023185	0.0184762687444824
ENSG00000163874	ZC3H12A	1073.58180108101	0.841934432269383	0.267018456698601	3.1530945897807	0.00161549450417O3	0.0461274453625036
ENSG00000164047	CAMP	1.90823631557395	2.79871898494695	0.681525348102219	4.10655156516231	0.0000401609838381999	0.00411222139431798
ENSG00000164082	GRM2	9.12701070116833	2.01345724666366	0.480135716827753	4.19351690802453	0.0000274662594164559	0.00323687276066384
ENSG00000164543	STK17A	460.415776481219	0.572345080329598	0.177433226613812	3.22569279301515	0.001256681659005	0.0395926034912746
ENSG00000164674	SYTL3	50.4040025127178	0.778331809883347	0.216418347702344	3.59642247594388	0.000322623673591125	0.0156919520668221
ENSG00000164683	HEY1	95.5515987752152	1.1630439785595	0.275493008351304	4.22168237778435	0.0000242485641570412	0.00299759747580725
ENSG00000165046	LETM2	77.4594196196562	1.80246324017131	0.454109525229992	3.96922579251871	0.0000721065311623881	0.00559475023155622
ENSG00000165168	CYBB	388.910118023314	1.45308975925703	0.301463515214252	4.82011814339892	1.43473230120713E-06	0.000499900661140903
ENSG00000165178	NCF1C	64.5632486738736	1.57319136405649	0.359804245159776	4.3723535372905	0.0000122914272933976	0.0019559810159124
ENSG00000165556	CDX2	3619.56322648428	-1.05153303674639	0.332722520097024	-3.16039033498471	0.00157557904679942	0.0455604941032831
ENSG00000165794	SLC39A2	47.7115709360776	-5.27821433609248	1.21563571509328	-4.34193753157989	0.0000141231692951752	0.00210031703375392
ENSG00000166130	IKBIP	276.220190392702	0.744679758047956	0.235913752800731	3.15657628776295	0.00159633123799842	0.0458422294829341
ENSG00000166165	CKB	8921.23640866134	-1.64666541554206	0.511154115181719	-3.2214656336214	0.00127536756735644	0.0400298785211473
ENSG00000166527	CLEC4D	4.6325942410957	1.91492160365395	0.570252421895291	3.35802449955323	0.000785016550156491	0.0280183621272997
ENSG00000166546	BEAN1	29.6193371192074	1.93471336820753	0.548560082925195	3.52689418794506	0.000420464761139913	0.0188597694655648
ENSG00000166619	BLCAP	1850.63034571975	-0.617194027331472	0.138400025179229	-4.45949360581548	8.21535240966461E-06	0.00157886434310047
ENSG00000166927	MS4A7	119.405289425568	1.12199696549024	0.319396231262918	3.51286851774605	0.000443296785943051	0.0194303980701775
ENSG00000167077	MEI1	40.297785186577	1.13134863583271	0.351005920177868	3.22316112292185	0.00126784216206611	0.0398437340591946
ENSG00000167080	B4GALNT2	187.384976796232	-1.56454649253913	0.401212856738105	-3.89954226066551	0.0000963747131537635	0.00682518002493358
ENSG00000167083	GNGT2	13.3542497280529	1.30470339385839	0.322062918527914	4.05108231590873	0.0000509812670012132	0.00463169445366658
ENSG00000167220	HDHD2	508.062328297478	0.823579952934442	0.221177353113742	3.7236179081631	0.000196388078286192	0.0112415909599754
ENSG00000167286	CD3D	99.5317520188386	1.32015327988007	0.407507014004111	3.23958418999569	0.00119704122158562	0.0380979336052166
ENSG00000167434	CA4	169.150377566078	-3.31247209852224	0.832813191498427	-3.97744912344907	0.0000696585206388348	0.00554251108165812
ENSG00000167600	CYP2S1	2421.9382672646	-1.25915320553216	0.24242033478084	-5.19409069651892	2.05722837840257E-07	0.000151169981782382
ENSG00000167613	LAIR1	177.514408208231	1.34737512166703	0.306703294675574	4.39308982021946	0.0000111750929257207	0.00186413292008799
ENSG00000167619	TMEM145	17.518526194487	1.43446131921582	0.442970085484286	3.23828034041523	0.00120252582393769	0.0381752289520448
ENSG00000167634	NLRP7	1.77206863447644	2.87704655497512	0.894969189670957	3.21468782185998	0.00130586453394959	0.0408843602959855
ENSG00000167779	IGFBP6	227.16136257719	1.49289675159987	0.396253725814157	3.76752735518767	0.000164872479152527	0.00999799519210373
ENSG00000167851	CD300A	108.94205697704	0.993107501666444	0.31584058609846	3.14433149309334	0.00166466751369354	0.0470475714503612
ENSG00000167895	TMC8	329.302244601287	1.19683645483278	0.297018711322359	4.02949851039463	0.0000558959700279853	0.0047900244472215
ENSG00000167964	RAB26	177.637007365407	1.75755051264642	0.486430405795718	3.6131592345083	0.000302488810934952	0.0149355344909068
ENSG00000167984	NLRC3	79.6684296651852	1.05679004868037	0.274359205631073	3.85184833236986	0.000117229625215256	0.00791585123345391
ENSG00000168267	PTF1A	10.8187971917854	-4.66852258165297	1.36680091370932	-3.41565661452712	0.000636284283462834	0.0242701168519625
ENSG00000168394	TAP1	4531.73648928429	0.830611967025	0.25946746638559	3.20121816656059	0.00136847875054129	0.0421641735901594
ENSG00000168421	RHOH	66.7244764309614	1.17152660011154	0.342496241338137	3.42055315858174	0.000624939208194737	0.0240254594200237
ENSG00000168612	ZSWIM1	291.564335617833	-0.568228240483013	0.179883234323525	-3.15887271329044	0.00158380647552559	0.0456399319314087
ENSG00000168899	VAMP5	336.059390471683	1.33740014144465	0.324628274771162	4.11978944960173	0.0000379218769O9954	0.00398563633340949
ENSG00000168903	BTNL3	212.763840099061	-1.60659239629363	0.46915330447724	-3.424450773258	0.000616043440720786	0.0238994244145468
ENSG00000168995	SIGLEC7	14.2983410963492	1.45821344572219	0.388625894598227	3.75222924151706	0.0001752690824203	0.0103765942066085
ENSG00000169035	KLK7	289.273351210798	2.92322954206772	0.730278172171598	4.00289869458241	0.0000625710995050018	0.00515228391044056
ENSG00000169136	ATF5	694.758782377984	0.851837504328373	0.217385089706312	3.91856454129034	0.0000890778785454 13	0.00648719576467403
ENSG00000169245	CXCL10	580.156443965999	1.91425335232618	0.489475106400897	3.91082881906223	0.0000919799461372287	0.00659159778520 98
ENSG00000169248	CXCL11	512.787518716046	2.04685464127835	0.536668863253004	3.81399924875721	0.000136735883244421	0.00887327092721715
ENSG00000169429	CXCL8	4603.70841802203	2.3195918197052	0.529677915633303	4.37924963688888	0.0000119088650468675	0.00193381050183383
ENSG00000169442	CD52	223.250298852607	1.27635011390972	0.376430594234753	3.39066519421572	0.00069723220662948	0.0258835801640876
ENSG00000169469	SPRR1B	39.4916177097019	4.40309737791444	1.08933007158749	4.04202315970013	0.0000529919972244969	0.00467325979495102
ENSG00000169495	HTRA4	6.51361588839785	1.53967990720265	0.384131567202482	4.00820978711979	0.0000611807616411402	0.00508149103813216
ENSG00000169507	SLC38A11	60.9102417837185	2.01041033441854	0.585419242267934	3.43413777557113	0.000594441969905266	0.0234251390790428
ENSG00000170011	MYRIP	111.410696570012	-1.40143206558398	0.420857396503078	-3.32994519575642	0.000868630791052006	0.0302676034639377
ENSG00000170088	TMEM192	689.008821386985	-0.677287487093469	0.19515260553862	-3.47055313570711	0.000519387568709984	0.0214343506372852
ENSG00000170099	SERPINA6	38.2068626321532	-3.02802721745621	0.896461110803058	-3.37775635882707	0.00073079799021551	0.0265381642260818
ENSG00000170175	CHRNB1	159.67945884218	0.860624830037718	0.238279899966441	3.6118230289627	0.000304052038520716	0.0149831087384646
ENSG00000170191	NANP	213.480318501806	-0.682530184009606	0.20668790757494	-3.30222600837031	0.000959207483698387	0.0324726826188625
ENSG00000170298	LGALS9B	76.839918224967	-2.05054600037859	0.590549944421675	-3.47226516528874	0.000516086309127188	0.0213829193154787
ENSG00000170442	KRT86	25.0291009922316	2.06845424983429	0.464291270579109	4.45507901161766	8.38623040470573E-06	0.00158724870023612
ENSG00000170458	CD14	560.628193029882	1.07920022546683	0.290779538941372	3.71140359254927	0.000206113168841848	0.0115593569883648
ENSG00000170471	RALGAPB	1370.4530793565	-0.557392237924703	0.146527423460966	-3.80401309706498	0.000142370647692203	0.00912126828707127
ENSG00000170476	MZB1	265.624186369975	1.83133527018927	0.51945031558839	3.52552537794665	0.000422643680070467	0.0189235299334777
ENSG00000170542	SERPINB9	457.727210140721	1.06720398437152	0.227637870744084	4.68816537812065	2.75665210974838E-06	0.000800475337508591
ENSG00000170835	CEL	428.012115733056	-2.44198202147075	0.670207008109248	-3.64362352515521	0.000268826545780332	0.0137348924739792
ENSG00000170891	CYTL1	11.0491648934352	1.28686205096871	0.387930829765397	3.31724614861611	0.000909094856048527	0.0312418512840666
ENSG00000171115	GIMAP8	81.3919246344622	1.05360453152395	0.279816958491269	3.76533480030957	0.000166326063559261	0.0100617200289699
ENSG00000171310	CHST11	269.752927025374	1.26495456064723	0.392326928491439	3.22423588284187	0.00126309310699402	0.0397444813414843
ENSG00000171365	CLCN5	476.014993290244	-0.81993070470172	0.246935636472248	-3.32042274827299	0.000898812304613494	0.0309736918875359
ENSG00000171488	LRRC8C	125.361368153908	0.955786712086909	0.218403901877649	4.37623459961053	0.0000120747053428625	0.00193381050183383
ENSG00000171608	PIK3CD	162.820579337354	1.33712697123435	0.318981601967372	4.19182636130045	0.0000276673840933101	0.00324526724970544
ENSG00000171631	P2RY6	57.072180327913	1.26398192333628	0.331340117256036	3.81475667300366	0.000136317185969873	0.00886913691216485
ENSG00000171700	RGS19	170.674071623079	1.21860165356355	0.301042209418296	4.04794283139981	0.0000516697620345735	0.00463300480097051
ENSG00000171759	PAH	34.3204637141022	-2.47467716305429	0.723770730743626	-3.41914512142779	0.000628182138657351	0.0240713229328455
ENSG00000171860	C3AR1	90.1949669431594	1.17073204394462	0.333383143797123	3.51167137789382	0.000445298251649634	0.0194355464303021
ENSG00000171889	MIR31HG	6.09294468187241	2.43377156324142	0.779877622921204	3.12070957251625	0.00180415870852151	0.0494940662776087
ENSG00000171954	CYP4F22	2.20161256125739	1.85093552452305	0.540939146155655	3.42170748350765	0.000622292242727378	0.0240254594200237
ENSG00000172016	REG3A	1939.09592129335	4.87469611111457	1.0592125650215	4.60218871272136	4.18074133341782E-06	0.00106421277563708
ENSG00000172175	MALT1	538.395279170251	0.599588554918298	0.164829220499351	3.63763508133959	0.000275152892653095	0.0139440565315313
ENSG00000172243	CLEC7A	107.164532421936	1.35351350538439	0.362273265072973	3.73616724135509	0.000186846424010111	0.0108562117615549
ENSG00000172315	TP53RK	683.888203722326	-0.803050957703782	0.229147486561019	-3.50451567135099	0.000457438644154153	0.0197461965251802
ENSG00000172322	CLEC12A	21.8147356074004	1.84404128305675	0.462099864093165	3.99056876304333	0.0000659150346165989	0.00534682215863996
ENSG00000172349	IL16	112.616385242149	1.14511425459181	0.332824879524583	3.44059090843064	0.000580445409555859	0.0231085235678311
ENSG00000172456	FGGY	500.460266301037	-1.74131905890955	0.439275570647438	-3.96406988065158	0.0000736826678344424	0.00568175238634478
ENSG00000172543	CTSW	52.2523286191892	1.52657991048254	0.447221401391814	3.413476872376	0.000641396022181051	0.0243590827217931
ENSG00000172575	RASGRP1	58.6622592562472	1.09178000275911	0.340313404211071	3.20816046636892	0.00133586950057471	0.041615166586482
ENSG00000172840	PDP2	372.864288497623	-0.552110678902613	0.177140018536388	-3.11680377739826	0.00182823215079261	0.0497024505499483
ENSG00000172889	EGFL7	427.601453756462	1.32529226535593	0.301248050018146	4.39933890120152	0.0000108581147001927	0.00184543631067765
ENSG00000172943	PHF8	583.358841315682	-0.391153873901319	0.125573469336132	-3.1149404087442	0.00183982074886625	0.0498547522264581
ENSG00000173369	C1QB	619.798034542285	1.40497803355679	0.338664897012569	4.14857886350307	0.0000334545600196387	0.00366591547162568
ENSG00000173372	C1QA	974.135866256296	1.55942711317547	0.34006465074084	4.58567836962242	4.52514757093989E-06	0.00109414212713492
ENSG00000173406	DAB1	26.1040956283835	-1.80115421719855	0.552453649661116	-3.26028114449675	0.00111301827138643	0.0359271944345201
ENSG00000173432	SAA1	487.570862863499	2.69204279713947	0.607953795405143	4.42803847510398	9.50939412381714E-06	0.0017469316381577
ENSG00000173762	CD7	173.565349541465	1.46044030254267	0.364271113968296	4.00921249734278	0.0000609215761771389	0.00508149103813216
ENSG00000174015	SPERT	10.8512891499117	-1.77448015152999	0.542175711188906	-3.27288758037285	0.00106454809300424	0.0348580203874416
ENSG00000174564	IL20RB	25.7724411071868	1.28843240000271	0.333147520786034	3.86745306392422	0.000109977987480111	0.00761132974848503
ENSG00000174600	CMKLR1	85.0679485909391	1.57895169663385	0.312994931686283	5.04465579722691	4.54338504451984E-07	0.000246765069461487
ENSG00000174837	ADGRE1	6.66067060112903	1.5368903955324	0.448235606427556	3.42875571126854	0.000606355033205696	0.0237076277771692
ENSG00000174992	ZG16	431.92575261703	-3.01538605629331	0.738286588712852	-4.08430290133052	0.0000442093324592506	0.00425360577394859
ENSG00000175040	CHST2	102.522358091161	0.981919378661421	0.275938793204625	3.55846804741684	0.000373024159455456	0.017207531024209
ENSG00000175463	TBC1D10C	134.64428263278	1.52520621954737	0.384575617426624	3.9659462286071	0.0000731053434852O7	0.00565468700196412

ENSG00000175489	LRRC25	57.9347750291937	1.35023518344319	0.304720210820987	4.43106540194803	9.37686413003706E-06	0.00173534498833219
ENSG00000175779	C15orf53	21.7382263616008	3.68683518315638	0.583216187199842	6.32155839305103	2.58938491910843E-10	1.61732982047512E-06
ENSG00000175832	ETV4	2034.72970020104	-1.12114538350473	0.285883892098602	-3.92168084488666	0.0000879334146311894	0.00646155420925187
ENSG00000175874	CREG2	42.4512443431556	1.68125570577748	0.488404799926806	3.44234066911185	0.000576703481704106	0.0230533756590326
ENSG00000176049	JAKMIP2	13.0604135568369	1.1169311981471	0.350286688458718	3.1886201644192	0.00142953580937062	0.0428757775045804
ENSG00000176083	ZNF683	22.3259840599369	1.95728002098682	0.614803182934357	3.18358797630982	0.00145461902468127	0.0433160926253122
ENSG00000176532	PRR15	2264.58812443353	-0.913399328304535	0.268577770008192	-3.40087464527192	0.000671706224134256	0.0251955480076645
ENSG00000176563	CNTD1	71.6796869431487	-1.31573398585366	0.377016351108507	-3.48985921163665	0.000483275076803555	0.0205692410883476
ENSG00000176809	LRRC37A3	174.628729672016	-0.805752165550933	0.244571663264141	-3.29454424440295	0.000985814240531899	0.0330136987358669
ENSG00000176956	LY6H	13.4454651569805	1.7128485090064	0.529114922712999	3.23719561758699	0.00120710635652786	0.038237513052727
ENSG00000177182	CLVS1	6.98016023347428	2.01070791941767	0.456797067343982	4.40175312663194	0.0000107379696748157	0.00183751667366846
ENSG00000177359	AC024940.1	28.9468957026003	1.73639932837166	0.483047316913191	3.59467751413617	0.00032479363248605	0.0157260544845571
ENSG00000177494	ZBED2	27.1877124215968	2.44353971430345	0.55609185940305	4.39412962622132	0.0000111217441521565	0.00186413292008799
ENSG00000177675	CD163L1	107.35554779236	1.42502562167832	0.445691599635051	3.1973356079522	0.00138703446498356	0.0424574558316833
ENSG00000177728	TMEM94	1320.51759292487	-0.64541877268722	0.196558257788173	-3.28360039384749	0.00102490133179487	0.0338705487745542
ENSG00000178093	TSSK6	35.4040598725348	-0.709284244074409	0.226005628553946	-3.13834769785438	0.0016990320445087	0.0477572664603769
ENSG00000178171	AMER3	4.87328137853893	3.61217972956026	1.03814517050423	3.47945531336992	0.000502434154432769	0.021167325897171
ENSG00000178460	MCMDC2	33.7919002745838	-1.63951774378022	0.448390577195859	-3.65645003968062	0.000255732224898634	0.0133854642936103
ENSG00000178934	LGALS7B	15.5905869642934	2.04314464592308	0.572370288261027	3.56962038006996	0.000357498910334826	0.0167349680380091
ENSG00000179044	EXOC3L1	106.195933777153	1.13087868152184	0.25942944571793	4.35909917007423	0.0000130598924304934	0.00203810834464363
ENSG00000179144	GIMAP7	88.6769800807554	0.996973525760091	0.313479969643122	3.1803420387436	0.00147101312465632	0.0436624722910505
ENSG00000179344	HLA-DQB1	1366.83256958197	1.6031308424408	0.33264814640779	4.81929888908967	1.44063591106888E-06	0.000499900661140903
ENSG00000179583	CIITA	553.181924906398	1.52013676856022	0.418381297079204	3.63337648975365	0.000279736381621598	0.0140905922549073
ENSG00000179673	RPRML	14.8997156676082	-3.78805473027906	1.18284541238599	-3.20249348783282	0.00136243380600231	0.0420823418223686
ENSG00000180061	TMEM150B	196.784343131735	-1.42074324969754	0.324649321136092	-4.37623970604876	0.0000120744226111969	0.00193381050183383
ENSG00000180096	01-sep	180.407580400536	1.2353101397638	0.321245142991405	3.84538153094145	0.000120365007675608	0.00805450767331783
ENSG00000180353	HCLS1	333.420879416339	1.18337127781472	0.292552994060593	4.04498091572982	0.0000523273866154762	0.00463598378440091
ENSG00000180720	CHRM4	3.69201705979607	1.67065523907196	0.514831317215797	3.24505363835837	0.00117428502293834	0.0374350363080741
ENSG00000180785	OR51E1	58.4019656608571	1.39082256282267	0.421480927497278	3.29984697310331	0.000967375616660779	0.0327000997942811
ENSG00000181036	FCRL6	7.16213437848542	2.03484385355943	0.492076971070076	4.13521455623992	0.0000354623156299141	0.00378628416110159
ENSG00000181264	TMEM136	42.8471984605719	0.874950421039007	0.269431057097836	3.24740002308381	0.00116464573225889	0.0373044986855848
ENSG00000181631	P2RY13	24.2668830829594	1.55321889800261	0.388900580273433	3.99387138201376	0.0000650031338081671	0.00529002701974999
ENSG00000181778	TMEM252	15.9300366636992	-3.25776760309958	0.791546607074694	-4.11569903020526	0.0000386007736387653	0.00400166692361374
ENSG00000182022	CHST15	230.286111401349	1.36781134435659	0.320170540310445	4.27213366673375	0.0000193611456849621	0.00257297267975049
ENSG00000182162	P2RY8	53.4700569783239	1.12944771561597	0.309286617043151	3.65178153136315	0.000260427391794061	0.0134989899909229
ENSG00000182247	UBE2E2	130.497270995156	1.22137716101686	0.370839398453757	3.29354746585579	0.00098931636100116	0.0330136987358669
ENSG00000182287	AP1S2	413.662077755245	0.811281299921755	0.242386173200842	3.34706096972589	0.00081673244939822	0.0290672984555059
ENSG00000182487	NCF1B	31.5364232102644	1.48789254876483	0.41454546744768	3.5892143699596	0.000331676018407245	0.0159357570074742
ENSG00000182566	CLEC4G	2.72897285814741	2.69883607923828	0.776201151273601	3.47698025802976	0.000507095154501354	0.0212215499833531
ENSG00000182578	CSF1R	241.349787571201	1.32920852317883	0.305426253094149	4.35197861910416	0.0000134914421568127	0.00205726877171705
ENSG00000182782	HCAR2	78.6322144929095	3.47175213908496	0.56904382676529	6.10102768150569	1.05388652773798E-09	3.29128762612572E-06
ENSG00000182985	CADM1	114.806813549283	1.32790507422099	0.392473815687691	3.38342335499309	0.000715881860017691	0.0263023417510029
ENSG00000183044	ABAT	349.97303543845	-1.1479563027664	0.36295841646056	-3.16277636970333	0.00156272325687213	0.0454284001785879
ENSG00000183307	TMEM121B	11.1224305663619	1.84285756123967	0.328023981079326	5.61805742121646	1.93116326155677E-08	0.000026804546070408
ENSG00000183484	GPR132	46.828154566648	1.10957455407711	0.300229895373935	3.69574972770495	0.00021923886123838	0.0121182825424329
ENSG00000183696	UPP1	513.53255860019	0.786329092807591	0.21471486988686	3.66220138000658	0.000250057162230835	0.0131802281459392
ENSG00000183760	ACP7	3.79124699027545	5.10331451272627	1.14081154872324	4.473407127091	7.69830026588259E-06	0.00153867467074249
ENSG00000183779	ZNF703	5110.48109722058	-0.755759370724218	0.236574728469644	-3.19459046032972	0.00140029393232897	0.0424574558316833
ENSG00000183844	FAM3B	330.106356006641	-2.86264212112127	0.666017960165149	-4.29814553411057	0.0000172233066472841	0.00241745558020082
ENSG00000183856	IQGAP3	1013.04230104995	-0.77379182146943	0.216351694127028	-3.57654616291159	0.000348163856887267	0.0164793242025849
ENSG00000183918	SH2D1A	32.4440426934373	1.53324728342251	0.425470274134648	3.60365312604022	0.000313775749680895	0.0153412393934002
ENSG00000184060	ADAP2	97.2075666480245	1.01690128997764	0.284075031974191	3.57969260061555	0.000343998630764719	0.0163704034114776
ENSG00000184182	UBE2F	722.754915408343	0.452996150099975	0.12164734252127	3.72384748167245	0.000196209487934809	0.0112415909599754
ENSG00000184371	CSF1	353.52808113435	1.09440183205273	0.255060405006008	4.29075548604633	0.0000178066293432445	0.00245973610713429
ENSG00000184557	SOCS3	1396.47199636765	1.20021158227832	0.304609697202153	3.94016209366378	0.0000814265713868807	0.00615016760933985
ENSG00000184634	MED12	694.708742729189	-0.561744364649304	0.16980994750091	-3.30807689959561	0.00093939011003738	0.0320450642251265
ENSG00000184922	FMNL1	330.166559607598	1.10408461584325	0.302490059469347	3.64998644180282	0.000262254151153025	0.0135494479178965
ENSG00000185105	MYADML2	46.4721482303947	-2.08526212265752	0.559388702883827	-3.72775158294641	0.000193195667145517	0.0111473453763593
ENSG00000185176	AQP12B	33.408814155563	-1.6426870800573	0.520877943500271	-3.15368907544545	0.001612207536836	0.0461252899488063
ENSG00000185215	TNFAIP2	1196.67337248368	1.19478347594904	0.337639084494887	3.53864090626963	0.00040219256496693	0.018170667347439
ENSG00000185291	IL3RA	124.943854911853	0.985629801518144	0.293818560243385	3.35455225545918	0.000794934517705474	0.0283318744551289
ENSG00000185338	SOCS1	254.142417015063	0.952941913173212	0.273287685774688	3.48695518596789	0.000488553214860035	0.0207585263946651
ENSG00000185386	MAPK11	125.183958085614	1.18579595212997	0.290460852068014	4.08246393167059	0.0000445607295153908	0.00426553741843879
ENSG00000185479	KRT6B	383.558910140679	2.57744288111664	0.687946558207069	3.74657428017954	0.000179265937476139	0.0105383063103362
ENSG00000185669	SNAI3	24.5913875569223	1.65533749453066	0.391912569242736	4.22374178437081	0.0000240279332070117	0.00298663623504467
ENSG00000185880	TRIM69	556.51725309034	0.764547600180966	0.240003050282176	3.18557451366585	0.00144466895377335	0.0430711326265791
ENSG00000186074	CD300LF	48.9900872724872	1.35445539013815	0.369459059448634	3.66605001420044	0.000246325770196666	0.0130932240415779
ENSG00000186198	SLC51B	121.488814871431	-3.3725357338176	0.57316178481525	-5.88409036182582	4.00249133146969E-09	9.99982434254387E-06
ENSG00000186469	GNG2	178.383177824371	0.910750277923369	0.229994821496362	3.95987297452162	0.0000749896405181885	0.00576474208832745
ENSG00000186517	ARHGAP30	200.493028166149	1.20217072853336	0.297189503576311	4.04513185717098	0.0000522936826563857	0.00463598378440091
ENSG00000186603	HPDL	366.708474120115	-1.44967005752378	0.363466153101475	-3.98845957224262	0.0000665037334241456	0.00535977185764146
ENSG00000186638	KIF24	131.444805150393	-0.645160331036917	0.176477339587725	-3.65576868137348	0.000256412491412675	0.0133854642936103
ENSG00000186818	LILRB4	184.171895641557	1.93410942802412	0.409205351415748	4.72650081757873	2.28421872786083E-06	0.000687577357793675
ENSG00000186827	TNFRSF4	100.305364629256	1.0858936485004	0.308606473230815	3.51870016572279	0.000433666529183388	0.0191765036550757
ENSG00000186891	TNFRSF18	83.4908621014247	1.48820020024424	0.317076280741813	4.69350844144676	2.68558903370586E-06	0.000789373604919808
ENSG00000187260	WDR86	27.7692740641207	1.01033511636418	0.251836780821177	4.01186480016833	0.0000602409986423381	0.00505054063785294
ENSG00000187474	FPR3	133.192136613944	1.33276014872808	0.313551811333808	4.25052607114179	0.0000213269008413909	0.00274655304443974
ENSG00000187555	USP7	2894.12136209226	-0.471644371666285	0.110359585087867	-4.27370555344844	0.0000192250883181154	0.00256855404566736
ENSG00000187688	TRPV2	168.362928577786	1.2232680628164	0.295073243936255	4.14564210057846	0.0000338862770191904	0.00368221921613172
ENSG00000187783	TMEM72	10.4672153922431	-2.93087860926213	0.654762776434517	-4.47624500772952	7.59673019214974E-06	0.00153383930916891
ENSG00000187957	DNER	18.2135462663654	3.27351864070488	0.644843613443492	5.0764535345619	3.84544781989405E-07	0.000223429461237751
ENSG00000188015	S100A3	101.49957542538	2.7254062854921	0.477987334004913	5.70183787644901	1.18522527676087E-08	0.0000211511916532811
ENSG00000188064	WNT7B	93.1387765389531	2.38062733954162	0.418911057831538	5.6828944832938	1.32433983364279E-08	0.0000218023935879327
ENSG00000188211	NCR3LG1	336.518149185816	-0.927544739798462	0.261066008566106	-3.55372945203114	0.000379809716727489	0.0174754437619145
ENSG00000188263	IL17REL	10.0625823146241	2.52008662598817	0.685025748721295	3.67882029353246	0.000234315293390404	0.012698770694286
ENSG00000188290	HES4	714.39338908411	0.898308545495873	0.287539968665141	3.12411714331795	0.00178339440712576	0.0491693173874959
ENSG00000188389	PDCD1	48.5117380309161	1.75990872083886	0.48802151688228	3.60621132461949	0.000310700142455246	0.0152505547330096
ENSG00000188536	HBA2	747.724418197453	3.05878974171264	0.467205880280719	6.54698468237337	5.87103826059134E-11	4.88940066342047E-07
ENSG00000188641	DPYD	135.955865223542	1.68491013408501	0.36457186897561	4.62161312341334	3.80767607495296E-06	0.0010046258862384
ENSG00000188820	CALHM6	63.1971495985424	2.04705782801391	0.38607521565124	5.30222543439077	1.14399447734632E-07	0.000102076992935788
ENSG00000188868	ZNF563	57.5305866351545	-0.811120622819233	0.233732970140545	-3.47028757787788	0.000519901396341453	0.0214343506372852
ENSG00000188883	KLRG2	10.7277900804805	-3.67130133809044	0.833878020278785	-4.40268390437134	0.0000106919890008851	0.00183751667366846
ENSG00000189350	TOGARAM2	52.8204884599350	1.6723673471206	0.350207835493269	4.77535673856373	1.79388961017554E-06	0.000574596641290072
ENSG00000196126	HLA-DRB1	4179.24430520027	1.38618266024632	0.377960885055666	3.6675296679588	0.000244905141801293	0.0130741668007767
ENSG00000196154	S100A4	1614.59538902661	1.10927003020102	0.325655240479671	3.40627108769116	0.000658567517668017	0.0247423321224327
ENSG00000196187	TMEM63A	2950.12446317821	-0.588659774000658	0.186837381104648	-3.15063310014675	0.00162905851654798	0.0463030693713705
ENSG00000196209	SIRPB2	20.5131432743228	1.31178091625621	0.383379981827909	3.42162078990613	0.000622490675681082	0.0240254594200237
ENSG00000196227	FAM217B	359.533925770545	-0.798952477741992	0.235770160868217	-3.38869208384925	0.000702268226963381	0.0259932879740046

ENSG00000196329	GIMAP5	135.811544221358	1.24791665997777	0.313532755586777	3.98017954341737	0.0000688632380618559	0.00549673846561472
ENSG00000196664	TLR7	27.1510065041052	1.2075948159506	0.298401618139156	4.04687757218345	0.0000519053709817056	0.00463300480097051
ENSG00000196735	HLA-DQA1	728.232238112254	1.81373609209229	0.36144412379033	5.01802622571142	5.22050519990585E-07	0.00026618184064173
ENSG00000196743	GM2A	1087.45574380328	0.605560455935045	0.179175183459501	3.37971165561508	0.000725619125675682	0.0265080437597578
ENSG00000196954	CASP4	681.449285588644	0.710879836132827	0.195452749761243	3.63709304167482	0.000275732351473148	0.0139451357676217
ENSG00000197044	ZNF441	98.9725346533437	-0.650859592805497	0.191792836499819	-3.39355527914157	0.000689916325065758	0.0256637191645867
ENSG00000197057	DTHD1	4.33870403242815	1.73408600169791	0.510822409565056	3.39469445589597	0.000687052298447065	0.0256581683473863
ENSG00000197122	SRC	2334.39303771701	-0.484154155926087	0.145114760362932	-3.33635361913025	0.00084885117298657	0.0299543752908142
ENSG00000197183	NOL4L	1222.30693938419	-0.623376219661287	0.190710671841459	-3.26870129312695	0.00108042295936467	0.0352139972002989
ENSG00000197191	CYSRT1	48.3872196577523	1.35531593029356	0.434736474211615	3.11755744155443	0.00182356406902837	0.0497024505499483
ENSG00000197273	GUCA2A	248.398450876288	-1.94474310744389	0.613721942501226	-3.16876906749999	0.00153085977236758	0.0448382187020299
ENSG00000197296	FITM2	522.005779309078	-0.901527863905569	0.22271427894849	-4.0479122764916	0.0000516765058814822	0.00463300480097051
ENSG00000197415	VEPH1	24.2059013111383	1.72474709006447	0.519639913336451	3.31911973233616	0.000903017015852562	0.0310757260661989
ENSG00000197467	COL13A1	119.534290748306	1.37768865128836	0.332410679088496	4.14453788026944	0.0000340499669538994	0.00368221921613172
ENSG00000197471	SPN	128.556681265941	1.07955063400586	0.313401215363874	3.44462810315669	0.000571845589604	0.0229694376377272
ENSG00000197506	SLC28A3	228.632883324342	1.75059682755235	0.459293490100184	3.81149932512759	0.000138126432854759	0.00889420308877137
ENSG00000197535	MYO5A	168.681754128125	0.872832367446549	0.250693241079649	3.48167490949321	0.000498288234452891	0.0210646924696633
ENSG00000197540	GZMM	26.0721879307574	1.60127248054593	0.418327187428541	3.82779921713662	0.000129294158374648	0.0085007506653479
ENSG00000197629	MPEG1	228.457255641818	1.26818845336133	0.315363630478719	4.02135291072161	0.0000578648360380920	0.00491733014821663
ENSG00000197646	PDCD1LG2	25.5429266542971	1.79202380307446	0.405320726783583	4.42124886455978	9.81320721128626E-06	0.00177674143500766
ENSG00000197860	SGTB	144.263137120916	0.821860769710554	0.202222260063596	4.06414590288968	0.0000482086642663522	0.00447749170271577
ENSG00000197872	FAM49A	60.8568714636291	0.957728031141982	0.29635542450671	3.23168719700724	0.00123061688205666	0.038874329643661
ENSG00000198010	DLGAP2	4.26016159993885	4.62326167008743	0.929695005530278	4.97287996879194	6.59655075198647E-07	0.000310958913184207
ENSG00000198019	FCGR1B	12.2489637573948	1.41086675138106	0.419396038067418	3.36404406174735	0.000768092794660877	0.0276115545061976
ENSG00000198216	CACNA1E	20.5081604933782	2.38532826186193	0.556414469976441	4.28696302948938	0.000018113242457922	0.00248649038224573
ENSG00000198821	CD247	79.3091495268358	1.43252817475423	0.312510696749049	4.58393325302581	4.56309952348417E-06	0.00109414212713492
ENSG00000198829	SUCNR1	22.4779338832099	1.49614988002609	0.361899346763161	4.13416021169338	0.0000356254899960954	0.00378752017898914
ENSG00000198844	ARHGEF15	98.5981887975632	1.00142872585482	0.31919349687431	3.13737195670109	0.00170469714248813	0.0478541049527229
ENSG00000198851	CD3E	162.481113940948	1.34820207830279	0.377044053690205	3.57571499963378	0.000349271993944572	0.0164793224025849
ENSG00000198879	SFMBT2	65.2498473566718	1.1248801059603	0.30833967530699	3.64818476519555	0.000264099688354267	0.0136046734306041
ENSG00000198959	TGM2	2748.46736092639	1.10754687125407	0.329461401587608	3.36168930841982	0.000774672343089188	0.0277681690383648
ENSG00000203747	FCGR3A	498.120698590573	1.63834635766305	0.402785236051587	4.06754322408485	0.0000475113976172831	0.00444578561074982
ENSG00000203814	HIST2H2BF	57.7431330310823	1.43971717206485	0.389712282822102	3.69430791772621	0.000220486490644938	0.0121603410204705
ENSG00000204136	GGTA1P	31.3752903377925	1.35364441709657	0.355848408115683	3.80399177353215	0.00014238291034093	0.00912126828707127
ENSG00000204160	ZDHHC18	537.720253406809	0.43128182558631	0.123449165766914	3.49359854242043	0.000476557067794425	0.0203928445450998
ENSG00000204161	TMEM273	70.6755580691706	1.41353842540046	0.34438995318818	4.10447056400648	0.0000405241863703043	0.0041202874359633
ENSG00000204252	HLA-DOA	272.465683699873	2.8052925778964	0.457748462861522	6.12845876173933	8.87344206375369E-10	3.16705823601175E-06
ENSG00000204257	HLA-DMA	779.34510978711	1.40667719668118	0.368145685948397	3.82097971094615	0.00013292258822449	0.00867778280311625
ENSG00000204287	HLA-DRA	5839.89430695246	1.63760240031923	0.390002101237169	4.19895789054555	0.0000268146175077073	0.00319017335148838
ENSG00000204397	CARD16	243.92871276576	0.967232021809549	0.24727576066179	3.91155210369558	0.0000917048662222675	0.0065915977852098
ENSG00000204472	AIF1	192.349336091701	1.55954800794937	0.310339184108072	5.02530163063867	5.02641888937403E-07	0.000261625103191918
ENSG00000204475	NCR3	4.33048249830007	1.4841784996063	0.437370824928387	3.39340992817551	0.000690282551977357	0.0256637191645867
ENSG00000204482	LST1	99.2110467521796	1.46172359210679	0.347581300111276	4.20541493929284	0.0000260603589871081	0.00314773102583305
ENSG00000204577	LILRB3	92.0001014111368	1.36734044939144	0.39300854246622	3.47916215971049	0.000502984125807258	0.021167235897171
ENSG00000204592	HLA-E	13500.298343276	0.63176727495097	0.197354780702463	3.20117542986424	0.00136868174758323	0.0421641735901594
ENSG00000204682	CASC10	15.0537818846888	1.18472837458614	0.354135759803999	3.34540735237198	0.00082161803317272	0.0291995802856149
ENSG00000204832	ST8SIA6-AS1	21.2187674874167	3.54197259534482	0.878300905507215	4.03275525863127	0.0000551266837375973	0.00476772190386881
ENSG00000205076	LGALS7	5.82312596875079	4.22326606383159	1.11860744062699	3.77546752367785	0.000159707832477825	0.00985219873240984
ENSG00000206172	HBA1	299.357451057837	2.95104248920861	0.469867690514792	6.28058185055334	3.37308238996754E-10	1.68546180861898E-06
ENSG00000206418	RAB12	379.655427164568	0.447542585918445	0.143414722080897	3.12061815847606	0.00180471879518498	0.0494940662776087
ENSG00000211644	IGLV1-51	320.332379802013	2.55023898924534	0.667268350310817	3.82190911356342	0.00013242249839028	0.00867778280311625
ENSG00000211651	IGLV1-44	353.13116300972	2.33520008619152	0.653125421259119	3.5754236631758	0.000349661194492614	0.0164793224025849
ENSG00000211662	IGLV3-21	786.391706730033	3.85881195617179	0.695384163736678	5.54918008980287	2.8701241513784E-08	0.0000358538590899019
ENSG00000211669	IGLV3-10	129.712946446768	3.17409535265395	0.827496323275687	3.83578181965707	0.000125165487823718	0.0082893354857424
ENSG00000211673	IGLV3-1	541.268028008691	3.54693691077272	0.691772915594041	5.12731393614461	2.93905205943248E-07	0.000198457504467192
ENSG00000211677	IGLC2	5018.66771756167	2.28795183117601	0.558003019883819	4.10024990841874	0.0000412704221950988	0.00415766221017076
ENSG00000211694	TRGV10	1.88760497402989	2.0138453439517	0.548849306102763	3.66921360129223	0.000243297725949974	0.0130658812996552
ENSG00000211745	TRBV4-2	1.71933789791379	1.91990081217755	0.558912683142626	3.43506395557608	0.000592413998296735	0.0233821032123943
ENSG00000211746	TRBV19	3.29069296384783	1.90256448883875	0.508269154731715	3.74322240711813	0.000181675288757963	0.0106548718646219
ENSG00000211751	TRBC1	110.409118070192	1.31709608776057	0.3985499710929	3.30472006847434	0.000950713047500671	0.03233490468913
ENSG00000211829	TRDC	15.7060837638256	1.85973453380217	0.533841209051419	3.48368485285489	0.0004945614612518	0.0209425822846017
ENSG00000211893	IGHG2	5351.98469596965	2.5698953690514	0.60188492243441	4.26974538364757	0.0000195696241650122	0.00257619780418335
ENSG00000211896	IGHG1	22940.1721929944	2.53538200646478	0.597151327546658	4.24579480863112	0.0000217819901242891	0.00279077559623199
ENSG00000211897	IGHG3	4333.44868206423	2.67162872702321	0.572369294359402	4.66766605642832	3.04640512344176E-06	0.000855184107910886
ENSG00000211934	IGHV1-2	241.835976161676	2.11081138411234	0.674949448720226	3.12736218707143	0.00176382490421471	0.0490351685946927
ENSG00000211943	IGHV3-15	232.968684979014	2.6450857574272	0.674646754966522	3.92069736933435	0.0000882930876287371	0.00646895748186618
ENSG00000211945	IGHV1-18	195.045231642715	2.23389266994312	0.670606568142103	3.33115238661032	0.000864872430022004	0.0302676034639377
ENSG00000211946	IGHV3-20	49.6297631505664	3.37958575209316	0.72745778657005	4.64574826812679	3.38845992259628E-06	0.000920187855501581
ENSG00000211947	IGHV3-21	297.09950547219	2.99574134265177	0.660475780691106	4.535732316357	5.74039505636703E-06	0.00125805289551118
ENSG00000211949	IGHV3-23	761.974410947307	2.02107525649501	0.648830166929561	3.11495266328211	0.00183974431588649	0.0498547522664581
ENSG00000211950	IGHV1-24	41.2301853830438	2.33038843536458	0.656848332569181	3.54783337311607	0.000388413860831489	0.0177731353461794
ENSG00000211962	IGHV1-46	117.360882001033	2.82308369983492	0.693284063872786	4.07204470280878	0.0000466022323943773	0.00437974021920927
ENSG00000211965	IGHV3-49	90.5399522054009	3.43997163150063	0.713680519331435	4.82004417708241	1.43526435139447E-06	0.000499900661140903
ENSG00000211970	IGHV4-61	167.461594376983	2.1673324838721	0.645210765409669	3.35910775217146	0.000781945715447867	0.0279486863444199
ENSG00000211976	IGHV3-73	31.8103905178396	2.1627882096061	0.602888035187196	3.58737955204992	0.000334017943538568	0.0160174746667324
ENSG00000211979	IGHV7-81	1.62915313447894	-2.46477616331774	0.740179567025009	-3.32997055461065	0.000868551685572155	0.0302676034639377
ENSG00000213203	GIMAP1	74.6511780396175	0.965852779033064	0.272471066966958	3.54479024060999	0.000392925642355562	0.0179467170906998
ENSG00000213402	PTPRCAP	222.152947177842	1.64947549381286	0.40352846730738	4.0876310531926	0.0000435800498010237	0.00422498284918415
ENSG00000213654	GPSM3	277.874411203212	1.01229635701864	0.259809910394644	3.89629612614861	0.0000976749393294635	0.00687076781919746
ENSG00000213742	ZNF337-AS1	70.5924992324598	-0.897458916645644	0.288233717807464	-3.11365000414398	0.00184788550139255	0.0499649040766142
ENSG00000213809	KLRK1	54.9611439545626	1.44432790915854	0.440012370619106	3.28247114308704	0.00102901517174323	0.0339615786668863
ENSG00000213886	UBD	2377.07337390338	1.63509703385602	0.511745217391318	3.19513886654598	0.00139763574366675	0.0424574558316833
ENSG00000214039	LINC02418	194.359651114632	-1.82372162271062	0.540671110609062	-3.3730702212963	0.000743349923318364	0.0269157311365014
ENSG00000214078	CPNE1	3105.1089537191	-0.886592028963362	0.223015504499557	-3.97547260650267	0.0000702396290239538	0.0055533762390331
ENSG00000214212	C19orf38	21.0738725396298	1.10508105759704	0.338456187957679	3.26506383075857	0.00109439411243614	0.035463479254351
ENSG00000214357	NEURL1B	838.637129771947	-0.753221121664416	0.217885405598517	-3.45695995376729	0.000546306026984799	0.0221933492328264
ENSG00000215458	AATBC	36.741583202896	1.45848404340515	0.411618969826037	3.54328675381882	0.0003951727581355	0.017956308943826
ENSG00000215808	LINC01139	26.0526852974745	2.31410852026148	0.684977697978263	3.37837060548344	0.000729167388765902	0.0265381642260818
ENSG00000215861	AC245297.1	9.36578390691746	1.75540696442506	0.463054593051001	3.79092873878852	0.000150085014925062	0.0094214171178084
ENSG00000216490	IFI30	2059.95772151546	1.31127254079897	0.259761034517035	5.04799552880198	4.46469593573 82E-07	0.000246765069461487
ENSG00000216588	IGSF23	13.3479933895561	-2.60297314578927	0.682730611974766	-3.81259181899036	0.00013751711675096	0.00887784921164335
ENSG00000220842	RPL21P16	14.1340722826736	-22.7256311608651	3.40810117295778	-6.66812104675352	2.59099052641767E-11	3.23666536560095E-07
ENSG00000222001	AC106876.1	102.350301423495	-1.08066006857675	0.276649807584624	-3.90623828012673	0.0000937440743536166	0.00669171986757359
ENSG00000222041	CYTOR	362.450038296764	0.719981211302698	0.218659600090161	3.29270341208813	0.000992290895511113	0.0330136987358669
ENSG00000223813	AC007255.1	48.3324066616293	-0.949841657591071	0.264415694762794	-3.59222873832497	0.000327861867396141	0.0158133221911683
ENSG00000223865	HLA-DPB1	1638.30996070374	1.75684262336122	0.361747882668655	4.85653878718183	1.19455390862007E-06	0.000464420001763836
ENSG00000223891	OSER1-DT	125.717823395261	-0.996055580871391	0.279471426129181	-3.56406948168999	0.000365149229596662	0.0169946638690228
ENSG00000224041	IGKV3D-15	24.8806115799813	2.82819339880486	0.840059850583113	3.36665702668889	0.000760852511685991	0.0274302152264975

ENSG00000224557	HLA-DPB2	3.40403502334082	1.82372854903586	0.583868366579506	3.12352689994127	0.0017869752983608	0.0491693731874959
ENSG00000224650	IGHV3-74	133.052907795603	2.78787166230686	0.66803104462119	4.17326662399011	0.0000300263210386195	0.00344118167352692
ENSG00000225217	HSPA7	66.2049719154138	1.92755365512114	0.476946331586355	4.04144770064584	0.0000531222229497522	0.00467325979495102
ENSG00000225492	GBP1P1	16.0976509627751	1.65108978330267	0.449736487708641	3.67123822155236	0.000241378226515029	0.0130532329050624
ENSG00000225783	MIAT	226.630141156525	1.76665461306831	0.444872867481875	3.97114488700592	0.0000715280597618179	0.00559177930742689
ENSG00000226025	LGALS17A	22.4132172625709	3.05105467540938	0.802737562543432	3.80081214306488	0.000144222613540796	0.00921549303504665
ENSG00000226147	TUBBP10	2.20830260082238	-3.71318755413541	1.18288742954666	-3.13908784672645	0.00169474633736222	0.0477572664603769
ENSG00000226789	AC110926.1	38.7984202280457	-1.14411169466323	0.332289566907584	-3.44311651223595	0.000575051510597336	0.0230241777896857
ENSG00000226812	AL117382.1	134.123576493577	-1.49151549740517	0.411872217993325	-3.6213063961244	0.000293119117987181	0.0145015604827559
ENSG00000226979	LTA	6.81852399713833	1.6481817236843	0.411899444212427	4.00141769269886	0.0000629640974101284	0.00515768855637589
ENSG00000227039	ITGB2-AS1	38.7942605431083	1.82506480170048	0.409426661474908	4.45761102886147	8.28781128530678E-06	0.00158063112329851
ENSG00000227507	LTB	178.121593317161	1.31854426991267	0.359374533318256	3.66899751559464	0.000243503445299903	0.0130658812996552
ENSG00000228495	LINC01013	2.8449389385656	1.90332739606767	0.519314255003313	3.66507827915397	0.000247262942674951	0.0130932240415779
ENSG00000229671	LINC01150	1.15970627380413	2.08768754434906	0.657570324583245	3.1748506073053	0.00149913661318324	0.0441159353872439
ENSG00000230006	ANKRD36BP2	41.2201798762455	1.4646446673961	0.415560340269417	3.52450540984382	0.000424274151205714	0.0189286882030778
ENSG00000230873	STMND1	9.44164243298128	-2.14904656048289	0.632529763227252	-3.39754219551372	0.000679941011678289	0.0254306081373808
ENSG00000231233	CFAP58-DT	2.99347863317207	2.04880525481622	0.499878394055444	4.09860733966626	0.0000415643472410968	0.0041704564316127
ENSG00000231389	HLA-DPA1	2339.25175611412	1.74907124193429	0.401068248152146	4.36103144537828	0.0000129450758978712	0.00203408664297116
ENSG00000231473	RB1-DT	4.02131339803876	-3.12429559593586	0.786189868728626	-3.97397081825573	0.0000706842271618361	0.00557089820634483
ENSG00000231475	IGHV4-31	84.0305473892919	3.30874954591563	0.666653867306952	4.9632196079231	6.93341096128934E-07	0.000314953344467005
ENSG00000231764	DLX6-AS1	34.0183397167356	-3.060225723324	0.918102167011085	-3.33320825642605	0.000858506553803789	0.0302038463157562
ENSG00000231999	LRRC8C-DT	3.41512920472441	1.30361962704514	0.398793694310026	3.26890732136727	0.00107963658230879	0.0352139972002989
ENSG00000232070	TMEM253	81.2132884064466	-1.32057936526402	0.422456246260245	-3.12595535503221	0.00177228455175342	0.0490351685946927
ENSG00000232629	HLA-DQB2	136.003107946498	1.69213108839035	0.502219321441463	3.36930702612878	0.000753574374733338	0.0272222827047922
ENSG00000232810	TNF	18.1425375637783	1.53072696438189	0.376573430728972	4.0648830731863	0.0000480565481429627	0.00447749170271577
ENSG00000233532	LINC00460	65.3212796095473	2.20069030369955	0.645076749578957	3.41151700962087	0.000646024705242965	0.0244179746381093
ENSG00000234336	JAZF1-AS1	1.50727269097948	3.15352658374454	0.729664291931158	4.32188695351707	0.0000154700450285812	0.00220859202853756
ENSG00000234663	LINC01934	32.7827620013525	1.7630596353324	0.405325033270434	4.34974277583315	0.0000136297313361311	0.00206378913758727
ENSG00000234883	MIR155HG	41.0189593276851	1.34612675430895	0.342679499051147	3.92823836277416	0.0000855703769344843	0.00630646105407421
ENSG00000235027	AC068580.3	31.5083209781536	0.924818474921192	0.259567234657003	3.56292455842226	0.000366746101537944	0.0169996003725862
ENSG00000235049	LINC00940	2.14855726999094	-3.66435810632959	0.896147690650842	-4.08901138122478	0.0000433215599477874	0.00422498284918415
ENSG00000235296	AC145207.1	1.26516829790422	-3.26592874264485	0.851038788609705	-3.83757918717221	0.000124253172652998	0.00825622677011305
ENSG00000235505	CASP17P	40.0274294050498	1.24719949986307	0.342521877486688	3.6412258072816	0.000271343011787895	0.0138351710336914
ENSG00000235568	NFAM1	102.318669835649	1.1898825595917	0.284332861993859	4.18482250432735	0.0000285389008139041	0.00331635301364921
ENSG00000235641	LINC00484	2.51685336893892	1.90817154988137	0.597152073791616	3.19545327501828	0.00139611386855153	0.0424574558316833
ENSG00000235831	BHLHE40-AS1	23.0870487438646	1.11328789348594	0.268702129688175	4.14320457667342	0.0000342486172143394	0.00368221921613172
ENSG00000236283	AC019197.1	7.6131657673256	2.53339826070384	0.797464321603951	3.17681705886024	0.00148900930252596	0.0440253354015487
ENSG00000236391	AC092573.2	2.97877730891151	-2.24412752893954	0.591231849125447	-3.79568105517161	0.000147238692841641	0.00933657741613086
ENSG00000236432	AC097662.1	11.4755582657599	-1.37057054298934	0.419739681256029	-3.26528704383642	0.00109353198297222	0.035463479254351
ENSG00000236481	LINC02195	1.67518420464011	2.09953805800601	0.610260103570125	3.4403986852874	0.000580857861054992	0.0231085235678311
ENSG00000236935	AP003774.4	66.0094551088634	-2.15763856368224	0.605494831277519	-3.56343019333442	0.000366040068214778	0.016998410900145
ENSG00000237181	AC147651.3	12.0945099489135	1.20873828561312	0.366027320795962	3.30231711388264	0.000958895957478097	0.0324726826188625
ENSG00000237517	DGCR5	13.5505987943749	1.40341636422361	0.384881468616657	3.64635992807807	0.000265981356284456	0.0136734119452898
ENSG00000237541	HLA-DQA2	339.147657334761	2.59043165037803	0.6160001169282	4.20524538744522	0.0000260799040324785	0.00314773102583305
ENSG00000237721	AF064858.3	5.4481014129346	-3.38271940236661	0.860960183185353	-3.92900794767457	0.0000852970207598683	0.00630491351084187
ENSG00000237976	AL391069.2	3.76522940447741	1.43417669534568	0.385682465075475	3.71854264897684	0.00020037546512563	0.0114036005027306
ENSG00000238266	LINC00707	6.063890221855	0.864499631052715	0.25014850468003	3.45594562781223	0.000548365923520919	0.022240867261764
ENSG00000238271	IFNWP19	3.42294982719805	2.53232123365449	0.806909806788133	3.1382952745788	0.00169933597035203	0.0477572664603769
ENSG00000239571	IGKV2D-30	1.22781468090279	3.6271228931463	1.13501433777774	3.19566262065718	0.0013951013913266	0.0424574558316833
ENSG00000239713	APOBEC3G	159.136739920212	1.53853882938478	0.268453185835075	5.731125241069	9.97665664815604E-09	0.0000191735992075023
ENSG00000239975	IGKV1D-33	21.4031326059289	2.49308121916816	0.72560604365051	3.43586060367623	0.000590674804890033	0.0233821032123943
ENSG00000240350	AC017002.3	7.91985238771532	1.47318091678062	0.397260448764599	3.70835033127994	0.000208613906210127	0.0116339505195398
ENSG00000240476	LINC00973	2.17287092191864	4.17721992649814	0.966268341172489	4.32304334987259	0.0000153891480661299	0.00220859202853756
ENSG00000240864	IGKV1-16	167.753614192218	3.41029550900081	0.786394353709912	4.33662257735234	0.0000144688753616564	0.00210168826764897
ENSG00000241127	YAE1	475.143563174702	-0.775890843035289	0.234376703303519	-3.31044353853935	0.000931482422191364	0.0318360558632408
ENSG00000241186	TDGF1	431.058810399312	-1.58559475087418	0.495869663223157	-3.19760386341807	0.00138574498106264	0.0424574558316833
ENSG00000241794	SPRR2A	18.1144954772952	4.5108685956982	0.993553322109719	4.54013739908774	5.62175814064614E-06	0.00124295579987525
ENSG00000241978	AKAP2	852.343425987231	0.858562013162529	0.27192786798891	3.15730866244847	0.00159232698037598	0.0458043893927425
ENSG00000242076	IGKV1-33	398.529277290252	2.28911426560028	0.676719428126919	3.38266373101816	0.00071786472078231	0.0263364642937228
ENSG00000242372	EIF6	4847.04327678908	-0.620730163388298	0.17615492060264	-3.52377419412828	0.000425446649471796	0.0189401392207324
ENSG00000242574	HLA-DMB	666.160166888422	2.28530422280307	0.402778510902394	5.67384843268582	1.39624678757174E-08	0.0000218023935879327
ENSG00000242600	MBL1P	2.55908547513854	2.13162367687961	0.673368840416669	3.16561080486082	0.00154757706569995	0.045248920567613
ENSG00000243064	ABCC13	22.4001774351102	-2.23871215978205	0.648437237946111	-3.45247315973563	0.000555472735465652	0.0224925945265379
ENSG00000243264	IGKV2D-29	17.5580989073617	2.36299633305739	0.724578039089766	3.26120335640568	0.00110940445635386	0.035856870553098
ENSG00000243290	IGKV1-12	229.545480907594	3.41164132597406	0.699456774208753	4.87755848791858	1.07407005528927E-06	0.000425945496211859
ENSG00000243709	LEFTY1	1434.88969979322	-2.61418682018582	0.658344056203572	-3.97085201203285	0.0000716160561210231	0.00559177930742689
ENSG00000243811	APOBEC3D	72.5553037986575	0.960915343707143	0.255528454054898	3.760502317682	0.000169572503611309	0.0101354053354664
ENSG00000244005	NFS1	662.572541133867	-0.786510019221232	0.150316981835446	-5.23234307672724	1.67374728697938E-07	0.000126717885508766
ENSG00000244437	IGKV3-15	593.999810690162	2.23925314923261	0.622020095536595	3.59996914135207	0.000318254947345271	0.0155298468837388
ENSG00000244682	FCGR2C	36.2085490846299	1.65621276918991	0.427267141903474	3.87629332274766	0.000106059817795403	0.00736055135500097
ENSG00000244734	HBB	629.548153244019	3.51217558523866	0.479962279532019	7.3176075183724	2.52430868590545E-13	6.30673282086617E-09
ENSG00000246627	CACNA1C-AS1	6.28116289546462	1.76402309636261	0.555411993646801	3.17606230427279	0.00149228884405664	0.0440358144498124
ENSG00000247134	AC090204.1	7.67243210727754	2.31568833084467	0.541843946105627	4.27371819411867	0.0000192239978830625	0.00256855404566736
ENSG00000247774	PCED1B-AS1	45.1357957149357	1.31784883750109	0.368863834684804	3.57272444078774	0.000353286463749143	0.0165911823502041
ENSG00000248323	LUCAT1	55.0630091138175	1.68627119292994	0.379489068781883	4.44353034553191	8.84946861766442E-06	0.00164996327591439
ENSG00000248409	AP000344.2	10.1579418781041	-2.15380238813333	0.63848824684287	-3.37328431460286	0.000742772131609957	0.0269157311365014
ENSG00000249138	SLED1	2.14352329774648	1.94104882068824	0.526401649563556	3.68739121979877	0.000226564895457048	0.0124406535123053
ENSG00000250378	AC114296.1	5.67301829807604	-2.34823276653171	0.73254787057505	-3.20556902948656	0.0013479571060335	0.0418872641009218
ENSG00000251003	ZFPM2-AS1	6.92663324252202	1.93285201263495	0.432875672446462	4.46514354403309	8.00150883059166E-06	0.00156179450487111
ENSG00000251442	LINC01094	18.23635645317	1.35336570990176	0.36182341012081	3.74040394304471	0.000183724750907667	0.0107247177025167
ENSG00000253755	IGHGP	83.5838926402497	3.27554044621413	0.655166184748773	4.99955663534459	5.74622923804922E-07	0.000281497629967494
ENSG00000253882	AC099548.2	62.085796299107	0.907180917685101	0.283241012768747	3.2028586143553	0.00136070766948335	0.0420823418223686
ENSG00000254777	AC022182.1	2.39510882613473	1.72744891748184	0.528852720200146	3.26640830518575	0.00108921076123205	0.0354333875763302
ENSG00000254838	GVINP1	44.2491113636752	1.18380119108233	0.369655273706747	3.20244637446039	0.00136265668816481	0.0420823418223686
ENSG00000255112	CHMP1B	1417.78303055228	0.481613976036869	0.153140569587445	3.14491435766707	0.00166135457052501	0.047007115050959
ENSG00000255177	MUC5B-AS1	15.424908241761	-8.60319913389838	2.60084587426334	-3.30784658138773	0.000940162987392639	0.0320450642251265
ENSG00000255221	CARD17	1.36756147855303	2.49062349623305	0.716590215230448	3.47565937030287	0.000509599103381928	0.0212906755834349
ENSG00000255398	HCAR3	77.2104895754852	3.35922515572659	0.610029019013639	5.50666452090779	3.65696200584688E-08	0.0000435073994067041
ENSG00000255833	TIFAB	12.6021446633454	1.77537507174572	0.548447677437143	3.23709105678398	0.00120754874313967	0.038237513052727
ENSG00000255986	MT1JP	6.9745507617921	2.70311367654212	0.777394852817723	3.47714377930918	0.000506785973481822	0.0212215499833531
ENSG00000256262	USP30-AS1	41.0381689003307	1.39478658449474	0.347457706100227	4.01426291605229	0.0000596318480953165	0.00502725989345869
ENSG00000256633	AP005019.1	1.17919053716056	2.12450824933572	0.640856150441008	3.31510940149943	0.000916072650906383	0.0313952799866188
ENSG00000256643	LINC02441	18.187526555584	-3.03084845547679	0.594029204745158	-5.10218762186456	3.35749643288663E-07	0.000200067637467126
ENSG00000258017	AC011603.2	74.7095929839225	-2.88159257537845	0.658224576293067	-4.37782586546184	0.0000119869059904196	0.00193381050183383
ENSG00000258227	CLEC5A	58.9131650973363	1.55949843715998	0.42791903326445	3.64437736097666	0.000268039906966585	0.0137348924739792
ENSG00000259347	AC087482.1	10.3064552379215	-1.67867553327995	0.434588385813595	-3.8626792341385	0.000112150225250102	0.00770269252078768
ENSG00000259768	AC004943.2	92.4792892148293	-0.738267119105127	0.213548894424622	-3.45717603031526	0.000545868150386995	0.0221933492328264
ENSG00000260032	NORAD	10562.4929186543	-0.71312357534168	0.177922511145572	-4.00805705107332	0.0000612203331123031	0.00508149103813216

ENSG00000260196	AC124798.1	250.692301153143	-0.789071235993622	0.247649158853005	-3.18624638035612	0.00144131796293575	0.043031911545288
ENSG00000260314	MRC1	200.835131308374	1.59199868342292	0.412207087980136	3.86213320887786	0.00011240124670894	0.00770269252078768
ENSG00000261269	AC093278.2	22.0509919119768	0.988707682129736	0.285989719266081	3.45714414024043	0.00054593275467761	0.0221933492328264
ENSG00000261308	FIGNL2	9.78289606558232	1.57556591979085	0.494065038790696	3.18898484225336	0.00142773363789492	0.0428731937610176
ENSG00000261329	AC016597.1	1.92474172251259	2.24177996117752	0.617946218837283	3.62779137219355	0.000285856095846139	0.0142836573972399
ENSG00000261465	AC099518.4	3.05158722835704	-1.44232150288766	0.456573548395011	-3.15901240437129	0.00158304752369744	0.0456399319314087
ENSG00000261469	AC020978.4	4.20397158908117	-1.96665828441113	0.616463947224376	-3.19022433228414	0.00142162394914045	0.0427957962569227
ENSG00000262406	MMP12	1311.22574975211	1.69120570831199	0.438260903798253	3.8589016123841	0.000113897776451705	0.00773266860562338
ENSG00000262943	ALOX12P2	39.7901590239929	2.10044978498805	0.621800460975798	3.378012588945	0.000730117381114357	0.0265381642260818
ENSG00000266053	NDUFV2-AS1	13.8420729042766	0.834929225485935	0.233547469454763	3.57498724963773	0.000350244964608253	0.0164793224025849
ENSG00000266835	GAPLINC	16.8450999644616	1.23444555057619	0.385178548096524	3.20486578672821	0.00135125472826137	0.0419375753178659
ENSG00000267120	AD000671.2	10.6640761402597	1.35983990556557	0.414882621010295	3.27764971753741	0.0010467520290252	0.0344555558195861
ENSG00000267607	AC011511.5	13.9314951198194	1.48656475391621	0.405061325610174	3.6699745444147	0.00024257462367749	0.0130658812996552
ENSG00000267934	AC010300.1	14.1072055306417	-1.43239502567198	0.458165227612438	-3.12637218921303	0.00176977413885593	0.0490351685946927
ENSG00000269220	LINC00528	5.60513155487551	1.41473174637105	0.425034850350395	3.32850763932596	0.000873126103941684	0.0303544309385232
ENSG00000269416	LINC01224	91.6011436403842	-1.03452991061962	0.298059180779542	-3.47088758653204	0.000518741113494624	0.0214343506372852
ENSG00000270550	IGHV3-30	407.04721435242	2.47539401226385	0.632619239261939	3.91292875498353	0.0000911834431803255	0.00658418249831576
ENSG00000270640	AC104695.3	4.29596655503811	1.71742510638652	0.489315598598287	3.50985153816131	0.00044835695449617	0.0194813046106649
ENSG00000271503	CCL5	509.803633167522	1.22796109186775	0.359110161593829	3.41945515108157	0.00062746675054599	0.0240713229328455
ENSG00000271605	MILR1	27.7694698154737	1.05766059183889	0.272713575513962	3.87828361622849	0.00010519603556454	0.00732094081488706
ENSG00000272449	AL139246.5	69.0736707194638	0.738156623582138	0.212173871157432	3.47901756024628	0.000503255608506227	0.021167235897171
ENSG00000272510	AL121992.3	6.39595574240399	-1.45631838569876	0.354937065951251	-4.10303269340369	0.0000407769601976811	0.00412458126955006
ENSG00000272769	AC097532.2	43.4701195288218	-2.22172029166604	0.50698643636074	-4.38220854114764	0.0000117482274008683	0.00193103758804798
ENSG00000272908	AC006033.2	2.6602002897837	2.13208329982448	0.507363033270946	4.20228349329876	0.0000264235928137643	0.00317015020957119
ENSG00000273443	AL645608.8	2.08991520122684	2.21999656648009	0.640626240605013	3.4653537831724	0.000529534383611867	0.0217239524468947
ENSG00000273820	USP27X	73.9070646824949	-0.70514956277512	0.191558767411179	-3.68111348963488	0.000232217606964718	0.0126124449834924
ENSG00000274576	IGHV2-70	43.1327694554664	2.77548845187319	0.811974807438955	3.41819527705219	0.000630378614617037	0.0241184981739541
ENSG00000274825	AL023803.2	10.2149759690662	-0.908043227645409	0.259338747863577	-3.50137893055256	0.000462857219604402	0.0199379737493041
ENSG00000275302	CCL4	140.510583999306	1.7290250415073	0.383287685543425	4.51103728797312	6.45113781203132E-06	0.00135441367307387
ENSG00000275385	CCL18	390.466559627423	1.96988273481726	0.58217046680814	3.38368716231436	0.000715194430121275	0.0263023417510029
ENSG00000276070	CCL4L2	78.9240706206915	1.59883796228684	0.437591050258897	3.65372637612424	0.00025846170380091	0.0134249630099001
ENSG00000276231	PIK3R6	18.8086744846605	1.36750851458541	0.361854962786407	3.77916197156762	0.000157357047646909	0.0097553560258322
ENSG00000276980	AC008760.2	8.07080963332903	2.33455420525374	0.708387647281743	3.2955885301106	0.000982157521725293	0.0330114926882151
ENSG00000277150	F8A3	102.36818931951	-1.91733715440128	0.590868103297308	-3.24494949668409	0.001174714555937	0.0374350363080741
ENSG00000277632	CCL3	159.650001327731	2.11769463318158	0.415082737941704	5.10186148352669	3.36328881428887E-07	0.000200067637467126
ENSG00000277734	TRAC	168.694727705695	1.12683980344359	0.360240683403484	3.12801928088028	0.0017598863609169	0.0490178381729629
ENSG00000278535	DHRS11	575.416046282742	-1.21979116125835	0.264227857095887	-4.61643664171147	3.90385132938307E-06	0.00101597730847194
ENSG00000279249	AC007614.1	10.0688438319346	-2.3911804783275	0.657296426126725	-3.63790275327997	0.000274867163386386	0.0139440565315313
ENSG00000279369	AC046185.3	18.4759790533997	1.02173422096767	0.327748719393416	3.11743161913386	0.00182434263163771	0.0497024505499483
ENSG00000279519	AC007382.1	31.4671470287117	-0.696328973791539	0.204773023990186	-3.40049172602399	0.000672647715382334	0.0251955480076645
ENSG00000279611	AC012313.10	1.36541982770582	3.15654398794707	0.736540592614243	4.28563478998949	0.0000182218125193057	0.00248696585590559
ENSG00000279901	AC092117.2	24.2620745493744	-1.44568414748247	0.366046908898806	-3.94945050029676	0.0000783308067401029	0.00598476108744566
ENSG00000280721	LINC01943	17.0704495288395	1.43111396839192	0.298512523720328	4.79415051186497	1.63365591099018E-06	0.000537042885265508
ENSG00000281103	TRG-AS1	21.4154667436523	1.50674546204955	0.412834105985419	3.64976013416578	0.000262485302283937	0.0135494479178965
ENSG00000284648	AC097493.4	12.6275097793888	-1.29014884743825	0.407645276565862	-3.16488113957038	0.0015514631565821	0.0452824246542606
ENSG00000285219	AL591485.1	1373.99260355719	-4.67138702778051	0.796282451473775	-5.86649500957181	4.45103657346837E-09	0.00000101095179774122

Supplementary Table 4. Differentially expressed genes in tumour tissues according to *F. nucleatum*

Gene ID	Gene symbol	Base mean	Log2 fold change	LfcSE	Stat	*P*-value	*P*-adjusted
ENSG00000002549	LAP3	2020.61451836466	0.568687468938137	0.18162404367912	3.13112436777837	0.00174138398301744	0.048560603752026
ENSG00000003393	ALS2	359.207709330485	0.396896569351534	0.122027378862758	3.25252064782868	0.00114386284885945	0.0367942437577118
ENSG00000004399	PLXND1	1484.85012195244	1.00037295143011	0.282579328242183	3.54014908894096	0.000399901052784313	0.0180998693890639
ENSG00000004468	CD38	64.9539424107838	1.50676270181429	0.425578489968854	3.54050483595767	0.000399362316416532	0.0180998693890639
ENSG00000004848	ARX	31.1641205020099	2.83043426182693	0.801692369071786	3.53057403440681	0.00041465895077577	0.0186663769841114
ENSG00000005844	ITGAL	159.759486166112	1.35479016028663	0.350399371807651	3.86641720645075	0.000110445934797443	0.00762260009662794
ENSG00000005961	ITGA2B	8.58294457063292	1.49378237226697	0.453091935782259	3.29686373624808	0.000977709234734282	0.032920603127495
ENSG00000007129	CEACAM21	17.8267704082821	1.37274322336356	0.363858917624384	3.77273486197927	0.000161467850863829	0.00991425265639193
ENSG00000007264	MATK	36.0032880206671	1.17113441176524	0.366453234498271	3.19586321394788	0.00139413187912624	0.0424574558316833
ENSG00000007306	CEACAM7	4645.98756142561	-2.73909591064156	0.656939361301871	-4.16948058221605	3.05294624259343e-05	0.00348286798744083
ENSG00000008118	CAMK1G	6.38854040633893	1.46459437498824	0.426323924265999	3.43540273398879	0.000591673816257239	0.0233821032123943
ENSG00000008513	ST3GAL1	679.24189009257	1.36949956110906	0.361139850292329	3.79215852252388	0.000149343528521042	0.00939848543216552
ENSG00000008517	IL32	6678.0839061446	0.767030330859585	0.245344313775993	3.12634240041897	0.00176995343542468	0.0490351685946927
ENSG00000009790	TRAF3IP3	83.7192614611323	1.19614546462781	0.378380606874432	3.16122296675939	0.00157108186442258	0.0455493456721898
ENSG00000010610	CD4	287.793563370153	1.29999346536561	0.308671384680594	4.21157752187109	2.53593495655042e-05	0.00310577445855176
ENSG00000011600	TYROBP	328.785959949116	1.29352799515559	0.36415493610989	3.55213637627377	0.000382116757368506	0.0175326676812251
ENSG00000011677	GABRA3	4.28209079018535	3.85234860984152	1.21865928914991	3.16113670501685	0.00157154722900367	0.0455493456721898
ENSG00000013725	CD6	129.703718817511	1.08834266387576	0.303864430699073	3.58167180466601	0.000341402510546473	0.0163089872342124
ENSG00000015285	WAS	123.183139829085	1.52442929027065	0.345186226284956	4.4162517916118	1.0042709632526e-05	0.00177674143500766
ENSG00000016391	CHDH	1284.57450587789	-0.718176455842048	0.203906065579191	-3.52209461647	0.000428151304095686	0.0189998795408999
ENSG00000016602	CLCA4	391.409868090609	-3.12503188774441	0.734754591920254	-4.25316414774251	2.10770957869223e-05	0.00272844643077963
ENSG00000019582	CD74	14854.6480404132	1.28229613165664	0.372561190366199	3.44184033338588	0.000577771168650422	0.0230591611462654
ENSG00000020256	ZFP64	497.764132251601	-0.472164523205195	0.137951950100392	-3.42267378505041	0.000620084455770186	0.0240188992914145
ENSG00000023445	BIRC3	1034.28361073015	1.52586462877471	0.339549910461872	4.49378598480309	6.99679481596581e-06	0.00145673268068408
ENSG00000023902	PLEKHO1	481.272605008649	1.11480807983382	0.280413595613847	3.97558498329375	7.02064669535313e-05	0.0055533762390331
ENSG00000025293	PHF20	977.398158925903	-0.50820746650415	0.162523526788406	-3.12697783851195	0.00176613239894759	0.0490351685946927
ENSG00000025708	TYMP	2344.99801512857	1.38424926812976	0.276432298477587	5.00755257527186	5.51264964545273e-07	0.000275456077483982
ENSG00000026751	SLAMF7	169.108517894869	1.98208704438445	0.383926357559121	5.16267509473931	2.43445478617952e-07	0.000168951162160858
ENSG00000027075	PRKCH	209.722643647883	0.787151436521589	0.231964666645086	3.39341093583859	0.000690280012443317	0.0256637191645867
ENSG00000029153	ARNTL2	683.492920146863	0.981708725874976	0.267774961754923	3.66617072575094	0.000246209585036025	0.0130932240415779
ENSG00000034533	ASTE1	134.251920448953	-0.56426006547293	0.169427230796079	-3.33039773371535	0.000867220128642646	0.0302676034639377
ENSG00000039068	CDH1	10427.1073573756	-0.736502681849722	0.169819105983336	-4.33698362492847	1.44451381674559e-05	0.00210168826764897
ENSG00000043462	LCP2	342.765407264269	1.37241833679181	0.345624458066966	3.97083685705454	7.16206123269535e-05	0.00559177930742689
ENSG00000044012	GUCA2B	15.7342549263051	-2.15030283940982	0.669458473341342	-3.21200332065023	0.00131812851734851	0.0412166744398437
ENSG00000047457	CP	40.9663498219854	1.74087014006572	0.552731369095855	3.14957724023046	0.00163506879950158	0.046368649613897
ENSG00000049249	TNFRSF9	56.9472610992171	1.12778081722551	0.298934093504419	3.77267378238621	0.000161507397980768	0.00991425265639193
ENSG00000049768	FOXP3	42.3483586190037	1.10412551516569	0.328280802835844	3.36335693597596	0.000770007350534684	0.0276406086864347
ENSG00000056558	TRAF1	227.694482678138	0.836628839167948	0.241780479044435	3.46028282545585	0.000539608305533179	0.0221009408285917
ENSG00000056736	IL17RB	461.68935760598	-0.843506045481734	0.255310700701987	-3.30384133200246	0.000953697924506018	0.0323738980242641
ENSG00000057019	DCBLD2	991.484003810583	1.99174151016897	0.377977359169011	5.26947305666096	1.36815950622506e-07	0.000110657821713214
ENSG00000057657	PRDM1	399.211119869034	0.848114410768352	0.230105523802108	3.68576293499905	0.000228018530338115	0.0124857376831952
ENSG00000059804	SLC2A3	907.238828678117	1.28057759576635	0.404704389424359	3.16422956911292	0.00155494091880899	0.0453309730636218
ENSG00000066294	CD84	99.4912971334111	1.52651845433488	0.353411821503927	4.31937575783079	1.56471168109654e-05	0.00222117935457478
ENSG00000066336	SPI1	320.356806357639	1.41541352961028	0.334258318369157	4.23449006898639	2.2907090729945e-05	0.00289045835756034
ENSG00000066405	CLDN18	18.0130340810606	2.84496759653402	0.766607907308649	3.71111172923056	0.000206350993308145	0.0115593569883648
ENSG00000066697	MSANTD3	321.560806027978	0.660943805545681	0.169595211215029	3.89718436511555	9.73175337524783e-05	0.0068683086533105
ENSG00000067066	SP100	969.105375869254	0.520604894622057	0.148836422215281	3.49783263312418	0.000469055386504965	0.0201487509159901
ENSG00000070388	FGF22	5.94939885128974	1.05752538672077	0.331000445417815	3.19493644603977	0.00139861635714799	0.0424574558316833
ENSG00000071246	VASH1	296.381356890759	1.07791224939491	0.307587090793092	3.50441316186445	0.000457614784985563	0.0197461965251802
ENSG00000072071	ADGRL1	1141.14217851411	-0.789740042913883	0.236954870227438	-3.33287111657955	0.000859547499619863	0.0302038463157562
ENSG00000072694	FCGR2B	93.1242047977268	1.74316631712887	0.44352974646876	3.93021286848828	8.48706902248032e-05	0.00629201579993022
ENSG00000072818	ACAP1	199.83050417959	0.993338794138011	0.311298283404172	3.19095493645339	0.00141803395860904	0.0427957962569227
ENSG00000073861	TBX21	15.9235930285927	1.46672318351543	0.427732750210245	3.42906448663211	0.00060566560279184	0.0237076277771692
ENSG00000074660	SCARF1	155.862861228891	1.0245820744459	0.26992717020331	3.79577229544614	0.000147184546530917	0.00933657741613086
ENSG00000075043	KCNQ2	24.1981910620184	3.3026232167792	0.846138734739399	3.90316987177802	9.49410182183645e-05	0.00675785298908153
ENSG00000075213	SEMA3A	369.75528742555	1.31238132933511	0.394301319909193	3.32837163628403	0.000873552507396662	0.0303544309385232
ENSG00000077420	APBB1IP	75.2254407944842	1.20336657743835	0.305604596918034	3.93765862678139	8.22805380074133e-05	0.00617326414888052
ENSG00000077984	CST7	89.5268184030592	1.88175698416899	0.40157928437432	4.68589157207364	2.78743813493625e-06	0.000800475337508591
ENSG00000078142	PIK3C3	416.045914690376	0.672541333003545	0.184580225958114	3.64362612253038	0.000268823831669537	0.0137348924739792
ENSG00000079215	SLC1A3	57.0064424021395	1.51918103168242	0.425602663328618	3.56948196658587	0.00035768783750788	0.0167349680380091
ENSG00000079263	SP140	43.4532591365791	1.28007595843239	0.308874880174375	4.14431875363166	3.40825395309275e-05	0.00368221921613172
ENSG00000080189	SLC35C2	1294.66156530561	-0.487109514825197	0.15007655011304	-3.24574035356156	0.00117145629347251	0.0374350363080741
ENSG00000080839	RBL1	398.955458230433	-0.899769076063514	0.227774958430547	-3.95025459454916	7.80681065978547e-05	0.00598298642711903
ENSG00000081237	PTPRC	449.976357590749	1.32523571137962	0.352894040559605	3.75533604726823	0.000173109052163827	0.0102749009547565
ENSG00000082074	FYB1	168.488789591143	1.24761774609537	0.31201848332972	3.99853794807716	6.37349635377277e-05	0.00520377231708036
ENSG00000085265	FCN1	42.0121459111027	1.85981121769941	0.567486024658518	3.27728109043485	0.00104811969351927	0.0344555558195861
ENSG00000086300	SNX10	329.875417378507	1.08678653740173	0.293474302420959	3.70317444640466	0.000212918363275294	0.0118400248872546
ENSG00000086730	LAT2	216.160238575726	1.91005488623369	0.396860297311925	4.81291502115774	1.48744549489155e-06	0.000509073126635213
ENSG00000087128	TMPRSS11E	3.46384357401286	4.02215513442986	0.948973605727453	4.23842676988535	2.25091579022637e-05	0.00286453445850951
ENSG00000087589	CASS4	18.3696511910841	1.33433958649224	0.353869365543586	3.77071234872942	0.0001627822210751	0.00996553722974346
ENSG00000088726	TMEM40	6.2705636149449	2.4821768366167	0.659845790118308	3.76175293347201	0.000168726675960751	0.0101354053354664
ENSG00000088827	SIGLEC1	87.0431598335352	1.25697087162213	0.403456844092899	3.11550266162472	0.00183631691461439	0.0498547522664581
ENSG00000089012	SIRPG	32.1881922988234	1.30965113441977	0.410549281624107	3.18999738408717	0.00142274081542955	0.0427957962569227
ENSG00000089041	P2RX7	45.7540708026856	1.21498893527658	0.359499097410267	3.37967172665809	0.000725724540973197	0.0265080437597578
ENSG00000089692	LAG3	65.0047979676006	1.65390142796271	0.360222723554604	4.59133008501614	4.40430127190137e-06	0.00108947587106123
ENSG00000090104	RGS1	693.870413397062	1.4319939912674	0.381327029938751	3.75529107259301	0.000173140141768832	0.0102749009547565
ENSG00000090339	ICAM1	879.985359084657	1.23224476539398	0.26065431998815	4.72750563063753	2.27294762844229e-06	0.000687577357793675
ENSG00000090659	CD209	44.7595284167746	1.17120910387388	0.350487052710697	3.341661538752	0.000832785417670165	0.0295544188566355
ENSG00000091129	NRCAM	55.8478671797979	1.1478428670336	0.361517316861776	3.1750702206956	0.00149800245517346	0.0441159353872439
ENSG00000091138	SLC26A3	1412.84090513449	-3.22163175659498	0.622660492960319	-5.17397810366667	2.2916169160151e-07	0.000163582162942061
ENSG00000091844	RGS17	58.2883845504844	1.75293669921261	0.383547130500221	4.57032932804486	4.8695836498812e-06	0.00112649701767252
ENSG00000092054	MYH7	2.6260022934299	2.78579879045154	0.66560827076994	4.18534281016232	2.8473605356326e-05	0.00331635301364921
ENSG00000093072	ADA2	206.041376937805	1.23635193779411	0.317637310584753	3.89233851501278	9.92825826751426e-05	0.00692870403786526
ENSG00000093217	XYLB	194.338638984781	-0.52053113845729	0.15668464315819	-3.32215798539847	0.000893241026952515	0.0308668517529483
ENSG00000094963	FMO2	8.42938495183112	2.20306211878391	0.538495975214937	4.09113943313053	4.29258938302901e-05	0.00422228555691326
ENSG00000095370	SH2D3C	121.007189398398	1.06782370674314	0.301372063297585	3.54320733998735	0.000395291783505616	0.017956308943826
ENSG00000095627	TDRD1	3.3354345364015	3.3841240557307	0.795529240818593	4.25392792884452	2.10052933350375e-05	0.00272844643077963
ENSG00000095932	SMIM24	573.013825346701	-1.31122707247151	0.38413936402957	-3.4134150135433	0.000641541643889683	0.0243590827217931
ENSG00000096968	JAK2	350.086972714881	0.84286081364627	0.222663485133027	3.78535714170968	0.000153487987945922	0.0095868597271023
ENSG00000096996	IL12RB1	71.4816591222727	1.29169546633843	0.320308926020579	4.03265523189148	5.5150161311963e-05	0.00476772190386881
ENSG00000099250	NRP1	650.774920990859	1.02503539367527	0.295131274207218	3.4731507070158	0.000514386427899546	0.0213664358695024
ENSG00000099381	SETD1A	803.9426703866	-0.490442807356625	0.143007630798713	-3.42948697644629	0.000604723453898505	0.0237076277771692
ENSG00000099822	HCN2	16.6571487155186	1.15079657429995	0.369218173181166	3.11684705112087	0.0018279638230434	0.0497024505499483
ENSG00000099834	CDHR5	2623.59963074877	-1.50394210060156	0.377025004129151	-3.98897177675352	6.63603156735959e-05	0.00535977185764146

ENSG00000099985	OSM	149.694142997882	2.01485179137501	0.485047595622194	4.15392594368076	3.2681898607194e-05	0.00362475419726277
ENSG00000100034	PPM1F	540.602459598699	0.471923291496669	0.146031625348728	3.23165129724264	0.00123077148367499	0.038874329643661
ENSG00000100055	CYTH4	174.951800160283	1.46087030593236	0.371421069584243	3.93319180187493	8.38252971544473e-05	0.00627033300630752
ENSG00000100336	APOL4	212.177053484559	1.2865855672272	0.287875637133561	4.46924088484183	7.84976885161084e-06	0.00155649702371941
ENSG00000100342	APOL1	2476.97981514855	1.32261975491439	0.358993801436829	3.68424120310926	0.000229384950231612	0.0124857376831952
ENSG00000100351	GRAP2	58.3295495013398	1.48033377618395	0.396105872430149	3.73721744416956	0.000186068002416929	0.0108361840848125
ENSG00000100365	NCF4	174.526745303315	1.28654757935239	0.31595720109684	4.07190459621166	4.66302793111457e-05	0.00437974021920927
ENSG00000100368	CSF2RB	186.645288167779	1.59758597600619	0.335682034696301	4.75922394074964	1.94338724158151e-06	0.000606919835545906
ENSG00000100385	IL2RB	153.933138843744	1.33775111139645	0.313929800983977	4.26130653159852	2.03235248670411e-05	0.0026584447396762
ENSG00000100450	GZMH	54.2453642443164	1.89641938768088	0.502163954973754	3.77649444747582	0.000159051103567619	0.0098359722067658
ENSG00000100625	SIX4	70.3404550795158	1.40668552136347	0.298649065367252	4.71016214175541	2.47519797469906e-06	0.000736194597617635
ENSG00000100644	HIF1A	2598.78874589581	0.775121013797425	0.19240176893516	4.02913142684605	5.59833148650608e-05	0.0047900244472215
ENSG00000100906	NFKBIA	1904.84926349238	0.991888697932198	0.217884769153116	4.55235444766293	5.30488613595732e-06	0.00118336852875677
ENSG00000100983	GSS	1924.8732973972	-0.647728793131821	0.150869177552935	-4.29331427159503	1.76025569074114e-05	0.00245688425572495
ENSG00000100991	TRPC4AP	1833.8929010636	-0.442750354538961	0.106663588293938	-4.15090436784156	3.31164125648389e-05	0.00364484780405258
ENSG00000101017	CD40	171.147056184452	1.11453185403303	0.353727119596093	3.15082387605922	0.00162810635028005	0.0463030693713705
ENSG00000101019	UQCC1	894.364843193977	-0.596342208862997	0.186949644923102	-3.18985472857299	0.00142344327127372	0.0427957962569227
ENSG00000101057	MYBL2	1632.22729207001	-1.0079764981554	0.277781845891066	-3.6286622508466	0.000284893674773015	0.0142640953317214
ENSG00000101076	HNF4A	4375.34558504034	-1.0882251145632	0.225348459710794	-4.82907722537708	1.37167208632664e-06	0.000496664571083836
ENSG00000101082	SLA2	34.056040357649	1.27855718975609	0.346997479040718	3.68462962120268	0.000229035446523266	0.0124857376831952
ENSG00000101126	ADNP	2102.8925074901	-0.399479734502291	0.128165484411972	-3.11690574365727	0.00182759994554799	0.0497024505499483
ENSG00000101138	CSTF1	608.031843228667	-0.514730178540841	0.148139250675707	-3.47463738471071	0.000511544340107611	0.0213362667666921
ENSG00000101158	NELFCD	1920.28556613161	-0.705499753274194	0.205634435118371	-3.43084441508098	0.000601705599937392	0.0236368124352764
ENSG00000101161	PRPF6	2523.95121657928	-0.543510474439201	0.173153367010478	-3.13889636582297	0.00169585412069608	0.0477572664603769
ENSG00000101188	NTSR1	223.331682939225	2.73823308579102	0.795191827945471	3.44348745744252	0.000574263230035282	0.0230241777896857
ENSG00000101191	DIDO1	1801.11677003927	-0.620857140109545	0.19280370665249	-3.22015147368811	0.00128122879078904	0.0401633878407444
ENSG00000101210	EEF1A2	108.579057907059	2.77213059534271	0.613190714518005	4.52082937609668	6.15978222172512e-06	0.00131535041903915
ENSG00000101331	CCM2L	35.4847175672537	0.999708779233209	0.317164954816519	3.15201526540517	0.00162147796025514	0.0462454399075507
ENSG00000101333	PLCB4	1632.4272063081	-1.34499381254902	0.343293920046368	-3.91790746648631	8.93209791485698e-05	0.00648719576467403
ENSG00000101336	HCK	168.929954255819	1.36745989561575	0.299648124199804	4.56355233081298	5.02952657797849e-06	0.00114234265476559
ENSG00000101337	TM9SF4	2792.44838142254	-0.598190996903285	0.160986019588171	-3.7157946909524	0.000202566019384279	0.0114759850981788
ENSG00000101346	POFUT1	2343.59878197953	-0.98887616705803	0.227146217805159	-4.35347846252172	1.33994265017048e-05	0.00205726877171705
ENSG00000101350	KIF3B	1573.81111134097	-0.60825024211731	0.161736596966372	-3.76074588884651	0.000169407457269757	0.0101354053354664
ENSG00000101407	TTI1	533.860315012793	-0.725181220471975	0.185038255013227	-3.91908808489433	8.88842885672917e-05	0.00648719576467403
ENSG00000101413	RPRD1B	948.947896298266	-0.756472289320671	0.152840223208924	-4.94943194303383	7.44303979946895e-07	0.000326240186578829
ENSG00000101417	PXMP4	382.652564380462	-0.639702977086813	0.200346085089081	-3.19292965488832	0.00140807990972559	0.0425901555261309
ENSG00000101444	AHCY	6464.7823867281	-0.683220847014799	0.199410777900188	-3.42619819354386	0.00061209357350715	0.023820164860596
ENSG00000101445	PPP1R16B	106.397187949328	1.08993512993735	0.328249983703931	3.32044229717444	0.00089874936051855	0.0309736918875359
ENSG00000101452	DHX35	339.157921894765	-0.698520456183437	0.173497489789978	-4.02611275372716	5.67065055826792e-05	0.00483534244190326
ENSG00000101464	PIGU	875.231126459197	-0.76536443971122	0.175296880095882	-4.36610417306109	1.26482175110899e-05	0.00200001940694348
ENSG00000101470	TNNC2	69.3279205687405	-1.67906899261909	0.510253914345357	-3.29065382040878	0.00099954837204823	0.0330764457314609
ENSG00000101624	CEP76	143.900205500922	0.540530309308676	0.169616629495127	3.18677662041506	0.00143867840665132	0.043031911545288
ENSG00000101745	ANKRD12	668.295205015723	0.617703575728325	0.181150209858567	3.40989710257912	0.000649873932992377	0.0244893670314956
ENSG00000101916	TLR8	21.201354484292	1.94075692786038	0.383797195340753	5.05672514395862	4.26517264482151e-07	0.000242184257632319
ENSG00000102096	PIM2	494.749935604325	0.982176223545979	0.220845166710351	4.44735213442163	8.69352619306673e-06	0.00163307562712466
ENSG00000102524	TNFSF13B	99.2226328385263	1.30766169074563	0.342960623259169	3.8128624747613	0.000137366555856767	0.00887784921164335
ENSG00000102575	ACP5	594.75560175204	0.987796642382306	0.253320473526228	3.89939521520764	9.64332592379744e-05	0.00682518002493358
ENSG00000102755	FLT1	629.847940634323	0.932918857899957	0.295225452710359	3.16002177093867	0.00157757350422892	0.0455654293984454
ENSG00000102924	CBLN1	26.2223127863798	-2.00554240701865	0.586330234488877	-3.42049972703002	0.000625061984590753	0.0240254594200237
ENSG00000102970	CCL17	12.5990663519151	1.84310110873192	0.578255005012747	3.18733003200066	0.00143582895603779	0.0430128904528156
ENSG00000103375	AQP8	38.2011056983619	-2.29734411364829	0.6388159459951	-3.59625355010457	0.000322833147708397	0.0156919520668221
ENSG00000103522	IL21R	56.5634874795183	1.66147997221885	0.371961015415246	4.46681212106067	7.93937965144452e-06	0.00156179450487111
ENSG00000103647	CORO2B	23.6275756995905	2.03147869587052	0.459724293744898	4.41890655662803	9.9201524267117e-06	0.00177674143500766
ENSG00000104044	OCA2	13.5060776337285	-3.00237820184025	0.727049683140049	-4.12953649724913	3.63495298836646e-05	0.00384812141785372
ENSG00000104327	CALB1	118.106671740445	2.82274883694126	0.583362058083943	4.83875973389939	1.30651874081534e-06	0.000480030356184273
ENSG00000104518	GSDMD	2053.04263021648	0.560839578285553	0.172438011860695	3.25241269157423	0.00114429744635535	0.0367942437577118
ENSG00000104814	MAP4K1	96.3183721141613	1.44179939094772	0.368692668071061	3.91057245182273	9.20776347677802e-05	0.0065915977852098
ENSG00000104892	KLC3	29.6259953986915	1.54906416077781	0.437148773104573	3.54356286940154	0.000394759176954653	0.017956308943826
ENSG00000104903	LYL1	69.2090833792205	0.852164932423812	0.261461141002793	3.25924123621379	0.00111710635117638	0.0360126259068267
ENSG00000104951	IL4I1	95.4944666731715	1.39805005660105	0.327450950933765	4.26949456892505	1.95916419626496e-05	0.00257619780418335
ENSG00000104972	LILRB1	80.9236388577537	1.71988602394032	0.383801910281124	4.48118151022374	7.42309449454847e-06	0.0015327156434033
ENSG00000105122	RASAL3	173.263840946441	1.28401268308054	0.353851476656368	3.62867691047526	0.000284877500209908	0.0142640953317214
ENSG00000105135	ILVBL	1795.934639547	-0.553928317629387	0.155424249271885	-3.56397615059665	0.000365279158568094	0.0169946638690228
ENSG00000105374	NKG7	104.606482220234	1.74170371138247	0.419236217946453	4.15446861894202	3.2604435916944e-05	0.00362475419726277
ENSG00000105398	SULT2A1	7.18365932184504	-3.72286353948346	0.867681000668942	-4.29059013233354	1.78198941479069e-05	0.00245973610713429
ENSG00000105639	JAK3	281.258987270895	1.00912102486234	0.292379872707103	3.45140387236316	0.000557678374121138	0.022545366503305
ENSG00000105967	TFEC	59.2201268922069	1.47180132344856	0.396445234924532	3.71249593586044	0.000205225357266921	0.0115593569883648
ENSG00000105971	CAV2	541.785839354113	1.30784744791449	0.328017887999501	3.98712233619553	6.68795445907452e-05	0.00537272843104559
ENSG00000105974	CAV1	641.466851560884	0.902004984566301	0.266866390106604	3.37998720710383	0.000724892037779286	0.0265080437597578
ENSG00000105982	RNF32	87.8357641146031	-0.849049008159897	0.235409120128938	-3.60669547422316	0.000310121256818244	0.0152505547330096
ENSG00000106123	EPHB6	205.936888645792	2.37223299434557	0.647366146446822	3.66443782605249	0.000247882443630577	0.0130932240415779
ENSG00000106384	MOGAT3	370.346260450511	-1.11704252902354	0.350589510090738	-3.18618354763221	0.00144163104240338	0.043031911545288
ENSG00000106404	CLDN15	659.475739247808	-1.70392264288766	0.420274970454734	-4.05430435470384	5.0283713951549e-05	0.0045849938298011
ENSG00000106809	OGN	13.5693831356516	-2.10363374773004	0.673032036750131	-3.12560715220609	0.00177438413655694	0.0490389527297992
ENSG00000108001	EBF3	14.2218647636427	1.2392701342492	0.356315627917938	3.47801229345633	0.0005051456757002471	0.0212110698772265
ENSG00000108576	SLC6A4	140.367023041674	-3.03973304125838	0.611792676516641	-4.96856722536412	6.74494170424694e-07	0.000312065969516492
ENSG00000108622	ICAM2	224.385761991206	0.862683062546644	0.254539225310515	3.38919497179363	0.000700981493238622	0.0259841567167266
ENSG00000108688	CCL7	8.3334175107799	2.72779338689238	0.70646582430316	3.86118237153652	0.000112839635871289	0.00770269252078768
ENSG00000108691	CCL2	208.904042314003	1.43694729859531	0.424408690168747	3.38576313794135	0.000709806211367576	0.0261701212318705
ENSG00000108700	CCL8	25.9769400408376	2.225827132989	0.546415776589143	4.07350451497418	4.63109533114072e-05	0.00437974021920927
ENSG00000108950	FAM20A	73.3358188933362	1.93426081258464	0.378259677226463	5.11357918657189	3.16111150212951e-07	0.000200067637467126
ENSG00000109790	KLHL5	254.061443549665	0.977639064271952	0.284893161497859	3.43159891635131	0.000600034261432844	0.0236082771458869
ENSG00000109861	CTSC	2961.02410805087	0.834931582773341	0.202769376560011	4.11764141576988	3.82769653174208e-05	0.00398951790871951
ENSG00000109943	CRTAM	15.8923838855688	1.83107829293454	0.505200462324197	3.62445886235058	0.000289567104045785	0.0143604116434913
ENSG00000109956	B3GAT1	13.8600598699255	1.97824671496598	0.505374372982398	3.9144183415784	9.06223994595972e-05	0.00656263776260457
ENSG00000110031	LPXN	197.678231835028	1.26272825659316	0.24746246755345	5.10270615611809	3.34830682506904e-07	0.000200067637467126
ENSG00000110077	MS4A6A	223.55936660417	1.1829062858158	0.32637767603474	3.6243480258432	0.000289691301165531	0.0143604116434913
ENSG00000110079	MS4A4A	78.1328995750159	1.31753390678666	0.355823712533547	3.70277151403292	0.000213256932407323	0.0118400248872546
ENSG00000110218	PANX1	283.639141734471	0.602484044528412	0.131187501735981	4.59254148875348	4.37880421324371e-06	0.00108947587106123
ENSG00000110324	IL10RA	190.800468995457	1.3176921228717	0.300097090574542	4.39088603074741	1.12889703111912e-05	0.00186783863744901
ENSG00000110665	C11orf21	15.753419233535	1.68542060398024	0.456915466838495	3.68869238689173	0.000225409548991431	0.012404476149784
ENSG00000110756	HPS5	306.354806698546	0.493081438993894	0.127701792956155	3.86119433078898	0.00011283411986228	0.00770269252078768
ENSG00000110848	CD69	71.1068904218046	1.32125336072847	0.400439443177922	3.2995085355301	0.000968542821316363	0.0327000997942811
ENSG00000110852	CLEC2B	145.917928885175	1.25703117861531	0.373575849017852	3.36486200036782	0.000765819513726309	0.0275695024941471
ENSG00000110876	SELPLG	207.206625527864	1.14128867315583	0.312031465215399	3.65760764661342	0.00025458035165726	0.0133622594659768
ENSG00000110944	IL23A	48.618764563023	1.57930807001217	0.420273466213184	3.75781056140019	0.000171406565641475	0.0102205766968654
ENSG00000111058	ACSS3	56.9029056898888	1.7352427195612	0.427145501928962	4.06241599577886	4.85674225581149e-05	0.00447752254653854
ENSG00000111252	SH2B3	521.899922465962	0.926698489342175	0.187199993876541	4.9503126049958	7.40943711803241e-07	0.000326240186578829

ENSG00000111537	IFNG	13.8034036256016	2.11192975600396	0.576269120486255	3.66483242104299	0.00024750058570724	0.0130932240415779
ENSG00000111729	CLEC4A	35.1580806314732	1.3189830482656	0.322690121577071	4.08746026007671	4.3612135235525e-05	0.00422498284918415
ENSG00000111817	DSE	209.956969369976	1.05320939609836	0.252149726073793	4.17692064353119	2.95482053512448e-05	0.00340391320622509
ENSG00000112096	SOD2	8182.6609070215	0.909942038352914	0.215591066693639	4.22068526450574	2.43560793943673e-05	0.00299759747580725
ENSG00000112208	BAG2	341.771440833339	1.24954218162053	0.295720539863393	4.2254155974345	2.38500205196424e-05	0.00297934456331373
ENSG00000113088	GZMK	57.5806230403908	1.73047811179255	0.539219403145008	3.20922819486744	0.00133091816689338	0.041512683497708
ENSG00000113231	PDE8B	57.4730775789709	0.906315032820421	0.224329628159708	4.04010402128061	5.34274996789516e-05	0.00468362334027694
ENSG00000113263	ITK	59.6302903711057	1.21174796777696	0.368226626035928	3.29076683243087	0.000999146926899358	0.0330764457314609
ENSG00000113303	BTNL8	202.414093634855	-1.63397048126343	0.496167726992586	-3.29318170524188	0.000990604324920845	0.0330136987358669
ENSG00000113532	ST8SIA4	126.550263669048	1.11566441273971	0.268629002815737	4.15317929577761	3.27887627514164e-05	0.00362475419726277
ENSG00000113749	HRH2	27.2866994764206	1.35094458783572	0.394246591760655	3.42664874235837	0.000611078981797769	0.0238177804699461
ENSG00000114013	CD86	82.2520247991628	1.3047236686152	0.295460924446277	4.41589246043409	1.00594088519721e-05	0.00177674143500766
ENSG00000114113	RBP2	76.6954137730057	-2.7964160106746	0.830005619938831	-3.3691531039039	0.000753995342287713	0.0272222827047922
ENSG00000114650	SCAP	1630.54316228217	-0.429605979578127	0.131353669827658	-3.27060507819682	0.00107317664150045	0.0350945617948261
ENSG00000115008	IL1A	53.2405033312345	1.58152599795509	0.412361261368113	3.83529236647491	0.000125415018156045	0.0082893354857424
ENSG00000115085	ZAP70	146.730459756204	1.39044235924129	0.418171444987815	3.32505333854588	0.000884016211116077	0.0306328169466353
ENSG00000115165	CYTIP	155.923067594132	0.985663129302421	0.310841527748057	3.17095060123794	0.0015194097897179	0.0446599225721318
ENSG00000115232	ITGA4	113.658717481981	1.019788178328	0.290952674282111	3.50499675194325	0.00045661285391695	0.0197461965251802
ENSG00000115295	CLIP4	69.2934151354364	1.1916815419828	0.378500579859123	3.14842725584818	0.00164151573692662	0.0464984457725335
ENSG00000115355	CCDC88A	258.109826428518	0.955069938176478	0.261265626609945	3.65555144229648	0.000256629738898469	0.0133854642936103
ENSG00000115386	REG1A	7928.88062525336	3.7714754021586	0.957226370645574	3.94000365829353	8.14803665822723e-05	0.00615016760933985
ENSG00000115415	STAT1	3671.87578380023	1.2288946372996	0.225279388844568	5.45498034064482	4.89782639503179e-08	4.89469178613897e-05
ENSG00000115523	GNLY	171.060303904192	1.70379606120296	0.425367582455764	4.00546758021964	6.18949229345964e-05	0.00512047269734423
ENSG00000115919	KYNU	309.780794819363	1.6121853891642	0.448676345087558	3.59320346574006	0.00032663733080254	0.0157847332161908
ENSG00000115956	PLEK	271.362830400208	2.02304163302639	0.409150481945022	4.94449285116136	7.63423428526646e-07	0.000328851223074306
ENSG00000116141	MARK1	71.4273214566746	1.91725712107001	0.536094748731451	3.57634005109501	0.000348438345593983	0.0164793224025849
ENSG00000116701	NCF2	188.16256619012	1.35349181149172	0.339842928790892	3.98269817267414	6.81372669316559e-05	0.00545622268275798
ENSG00000116741	RGS2	398.686026071861	0.985788868191988	0.309864488138801	3.18135477257533	0.00146587997486928	0.0435994586811121
ENSG00000116793	PHTF1	198.202316206071	0.572163810601019	0.180064794725287	3.17754401394193	0.00148528143837136	0.0439671462752014
ENSG00000116824	CD2	133.919496172956	1.17136418736793	0.370381599253833	3.16258742261428	0.00156373775830874	0.0454284001785879
ENSG00000117090	SLAMF1	30.9310330310446	1.23731366644418	0.375517559024397	3.29495555323365	0.000984372480496326	0.0330114926882151
ENSG00000117091	CD48	136.09887811974	1.24437477014035	0.398348660741136	3.12383319633903	0.00178511623384395	0.0491693731874959
ENSG00000117115	PADI2	762.54310591012	-1.82259327099648	0.487218628404829	-3.7408119573829	0.000183426721425735	0.0107247177025167
ENSG00000117228	GBP1	1038.03461757356	1.47609541756305	0.301839054294994	4.89033939299461	1.00662263178964e-06	0.000405636448913425
ENSG00000117525	F3	1899.52426779404	1.41554618790492	0.346322299372271	4.08736656712744	4.36297460410467e-05	0.00422498284918415
ENSG00000117560	FASLG	15.0569113473255	1.5100550177535	0.478859294635277	3.15344201244676	0.00161357282321713	0.0461252899488063
ENSG00000117834	SLC5A9	59.7350356265547	-1.74723983983459	0.407920437262669	-4.28328585730923	1.84153331469154e-05	0.00248696585590559
ENSG00000118242	MREG	240.786619931378	0.648030167876889	0.180796246024776	3.58431207575009	0.000337967797657452	0.0161758380396049
ENSG00000118292	C1orf54	123.248505794931	1.05694432491689	0.316519909956411	3.33926647793638	0.00083999942848884	0.0297259854410272
ENSG00000118503	TNFAIP3	1023.38500219223	0.860326569715588	0.241394420428361	3.56398697280954	0.000365264090430657	0.0169946638690228
ENSG00000118705	RPN2	8252.82416875894	-0.558930901283886	0.165072299361172	-3.38597634761824	0.000709254964321205	0.0261701212318705
ENSG00000118985	ELL2	529.48881998669	0.862406537303118	0.209456244897999	4.11735891533381	3.83238992192076e-05	0.00398951790871951
ENSG00000119139	TJP2	2565.57886857086	-0.402163367014919	0.118038822408336	-3.40704319824286	0.000656707324739456	0.0247096021103774
ENSG00000120217	CD274	82.4880889620337	1.85316557173865	0.314271797235625	5.89669702480251	3.70849885904327e-09	9.99982434254387e-06
ENSG00000120279	MYCT1	69.4275804524214	0.923574027145971	0.288028282026609	3.2065393740072	0.00134341918754839	0.0417982378352541
ENSG00000120280	CXorf21	13.6865452148768	1.17045225092854	0.365888630370507	3.19893036780976	0.00137938480832558	0.0423372850751919
ENSG00000121057	AKAP1	3527.07295498283	-0.558852917251017	0.158878366712585	-3.51748906295088	0.000435650303181703	0.0191962736767049
ENSG00000121064	SCPEP1	470.231967171274	0.716494524723709	0.207980142860085	3.44501410024378	0.000571029603271449	0.0229694376377272
ENSG00000121210	TMEM131L	132.064654480329	0.721161977992662	0.204293700943178	3.53002552043073	0.000415519584124966	0.0186714771398888
ENSG00000121552	CSTA	33.8517903913017	1.27407610704196	0.404008966579643	3.15358373807478	0.00161278950799456	0.0461252899488063
ENSG00000121594	CD80	14.0837249243403	1.21318714888696	0.387317367963842	3.13228181649788	0.00173453294283277	0.0485014419087505
ENSG00000121742	GJB6	6.91700003456606	2.91403224712168	0.828749079235337	3.51618158032872	0.000437801454126984	0.0192570977639236
ENSG00000121743	GJA3	12.3021284932774	2.72369293126406	0.608538221375321	4.47579599044478	7.61271511114891e-06	0.00153383930916891
ENSG00000121807	CCR2	17.906785763044	2.01450638925171	0.451372675707426	4.46306676870598	8.07948629172422e-06	0.00156478981017394
ENSG00000122122	SASH3	150.814301578433	1.13644882462329	0.362332636404209	3.13647932988156	0.00170989489484637	0.0479461437181165
ENSG00000122133	PAEP	25.27617240183	3.09026744037651	0.71085663484512	4.34724428090878	1.3785865303014e-05	0.00207149779433149
ENSG00000122188	LAX1	18.7424116070434	1.39969448211953	0.414265611299106	3.37873439243454	0.000728203260938603	0.0265381642260818
ENSG00000122223	CD244	20.7564443611492	1.35596447658133	0.433977979741381	3.12450064261183	0.00178107132814823	0.0491693731874959
ENSG00000122548	KIAA0087	4.34406660055394	1.59605158718777	0.512441690990371	3.1146013590408	0.0018419365987621	0.0498580108163297
ENSG00000122862	SRGN	958.638351560645	1.34882752469129	0.342431555835273	3.93896970564285	8.18322609982644e-05	0.00615812412283325
ENSG00000123329	ARHGAP9	163.59258780532	1.29407512050286	0.343361338032232	3.76884341628355	0.000164005727176388	0.00996963281697053
ENSG00000123338	NCKAP1L	188.557829472348	1.3160206317389	0.325200429033405	4.04679857173164	5.19228844168965e-05	0.00463300480097051
ENSG00000123364	HOXC13	6.81309292085342	3.5072568308799	1.05781073618663	3.3155806713812	0.000914529412126527	0.0313854434513313
ENSG00000123454	DBH	14.4601432455679	3.15838324854994	0.641596666185747	4.92269276995018	8.53614101449103e-07	0.000361469401874651
ENSG00000123561	SERPINA7	5.93771337337016	-4.49514605423252	1.04920168458092	-4.28434887237911	1.83275136364697e-05	0.00248696585590559
ENSG00000123685	BATF3	51.1496649447913	1.73098589590167	0.311728196781114	5.55286917826402	2.81018388825699e-08	3.5853590899019e-05
ENSG00000124126	PREX1	416.50869149599	0.929828400710473	0.272147400966355	3.41663524034693	0.000634001645130426	0.0242200261497532
ENSG00000124217	MOCS3	356.330672907565	-0.833785104218121	0.199623314745231	-4.17679220126285	2.95648881584552e-05	0.00340391320622509
ENSG00000124228	DDX27	1945.15706662323	-0.703469514038487	0.21986606832613	-3.19953651508891	0.00137648749324539	0.0423003241466701
ENSG00000124253	PCK1	1692.4226343767	-1.71274629049604	0.519755914946839	-3.29528965662896	0.000983202785866849	0.0330114926882151
ENSG00000124469	CEACAM8	15.3368159254309	-1.72364853636481	0.488979183901748	-3.52499368707512	0.000423492883634771	0.0189276318510396
ENSG00000124491	F13A1	101.201023530457	1.43582702441691	0.438279918889612	3.27605021935434	0.00105269840557603	0.0345606004795157
ENSG00000124508	BTN2A2	208.20813829333	0.644283128670586	0.194967882074796	3.30456032970301	0.000951255001061101	0.03233490468913
ENSG00000125046	SSUH2	66.2463721907777	-1.57829782134168	0.483335736208309	-3.26542753433291	0.00109298968009065	0.035463479254351
ENSG00000125347	IRF1	2076.84221981139	1.0723741221801	0.234745300305732	4.56824533135887	4.9182416863992e-06	0.00112731514030273
ENSG00000125510	OPRL1	10.8082896941993	1.26938227174731	0.335026433911927	3.7889018395516	0.000151314683178235	0.00947480211660404
ENSG00000125538	IL1B	405.823076819825	1.69041762662072	0.453595174455659	3.72670989864325	0.00019399552514883	0.0111677055306875
ENSG00000125730	C3	2884.42728064888	1.99278813646977	0.459435636579431	4.33746966453536	1.4413241972126e-05	0.00210168826764897
ENSG00000125735	TNFSF14	31.677888299009	1.69097635119405	0.357277683504584	4.73294703046391	2.21283251056836e-06	0.000682535894370862
ENSG00000125967	NECAB3	574.829143558115	-0.606653269287813	0.184885223640978	-3.28124258575608	0.00103350813692615	0.0340648645025898
ENSG00000125991	ERGIC3	5055.83110149366	-0.654726803933243	0.124264410798431	-5.26881992781727	1.37303573211241e-07	0.000110657821713214
ENSG00000126001	CEP250	891.605566411802	-0.728228307909419	0.19597400683141	-3.7159433523033	0.000202446939620589	0.0114759850981788
ENSG00000126003	PLAGL2	1128.59974526679	-1.11400894332613	0.267949285737266	-4.1575365288283	3.21697848210832e-05	0.00362475419726277
ENSG00000126246	IGFLR1	98.0242128337333	0.703259477392337	0.222504034272086	3.16065944463895	0.00157412424624093	0.0455604941032831
ENSG00000126259	KIRREL2	2.32824385216016	2.33756542115755	0.616107426707014	3.79408739422511	0.000148187477003882	0.00934928264006307
ENSG00000126264	HCST	55.7574541803614	1.56221997654956	0.310542570645169	5.03061455730196	4.88910157625965e-07	0.000259892156981428
ENSG00000126860	EVI2A	99.5446139836963	1.44169429462868	0.36559606345812	3.94340759851706	8.03319513145282e-05	0.00610034489860843
ENSG00000126882	FAM78A	93.17270193996	1.26712589597207	0.282879914287024	4.47937740353801	7.48610713474873e-06	0.00153305656274231
ENSG00000127863	TNFRSF19	77.6854429708861	1.88016135051245	0.542142143249538	3.46802286803047	0.000524302669935081	0.0215801942432588
ENSG00000127951	FGL2	330.210265046275	1.49684805281144	0.36584350753909	4.09149820063843	4.2859527241535e-05	0.00422228555691326
ENSG00000128284	APOL3	381.819249185522	1.18071155383814	0.325368027311244	3.62884934821418	0.000284687307227329	0.0142640953317214
ENSG00000128335	APOL2	766.248251614979	0.98089551666743	0.261699491362411	3.74817509793723	0.000178125888314035	0.0105144781900598
ENSG00000128340	RAC2	478.36397273984	1.18360177115678	0.26139770779493	4.52797303060259	5.95521800152569e-06	0.00129378405695755
ENSG00000128394	APOBEC3F	66.2268185685745	0.890402604369063	0.277447865599104	3.20920096312322	0.00133076648298912	0.041512683497708
ENSG00000128512	DOCK4	167.91747287122	1.15420105789318	0.282811305449181	4.08117015004049	4.4809535781584e-05	0.00427298260292785
ENSG00000128791	TWSG1	799.220050494071	0.713701494469283	0.228302570828943	3.12612114650267	0.00177128566924713	0.0490351685946927
ENSG00000129173	E2F8	250.257569955611	-0.775854056932856	0.245912341006871	-3.15500252551855	0.00160496716058078	0.0460373129046501
ENSG00000129988	LBP	6.12091822022854	1.9041286368599	0.600842532553761	3.16909761492214	0.00152913029266263	0.0448382187020299

ENSG00000130054	FAM155B	31.9633136517913	-3.12731763953336	0.812534153063408	-3.84884454117132	0.000118676289634582	0.00797045274255485
ENSG00000130561	SAG	11.0999844500696	-1.30331282330458	0.387876775588981	-3.36012080466929	0.000779083979646204	0.0278862953402303
ENSG00000130584	ZBTB46	136.901491884562	0.942008643934604	0.238828112901191	3.94429547049665	8.00349283447051e-05	0.00609631905438739
ENSG00000130592	LSP1	460.157806376585	1.08551606775073	0.305068053402258	3.55827513121929	0.000373298183442254	0.017207531024209
ENSG00000130699	TAF4	796.772623537311	-0.677109157884165	0.192333920188998	-3.52048747937338	0.000430754329085429	0.019081500279912
ENSG00000130755	GMFG	201.066139553236	1.13811053352167	0.277054008208332	4.10790134703938	3.99270569001574e-05	0.00411222139431798
ENSG00000130775	THEMIS2	207.658706332577	0.99856615175226	0.307299056616944	3.24949305977532	0.00115610899246636	0.0370785970061353
ENSG00000131042	LILRB2	121.911160942016	1.57960097626566	0.359591746429616	4.39276204737596	1.1191960375168e-05	0.00186413292008799
ENSG00000131043	AAR2	771.910036674505	-0.682711195601748	0.149866642763756	-4.55545799259643	5.22715614389952e-06	0.00117653395584852
ENSG00000131069	ACSS2	2119.97372654082	-0.7215051106016	0.225500231231696	-3.19957592353984	0.00137629931978282	0.0423003241466701
ENSG00000131203	IDO1	285.229772731854	2.56175323920895	0.555429361171157	4.61220349210085	3.98422654145363e-06	0.00102620531867709
ENSG00000131401	NAPSB	46.9962741425642	1.29911616776267	0.388858983537527	3.34084134033463	0.000835249389528596	0.0295998166638049
ENSG00000131446	MGAT1	2363.42870716722	0.562015264399998	0.166000914051482	3.38561548056115	0.000710188208261616	0.0261701212318705
ENSG00000131730	CKMT2	190.58995668599	-3.44355612972424	0.748535493520713	-4.60039124334317	4.216981459657e-06	0.00106421277563708
ENSG00000132514	CLEC10A	34.764474762756	2.83719343220312	0.46141795501096	6.14885788771641	7.80428635826058e-10	3.16705823601175e-06
ENSG00000132825	PPP1R3D	298.401831666788	-0.884627903127979	0.242060099938605	-3.65457959966286	0.000257603734047515	0.0134082743571732
ENSG00000132965	ALOX5AP	177.549743173768	1.11883435625579	0.346380866749711	3.23006974015178	0.00123760025611403	0.0390406626246881
ENSG00000133055	MYBPH	2.10611387135794	2.81293567952913	0.722660367535962	3.89247259970868	9.92277094742839e-05	0.00692870403786526
ENSG00000133246	PRAM1	26.4620036563387	1.33361863616709	0.405222619659551	3.291076488493	0.000998047720786647	0.0330764457314609
ENSG00000133561	GIMAP6	156.039082384429	1.09792270541371	0.302748542524863	3.62651689833833	0.000287270028049242	0.0143256574466712
ENSG00000133574	GIMAP4	169.343825347576	1.10895448127559	0.276146185289564	4.01582401043399	5.92384504828868e-05	0.00501699473513371
ENSG00000133739	LRRCC1	514.318238376947	-0.709020004973408	0.207917627593183	-3.41010049595552	0.000649389460837471	0.0244893670314956
ENSG00000133805	AMPD3	243.523818449295	0.59863177899919	0.191188949163121	3.13110031526164	0.00174152661550654	0.048560603752026
ENSG00000134049	IER3IP1	1068.66933166176	0.781290663087724	0.20259044259843	3.85650306631877	0.000115020655911243	0.00778774001974659
ENSG00000134258	VTCN1	10.9219369121094	3.50926373644012	0.928339230796675	3.78015236243821	0.00015673241731658	0.00974080277173492
ENSG00000134265	NAPG	702.487508643345	0.658996959902065	0.151859914094188	4.33950568082986	1.42803575501771e-05	0.00210168826764897
ENSG00000134291	TMEM106C	2408.86935592241	-0.58412984464817	0.167652461530299	-3.48417100063038	0.000493663973225478	0.0209400691121653
ENSG00000134339	SAA2	128.714600224533	2.80497778828743	0.613650404651624	4.57097032288254	4.85471037817733e-06	0.00112649701767252
ENSG00000134460	IL2RA	77.6671652044741	1.41611867645624	0.352817018240996	4.01374821293042	5.97620952752654e-05	0.00502725989345869
ENSG00000134470	IL15RA	385.680634231816	0.696796045672953	0.193269037819042	3.60531647249863	0.000311772758834703	0.0152731972680906
ENSG00000134516	DOCK2	129.091607446031	1.27160441104079	0.314004722762744	4.04963466744284	5.12976517673621e-05	0.00463300480097051
ENSG00000134575	ACP2	690.886725462205	0.475003053812249	0.139107407210412	3.41464961023798	0.000638641093108543	0.0243228796802193
ENSG00000134594	RAB33A	9.38346154681117	1.09265153005584	0.294514428825216	3.71001018325078	0.000207250910882551	0.0115837958780529
ENSG00000134668	SPOCD1	58.6300102232025	1.22375201968281	0.388312883719116	3.15145871020856	0.00162457130235948	0.0462808317196684
ENSG00000134954	ETS1	1163.54349144475	0.832290921943779	0.223003522653274	3.73218733068098	0.000189824245063373	0.0109850117153476
ENSG00000135046	ANXA1	1890.52602714939	1.50626289562147	0.362555503911898	4.15457186380903	3.25897181554904e-05	0.00362475419726277
ENSG00000135047	CTSL	996.685337398703	1.10958725267698	0.271134513673564	4.09238660782554	4.26956039955054e-05	0.00422228555691326
ENSG00000135077	HAVCR2	105.890599140165	1.38397269319514	0.302024108837064	4.58232522736045	4.59833987148442e-06	0.00109414212713492
ENSG00000135093	USP30	298.277950386465	-0.580110716633177	0.166992559792464	-3.47387163448375	0.00051300639478375	0.0213615862787954
ENSG00000135220	UGT2A3	299.488953168629	-2.45887487425186	0.773264639834258	-3.17986203892485	0.00147345183651331	0.0436687078095476
ENSG00000135363	LMO2	127.086163922824	1.15766582407059	0.28206776918344	4.10421164893079	4.05695929093409e-05	0.0041202874359633
ENSG00000135604	STX11	29.7601188971117	1.45478152334465	0.317048657542674	4.58851185373286	4.46416962744283e-06	0.00109345896051011
ENSG00000135905	DOCK10	143.323529515559	1.20000436732144	0.360179097811784	3.33168797026782	0.000863209824994095	0.0302676034639377
ENSG00000136040	PLXNC1	192.566183225407	1.06146900397834	0.303848626812595	3.49341385910892	0.000476886810834072	0.0203928445450998
ENSG00000136048	DRAM1	520.889162257159	0.91360641106017	0.2564945335149	3.56189427720142	0.000368188656482173	0.0170348618399086
ENSG00000136286	MYO1G	164.668434422172	1.37795200368301	0.330629789129543	4.167658356831	3.07744712565473e-05	0.00349486086306172
ENSG00000136404	TM6SF1	23.8148514740917	1.18144815141691	0.375113437578097	3.14957565648642	0.00163507766209747	0.046368649613897
ENSG00000136490	LIMD2	321.385656703352	1.33938569613355	0.296481641324101	4.51760078685408	6.25442602823737e-06	0.00132424220245324
ENSG00000136634	IL10	11.3602996707064	1.57032973482669	0.314598665107183	4.99153336932208	5.99018311759568e-07	0.000287805259634636
ENSG00000136881	BAAT	19.0795575124704	-3.0395009410851	0.698451748089932	-4.35176939480397	1.35043259110469e-05	0.00205726877171705
ENSG00000137265	IRF4	63.9595378134197	1.97807970944007	0.428068537494459	4.62094159271322	3.82002318254275e-06	0.0010046258862384
ENSG00000137285	TUBB2B	77.8894516093404	1.52928471735718	0.397737588663837	3.84495899041042	0.00012057260125764	0.00805450767331783
ENSG00000137338	PGBD1	45.0445236206452	0.696257332722805	0.198310828900058	3.51093955173621	0.000446525922630221	0.0194355464303021
ENSG00000137473	TTC29	4.83449765599527	4.56567558197963	1.18609176290824	3.84934431277458	0.000118434431504038	0.00797045274255485
ENSG00000137496	IL18BP	180.131875169936	1.45574829647096	0.252962172861518	5.75480626215171	8.67412149689818e-09	1.8059520956542e-05
ENSG00000137672	TRPC6	32.6287103593502	1.51301866594665	0.315358842143029	4.79776833167224	1.60443193705937e-06	0.000537042885265508
ENSG00000137710	RDX	375.453631714147	1.15416780318749	0.311877493254656	3.70070886213351	0.000214998034767372	0.0119102237264479
ENSG00000137841	PLCB2	236.543914983676	0.980954071101071	0.307472411394529	3.19038077807369	0.00142085451274244	0.0427957962569227
ENSG00000138074	SLC5A6	1286.65432923492	-1.0640102798693	0.23018740967231	-4.62236523441489	3.79389279385863e-06	0.0010046258862384
ENSG00000138100	TRIM54	58.6334184132039	-5.32281541023161	0.971282903985756	-5.48019056897729	4.24867960880367e-08	4.5879821239805e-05
ENSG00000138166	DUSP5	624.515819593747	1.0612359693154	0.307607302607992	3.44997001761952	0.000560648829478297	0.0226288374082161
ENSG00000138411	HECW2	128.641982913435	0.979831472884737	0.306844598621362	3.19324986422141	0.00140681159604422	0.0425901555261309
ENSG00000138755	CXCL9	666.41229194507	1.99354740411447	0.520079148043237	3.83316157091677	0.000126506802866242	0.0083394352580744
ENSG00000138964	PARVG	128.873970279439	1.22494513505098	0.344870171617499	3.55190223992345	0.000382456927884552	0.0175326676812251
ENSG00000139209	SLC38A4	36.3630007491583	-1.85634897123091	0.586443266853464	-3.16543658381666	0.00154850412605304	0.045248920567613
ENSG00000139517	LNX2	955.88777611988	-0.747254296106133	0.21364421074808	-3.49765759385478	0.00046936331384511	0.0201487509159901
ENSG00000139572	GPR84	15.8845023854672	1.78775613863796	0.487402470446868	3.66792588679102	0.000244526035308754	0.0130741668007767
ENSG00000139626	ITGB7	144.611096686185	1.61882505642572	0.299852207423563	5.39874316863311	6.71093627887893e-08	6.20985304589303e-05
ENSG00000140030	GPR65	42.8131039442892	1.03197063248024	0.268889680114585	3.83789601758042	0.000124093005869007	0.00825622677011305
ENSG00000140093	SERPINA10	40.4472471323771	-2.59484789631736	0.616873653845083	-4.20644953815617	2.59413973895731e-05	0.00314773102583305
ENSG00000140105	WARS	3572.72734941509	1.38862904495885	0.26289400385372	5.28208717050663	1.27720398361682e-07	0.000110033325264423
ENSG00000140368	PSTPIP1	88.3465589652562	1.18523670940567	0.325967790524154	3.63605466355982	0.000276845604827358	0.0139731528830447
ENSG00000140379	BCL2A1	141.796807154197	2.08045771321167	0.429266120530034	4.84654533333036	1.25629891283498e-06	0.000475566243004078
ENSG00000140511	HAPLN3	354.641711401491	1.24984801686272	0.347594363492404	3.59570852733356	0.000323509863807844	0.0156943115288838
ENSG00000140749	IGSF6	182.497033047922	1.12095599807779	0.345386546846288	3.24551146624903	0.00117239842978644	0.0374350363080741
ENSG00000140992	PDPK1	1520.95665350913	-0.755147655848831	0.182288688804389	-4.14259195565978	3.43402608612989e-05	0.00368221921613172
ENSG00000141506	PIK3R5	90.9879191805918	1.31056397327427	0.39264612985537	3.33777382132103	0.000844524638417554	0.0298438522860314
ENSG00000141837	CACNA1A	49.6533486535946	0.94236314613013	0.296697761826529	3.17617207601689	0.00149232402417638	0.044035814498124
ENSG00000141968	VAV1	115.955927933068	1.03350564936978	0.316738729449108	3.26295950977423	0.00110255271139519	0.03568157634909
ENSG00000142149	HUNK	323.971109616046	-1.6719069735652	0.476578868135836	-3.50814332180728	0.000451245876947314	0.0195727899125897
ENSG00000142512	SIGLEC10	76.6037857693469	1.42024590569316	0.371715949576342	3.82078279748788	0.000133028770957154	0.00867778280311625
ENSG00000142677	IL22RA1	725.102888870724	-0.889651769956914	0.257254537870188	-3.45825491484949	0.000543686691250124	0.0221933492328264
ENSG00000143119	CD53	277.133393462077	1.39813120335527	0.316673446133528	4.41505664723696	1.0098354297594e-05	0.00177674143500766
ENSG00000143158	MPC2	2144.76524703302	-0.668649658681377	0.18148670085921	-3.68429011886711	0.000229340907656243	0.0124857376831952
ENSG00000143226	FCGR2A	411.379092045744	1.2424577829196	0.304225236756679	4.08400629798284	4.42658301803808e-05	0.00425360577394859
ENSG00000143258	USP21	400.588781042514	-0.528661722392481	0.139307067659373	-3.79493827036212	0.000147680194994056	0.00934086580185186
ENSG00000143315	PIGM	445.399877105236	-0.566669291275189	0.171814589041057	-3.29814420555274	0.000973261366037879	0.0328150633860869
ENSG00000143382	ADAMTSL4	214.657162166519	2.09666427894045	0.370316177818062	5.66182199031702	1.4977415014266e-08	2.20115139244954e-05
ENSG00000143546	S100A8	191.321529241046	1.65875398513738	0.503769877692717	3.29268195378122	0.000992366624665227	0.0330136987358669
ENSG00000143556	S100A7	3.79736179673215	3.99854364388257	0.97995688980614	4.08021688043192	4.49937008571252e-05	0.00427423050271641
ENSG00000143603	KCNN3	56.4775297335036	1.70520204936483	0.333066851237704	5.119699072508	3.0602358027337e-07	0.000200067637467126
ENSG00000143627	PKLR	38.358816731414	-4.00418187538307	0.731530762762776	-5.47370265094588	4.40728350046158e-08	4.5879821239805e-05
ENSG00000143702	CEP170	429.249981456163	0.947737293626434	0.235152975581099	4.03030108926658	5.57054517271048e-05	0.0047900244472215
ENSG00000144354	CDCA7	2568.14179500797	-0.779074129363462	0.227730802090078	-3.42103098137469	0.000623842251731005	0.0240254594200237
ENSG00000144834	TAGLN3	8.30763070793649	2.56524087657903	0.779239394066144	3.29198048265163	0.000994845155965983	0.0330521427881039
ENSG00000144837	PLA1A	50.357181961616	1.70202188058425	0.419779921089821	4.05455762668569	5.02292672332923e-05	0.0045849938298011
ENSG00000144843	ADPRH	100.543061497076	0.692483100192857	0.22174140512095	3.12293096463035	0.00179059742534366	0.0492148361658812
ENSG00000144847	IGSF11	2.3271145646873	2.64539737220752	0.750808021100514	3.52340052032206	0.000426046999761913	0.0189401392207324

ENSG00000144852	NR1I2	664.668614505302	-1.16717150260422	0.275428846952038	-4.23765163133935	2.2586987204866e-05	0.00286453445850951
ENSG00000145198	VWA5B2	17.2136137208022	1.57646151597089	0.497344021888829	3.16976066181263	0.00152564548102295	0.0447905131584928
ENSG00000145416	MARCH1	106.985062851582	0.997793993119655	0.268823073726945	3.71171261189119	0.000205861645429956	0.0115593569883648
ENSG00000145632	PLK2	721.66786205962	1.21651939305149	0.346192029752677	3.51400173458812	0.000441409930433935	0.0193816971914964
ENSG00000145649	GZMA	152.451845600637	1.59599502396417	0.388631372192732	4.1067066072388	4.01340479573676e-05	0.00411222139431798
ENSG00000145681	HAPLN1	67.9407699898941	1.86310793590669	0.440333713934404	4.23112775867183	2.3252316828379e-05	0.00291927579718703
ENSG00000146070	PLA2G7	95.8306274166739	1.13029461749953	0.300548898632015	3.76076779067966	0.000169392623767901	0.0101354053354664
ENSG00000146112	PPP1R18	761.36001456683	0.929943401869121	0.264842141653198	3.51131204446629	0.000445900655274234	0.0194355464303021
ENSG00000146192	FGD2	87.4472059289136	1.09186681548447	0.320001133771746	3.41207170929367	0.000644711507424579	0.0244179746381093
ENSG00000147113	DIPK2B	124.342041483905	1.0107493763977	0.315534448981396	3.20329326848649	0.00135865547111964	0.0420823418223686
ENSG00000147443	DOK2	116.474767847135	1.80087201790999	0.366862262465844	4.90885054735673	9.16117577077732e-07	0.000375217730257542
ENSG00000147488	ST18	36.9029657010024	2.74709600760401	0.601045695004733	4.57052771600398	4.8649757139263e-06	0.00112649701767252
ENSG00000147614	ATP6V0D2	12.8982652028903	1.26813198213725	0.338373048175454	3.74773342314102	0.000178439751544402	0.0105144781900598
ENSG00000149474	KAT14	388.04877842564	-0.652466625392727	0.199244108419316	-3.27470975462717	0.00105770584148884	0.0346794261729096
ENSG00000149636	DSN1	541.897133517108	-0.91913414616315	0.196552693792605	-4.67627346350687	2.92134984095875e-06	0.000829397777574015
ENSG00000150337	FCGR1A	101.797910358595	1.74422061545445	0.459163493917877	3.79869183538883	0.00014546181856205	0.00927096447692414
ENSG00000150630	VEGFC	58.224728862988	1.14041628540305	0.364104287350475	3.13211441068619	0.00173552229692695	0.0485014419087505
ENSG00000150637	CD226	57.9914496161184	1.78181066042652	0.37278536895374	4.77972262008928	1.75537206073836e-06	0.000569561241110222
ENSG00000150681	RGS18	18.3917757740765	1.18919262257125	0.320333568172329	3.71235718240901	0.000205337930850671	0.0115593569883648
ENSG00000151117	TMEM86A	60.2357472748941	0.926998320925926	0.249124434601829	3.72102528765406	0.000198415566373543	0.0113178413476635
ENSG00000151702	FLI1	140.910093578754	1.08476785176374	0.308372521255901	3.51771910832582	0.000435272840664506	0.0191962736767049
ENSG00000151743	AMN1	122.752135967215	0.776136749757514	0.189552913615432	4.09456512671788	4.22961513507561e-05	0.00422228555691326
ENSG00000152128	TMEM163	33.2892272819483	2.14133747063525	0.441123840285185	4.85427735950722	1.20826529437437e-06	0.000464420001763836
ENSG00000152207	CYSLTR2	9.54953999976848	1.59707323772751	0.422305765248131	3.78179359400677	0.000155702442858439	0.00970092227524998
ENSG00000152315	KCNK13	9.27445496994602	1.446124411158	0.344195349452328	4.20146412047409	2.65194281860542e-05	0.00317015020957119
ENSG00000152689	RASGRP3	164.307469668389	1.13706768845961	0.251507498614663	4.52100909405379	6.15455438635868e-06	0.00131535041903915
ENSG00000152782	PANK1	459.355761089422	-0.6601235456336	0.207569874101258	-3.18024736726282	0.00147149382280918	0.0436624722910505
ENSG00000153237	CCDC148	13.0233754459797	1.38426785176374	0.443785216091191	3.11922930636628	0.0018132478637973	0.0496190412147991
ENSG00000153283	CD96	105.742581288504	1.43693346268932	0.361945955541454	3.9700221557656	7.18659488009007e-05	0.00559345440760655
ENSG00000153563	CD8A	180.462694222882	1.39098053357043	0.443875567782429	3.13371727243215	0.00172607078559815	0.0483454624522243
ENSG00000153707	PTPRD	710.308895964661	-1.65687222157949	0.531587493022418	-3.11683823138709	0.00182801850870717	0.0497024505499483
ENSG00000154102	C16orf74	55.8613545842006	1.61687074999209	0.433241579830913	3.7320304081227	0.000189942565683245	0.0109850117153476
ENSG00000154451	GBP5	277.754756698464	2.15881743802595	0.439770063244585	4.9089686144127	9.15566279177902e-07	0.000375217730257542
ENSG00000154589	LY96	67.3218520689726	1.45170335394016	0.418006844482414	3.47291766415377	0.000514833269029796	0.0213664358695024
ENSG00000154930	ACSS1	1344.50046694507	-0.839669433318093	0.252738190368146	-3.32228948895696	0.000892820119675152	0.0308668517529483
ENSG00000155269	GPR78	14.2467037271578	2.43199882556801	0.670746847815005	3.62580731238675	0.000288060096830982	0.0143364411538352
ENSG00000155307	SAMSN1	133.952197523294	1.26213299941597	0.346758978283056	3.63979904908389	0.000272850891457806	0.0138837203099426
ENSG00000155659	VSIG4	110.069006926719	1.75506730182255	0.422292063419907	4.15605088006921	3.23795744587783e-05	0.00362475419726277
ENSG00000155850	SLC26A2	2039.01207822878	-2.5853435792895	0.493829668030666	-5.23529416446472	1.64722304713997e-07	0.000126717885508766
ENSG00000155926	SLA	188.308718013544	1.08909968467467	0.312005172519327	3.49064624756246	0.000481853806894452	0.0205437466065717
ENSG00000155962	CLIC2	92.1614920745493	1.23712680988692	0.266164123593876	4.64798483425429	3.35193551143121e-06	0.000920187855501581
ENSG00000156076	WIF1	47.0975603181002	-5.45048370918534	1.23787818915365	-4.40308566460154	1.06722000784999e-05	0.00183751667366846
ENSG00000156103	MMP16	30.4793754485187	1.22545282024473	0.355708788546988	3.4451013292376	0.000570844535403732	0.0229694376377272
ENSG00000156273	BACH1	863.897683219899	0.54061190275045	0.163304546949611	3.31045223693166	0.000931453472210776	0.0318360558632408
ENSG00000156414	TDRD9	6.2089414004855	1.59918295207168	0.491848876520174	3.2513705498036	0.00114850063229004	0.0368819277598128
ENSG00000156574	NODAL	5.86860374749638	-2.14822307226163	0.683841250411361	-3.14140609530264	0.00168138717635015	0.0474664149309967
ENSG00000156587	UBE2L6	1198.8942396326	0.890458598448946	0.281449603059868	3.16382964753918	0.00155707905709044	0.0453403999561159
ENSG00000157017	GHRL	11.1335356636212	2.12666724839568	0.491945370338739	4.32297441264936	1.53939593180191e-05	0.00220859202853756
ENSG00000157388	CACNA1D	332.845515083291	-0.747809109738568	0.234057274218988	-3.19498341691745	0.00139838875315835	0.0424574558316833
ENSG00000158122	PRXL2C	160.839270239884	0.683126490108203	0.198674246465058	3.43842507468073	0.000585108431959349	0.0232037286731308
ENSG00000158296	SLC13A3	87.2368634410105	-2.858975730911	0.590272741646333	-4.84348256186964	1.27582923274978e-06	0.000475751008224187
ENSG00000158428	CATIP	4.05121219376266	1.63376168003297	0.404582671970259	4.03814051668795	5.38765784224236e-05	0.00469113593938469
ENSG00000158480	SPATA2	636.831371269136	-0.65137529488489	0.177998222278405	-3.65944831665837	0.000252758812492372	0.013294581413283
ENSG00000158485	CD1B	3.08862028708533	2.07947774475779	0.586430213078644	3.57748918450838	0.000346910568821958	0.0164775924932468
ENSG00000158488	CD1E	5.47573526538685	1.58253738250576	0.484173272591923	3.26853519615813	0.00108105731078407	0.0352139972002989
ENSG00000158517	NCF1	112.0380507819	1.42838120945388	0.353727147404073	4.03808760491372	5.38887293709337e-05	0.00469113593938469
ENSG00000158683	PKD1L1	63.5119627654387	-1.82641038658641	0.522866550764029	-3.49307176798998	0.00047749816117849	0.0203928445450998
ENSG00000158714	SLAMF8	159.333272914868	1.64855200020983	0.354753220280232	4.64703885959818	3.36733751016429e-06	0.000920187855501581
ENSG00000158764	ITLN2	8.81766479240232	3.53840562665057	1.02884834084095	3.43919068164934	0.000583456115790101	0.0231749882303655
ENSG00000158869	FCER1G	265.26258168559	1.3879631447226	0.318496239259129	4.3578635275293	1.31338233864723e-05	0.00203810834464363
ENSG00000159189	C1QC	946.326491641526	1.28868601083916	0.341866965084531	3.76955407352833	0.000163539475832325	0.00996553722974346
ENSG00000159339	PADI4	7.66015258636288	2.0946445868872	0.605901292439315	3.45707232023604	0.000546078276829366	0.0221933492328264
ENSG00000159753	CARMIL2	115.391050501328	1.38308676674102	0.398894162955418	3.46730259599112	0.000525709720122452	0.0216025191571371
ENSG00000160219	GAB3	36.882496318312	1.37555324126181	0.349940734631099	3.93081772321504	8.46574376609066e-05	0.00629201579993022
ENSG00000160255	ITGB2	685.687449851159	1.31870151039638	0.367347924881606	3.58978891964993	0.000330945839452559	0.0159313118552654
ENSG00000160326	SLC2A6	96.7207820086815	0.986638103057929	0.250920625501263	3.93207254719266	8.42166404043332e-05	0.00628080162346824
ENSG00000160396	HIPK4	1.17965282666987	2.20937926133151	0.508337786659561	4.34628178213939	1.38464670050176e-05	0.00207149779433149
ENSG00000160593	JAML	122.45957658582	1.35286549235773	0.330561019200815	4.0926346839942	4.26499368965177e-05	0.00422228555691326
ENSG00000160791	CCR5	84.169612614775	1.44091576880336	0.36987030424517	3.8957325101943	9.79023912757884e-05	0.00687076781919746
ENSG00000160867	FGFR4	1730.46101926784	-0.851032907427449	0.266346015074933	-3.19521546882548	0.00139726481410027	0.0424574558316833
ENSG00000160883	HK3	112.556532833222	1.81820045259677	0.412977158646397	4.40266589696202	1.06928767856811e-05	0.00183751667366846
ENSG00000160886	LY6K	8.81267865144248	2.40251775659749	0.600262856370549	4.00244254429378	6.2691895163862e-05	0.00515228391044056
ENSG00000161267	BDH1	1178.39698452926	-0.56603767718999	0.181421314345172	-3.12001750859906	0.00180840290632017	0.0495407217231395
ENSG00000161642	ZNF385A	254.498747123818	1.31016286944396	0.384022782989208	3.41168005514083	0.000645638452824163	0.0244179746381093
ENSG00000161929	SCIMP	35.4198112372914	1.20145652028675	0.318717680135661	3.76965758465403	0.000163471667913767	0.00996553722974346
ENSG00000161944	ASGR2	4.25250131348366	1.29528505410231	0.378176360820046	3.42508202071009	0.000614613841224058	0.0238810454263482
ENSG00000162460	TMEM82	34.2345689980551	-2.11986827966495	0.549193503376893	-3.85996605318572	0.000113402777968243	0.0077200408849008
ENSG00000162511	LAPTM5	1316.21080769543	1.13276136634069	0.278788572154184	4.06315566519786	4.84137197757213e-05	0.00447752254653854
ENSG00000162618	ADGRL4	226.863435304919	0.977426195902049	0.301829761026011	3.23833604936597	0.00120229101203616	0.0381752289520448
ENSG00000162620	LRRIQ3	13.2058466130555	-1.28721916384205	0.375498588393323	-3.42802663879451	0.000607985797836935	0.0237342455830594
ENSG00000162654	GBP4	696.651349802585	1.56740677250524	0.364266012008539	4.30291797981005	1.68563264052392e-05	0.00237931332716665
ENSG00000162946	DISC1	71.1803354362913	1.13374959230286	0.256585959170361	4.41859560814902	9.93443315095606e-06	0.00177674143500766
ENSG00000163050	COQ8A	1278.55381305258	-0.667096690886735	0.179167767426033	-3.72330870921162	0.000196622852445936	0.0112415909599754
ENSG00000163075	CFAP221	2.90134619800003	2.63572513090665	0.834842523361678	3.15715246546537	0.00159318021062653	0.0458043893927425
ENSG00000163121	NEURL3	35.3428644286913	1.23986809397184	0.376493419119856	3.29319991002851	0.000990540183160442	0.0330136987358669
ENSG00000163154	TNFAIP8L2	33.267610469911	1.33272802193322	0.323515990746243	4.11951204903061	3.79675571306219e-05	0.00398563633340949
ENSG00000163216	SPRR2D	24.1020309246552	6.23574727540019	1.15355859711271	5.40566148179026	6.45696465252325e-08	6.20464634148619e-05
ENSG00000163219	ARHGAP25	85.5191421719612	0.904152638930509	0.215436390160411	4.19684268872725	2.70661794155026e-05	0.00320484088396643
ENSG00000163220	S100A9	786.237538774838	1.99571753965206	0.419282398163225	4.75984097685669	1.93745550391903e-06	0.000606919835545906
ENSG00000163421	PROK2	17.9762061877846	2.99879389419991	0.625447134555945	4.79464007190471	1.62967162489515e-06	0.000537042885265508
ENSG00000163508	EOMES	17.4820281056536	1.64637330347994	0.468739724572379	3.51234004112174	0.000444179294266481	0.0194349833414251
ENSG00000163536	SERPINI1	128.512736891845	2.35450270676851	0.428382017734353	5.4962687724875	3.87911147870519e-08	4.40526005381684e-05
ENSG00000163563	MNDA	77.5972404002405	1.40289956729465	0.391824360783733	3.58042956984194	0.000342932968019721	0.0163554456680532
ENSG00000163564	PYHIN1	17.4198047786852	1.04874376339591	0.314396952705626	3.33573132427228	0.000850753414914981	0.0299791584178221
ENSG00000163565	IFI16	916.284179353792	1.10410873497384	0.331961037523349	3.32601905094414	0.000880959071806734	0.0305692797916937
ENSG00000163599	CTLA4	41.4824044609635	1.54537490747687	0.380752893729849	4.05873450451921	4.93393701423697e-05	0.00453196626337119
ENSG00000163638	ADAMTS9	228.527801858327	0.867867274854925	0.260610358666164	3.33013345784402	0.000868043677610696	0.0302676034639377
ENSG00000163661	PTX3	22.0846007986276	1.70586510910814	0.453579429300184	3.76089610531958	0.000169305744377739	0.0101354053354664

ENSG00000163808	KIF15	364.31252762677	-0.733761590691521	0.200001141549772	-3.66878701294271	0.000243703997984284	0.0130658812996552
ENSG00000163823	CCR1	93.002020078208	1.10663012679433	0.313159585070022	3.53375780130403	0.000409696321023185	0.0184762687444824
ENSG00000163874	ZC3H12A	1073.58180108101	0.841934432269383	0.267018450698601	3.1530945897807	0.0016154945041703	0.0461274453625036
ENSG00000164047	CAMP	1.90823631557395	2.79871898494695	0.681525348102219	4.10655156516231	4.01609838381999e-05	0.00411222139431798
ENSG00000164082	GRM2	9.12701070116833	2.01345724666366	0.480135716827753	4.19351690802453	2.74662594164559e-05	0.00323687276066384
ENSG00000164543	STK17A	460.415776481219	0.572345080329598	0.177433226613812	3.22569279301515	0.001256681659005	0.0395926034912746
ENSG00000164674	SYTL3	50.4040025127178	0.778331809883347	0.216418347702344	3.59642247594388	0.000322623673591125	0.0156919520668221
ENSG00000164683	HEY1	95.5515987752152	1.1630439785595	0.275493008351304	4.22168237778435	2.42485641570412e-05	0.00299759747580725
ENSG00000165046	LETM2	77.4594196196562	1.80246324017131	0.454109525229992	3.96922579251871	7.21065311623881e-05	0.00559475023155622
ENSG00000165168	CYBB	388.910118023314	1.45308975925703	0.301463515214252	4.82011814339892	1.43473230120713e-06	0.000499900661140903
ENSG00000165178	NCF1C	64.5632486738736	1.57319136405649	0.359804245159776	4.3723535372905	1.22914272933976e-05	0.0019559810159124
ENSG00000165556	CDX2	3619.56322648428	-1.05153303674639	0.332722520097024	-3.16039033498471	0.00157557904679942	0.0455604941032831
ENSG00000165794	SLC39A2	47.7115709360776	-5.27821433609248	1.21563571509328	-4.34193753157989	1.41231692951752e-05	0.00210031703375392
ENSG00000166130	IKBIP	276.220190392702	0.744679758047956	0.235913752800731	3.15657628776295	0.00159633123799842	0.0458422294829341
ENSG00000166165	CKB	8921.23640866134	-1.64666541554206	0.511154115181719	-3.2214656336214	0.00127536756735644	0.0400298785211473
ENSG00000166527	CLEC4D	4.6325942410957	1.91492160365395	0.570252421895291	3.35802449955323	0.000785016550156491	0.0280183621272997
ENSG00000166546	BEAN1	29.6193371192074	1.93471336820753	0.548560082925195	3.52689418794506	0.000420464761139913	0.0188597694655648
ENSG00000166619	BLCAP	1850.63034571975	-0.617194027331472	0.138400025179229	-4.45949360581548	8.21535240966461e-06	0.00157886434310047
ENSG00000166927	MS4A7	119.405289425568	1.12199696549024	0.319396231262918	3.51286851774605	0.000443296785943051	0.0194303980701775
ENSG00000167077	MEI1	40.297785186577	1.13134863583271	0.351005920177868	3.22316112292185	0.00126784216206611	0.0398437340591946
ENSG00000167080	B4GALNT2	187.384976796232	-1.56454649253913	0.401212856738105	-3.89954226606551	9.63747131537635e-05	0.00682518002493358
ENSG00000167083	GNGT2	13.3542497280529	1.30470339385839	0.322062918527914	4.05108231590873	5.09812670012132e-05	0.00463169445366658
ENSG00000167220	HDHD2	508.062328297478	0.823579952934442	0.221177353113742	3.7236179081631	0.000196388078286192	0.0112415909599754
ENSG00000167286	CD3D	99.5317520188386	1.32015327988007	0.407507014004111	3.23958418999569	0.00119704122158562	0.0380979336052166
ENSG00000167434	CA4	169.150377566078	-3.31247209852224	0.832813191498427	-3.97744912344907	6.96585206388348e-05	0.00554251108165812
ENSG00000167600	CYP2S1	2421.9382672646	-1.25915320553216	0.24242033478084	-5.19409069651892	2.05722837840257e-07	0.000151169981782382
ENSG00000167613	LAIR1	177.514408208231	1.34737512166703	0.306703294675574	4.39308982021946	1.11750929257207e-05	0.00186413292008799
ENSG00000167619	TMEM145	17.518526194487	1.43446131921582	0.442970085484286	3.23828034041523	0.00120252582393769	0.0381752289520448
ENSG00000167634	NLRP7	1.77206863447644	2.87704655497512	0.894969189670957	3.21468782185998	0.00130586453394959	0.0408843602959855
ENSG00000167779	IGFBP6	227.16136257719	1.49289675159987	0.396253725814157	3.76752735518767	0.000164872479152527	0.00999799519210373
ENSG00000167851	CD300A	108.94205697704	0.993107501666444	0.31584058609846	3.14433149309334	0.00166466751369354	0.0470475714503612
ENSG00000167895	TMC8	329.302244601287	1.19683645483278	0.297018711322359	4.02949851039463	5.58959700279853e-05	0.0047900244472215
ENSG00000167964	RAB26	177.637007365407	1.75755051264642	0.486430405795718	3.6131592345083	0.000302488810934952	0.0149355344909068
ENSG00000167984	NLRC3	79.6684296651852	1.05679004868037	0.274359205631073	3.85184833236986	0.000117229625215256	0.00791585123345391
ENSG00000168267	PTF1A	10.8187971917854	-4.66852258165297	1.36680091370932	-3.41565661452712	0.000636284283462834	0.0242701168519625
ENSG00000168394	TAP1	4531.73648928429	0.830611967025	0.25946746638559	3.20121816656059	0.00136847875054129	0.0421641735901594
ENSG00000168421	RHOH	66.7244764309614	1.17152660011154	0.342496241338137	3.42055315858174	0.000624939208194737	0.0240254594200237
ENSG00000168612	ZSWIM1	291.564335617833	-0.568228240483013	0.179883234323525	-3.15887271329044	0.00158380647552559	0.0456399319314087
ENSG00000168899	VAMP5	336.059390471683	1.33740014144465	0.324628274771162	4.11978944960173	3.7921876999954e-05	0.00398563633340949
ENSG00000168903	BTNL3	212.763840099061	-1.60659239629363	0.46915330447724	-3.424450773258	0.000616043440720786	0.0238994244145468
ENSG00000168995	SIGLEC7	14.2983410963492	1.45821344572219	0.388625894598227	3.75222924151706	0.0001752690824203	0.0103765942066085
ENSG00000169035	KLK7	289.273351210798	2.92322954206772	0.730278172171598	4.00289869458241	6.25710995050018e-05	0.00515228391044056
ENSG00000169136	ATF5	694.758782377984	0.851837504328373	0.217385089706312	3.91856454129034	8.9077787545413e-05	0.00648719576467403
ENSG00000169245	CXCL10	580.156443965999	1.91425335232618	0.489475106400897	3.91082881906223	9.19799461372287e-05	0.0065915977852098
ENSG00000169248	CXCL11	512.787518716046	2.04685464127835	0.536668863253004	3.81399924875721	0.000136735883244421	0.00887327092721715
ENSG00000169429	CXCL8	4603.70841802203	2.3195918197052	0.529677915633303	4.37924963688888	1.19088650468675e-05	0.00193381050183383
ENSG00000169442	CD52	223.250298852607	1.27635011390972	0.376430594234753	3.39066519421572	0.00069723220662948	0.0258835801640876
ENSG00000169469	SPRR1B	39.4916177097019	4.40309737791444	1.08933007158749	4.04202315970013	5.29919972244969e-05	0.00467325979495102
ENSG00000169495	HTRA4	6.51361588839785	1.53967990720265	0.384131567202482	4.00820978711979	6.11807616411402e-05	0.00508149103813216
ENSG00000169507	SLC38A11	60.9102417837185	2.01041033441854	0.585419242267934	3.43413777557113	0.000594441969905266	0.0234251390790428
ENSG00000170011	MYRIP	111.410696570012	-1.40143206558398	0.420857396503078	-3.32994519575642	0.000868630791052006	0.0302676034639377
ENSG00000170088	TMEM192	689.008821386985	-0.677287487093469	0.19515260553862	-3.47055313570711	0.000519387568709984	0.0214343506372852
ENSG00000170099	SERPINA6	38.2068626321532	-3.02802721745621	0.896461110803058	-3.37775635882707	0.00073079799021551	0.0265381642260818
ENSG00000170175	CHRNB1	159.67945884218	0.860624830037718	0.238279899966441	3.6118230289627	0.000304052038520716	0.0149831087384646
ENSG00000170191	NANP	213.480318501806	-0.682530184009606	0.20668790757494	-3.30222600837031	0.000959207483698387	0.0324726826188625
ENSG00000170298	LGALS9B	76.839918224967	-2.05054600037859	0.590549944421675	-3.47226516528874	0.000516086309127188	0.0213829193154787
ENSG00000170442	KRT86	25.0291009922316	2.06845429483429	0.464291270579109	4.45507901161766	8.38623040470573e-06	0.00158728470023612
ENSG00000170458	CD14	560.628193029882	1.07920022546683	0.290779538941372	3.71140359254927	0.000206113168841848	0.0115593569883648
ENSG00000170471	RALGAPB	1370.4530793565	-0.557392237924703	0.146527423460966	-3.80401309706498	0.000142370647692203	0.00912126828707127
ENSG00000170476	MZB1	265.624186369975	1.83133527018927	0.51945031558839	3.52552537794665	0.000422643680070467	0.0189235299334777
ENSG00000170542	SERPINB9	457.727210140721	1.06720398437152	0.227637870744084	4.68816537812065	2.75665210974838e-06	0.000800475337508591
ENSG00000170835	CEL	428.012115733056	-2.44198202147075	0.670207008109248	-3.64362352515521	0.000268826545780332	0.0137348924739792
ENSG00000170891	CYTL1	11.0491648934352	1.28686205096871	0.387930829765397	3.31724614861611	0.000909094856048527	0.0312418512840666
ENSG00000171115	GIMAP8	81.3919246344622	1.05360453152395	0.279816953491269	3.76533480030957	0.000166326063559261	0.0100617200289699
ENSG00000171310	CHST11	269.752927025374	1.26495456064723	0.392326928491439	3.22423588284187	0.00126309310699402	0.0397444813414843
ENSG00000171365	CLCN5	476.014993290244	-0.81993070470172	0.246935636472248	-3.32042274827299	0.000898812304613494	0.0309736918875359
ENSG00000171488	LRRC8C	125.361368153908	0.955786712086909	0.218403901877649	4.37623459961053	1.20747053428625e-05	0.00193381050183383
ENSG00000171608	PIK3CD	162.820579337354	1.33712697123435	0.318981601967372	4.19186236130045	2.76673840933101e-05	0.00324526724970544
ENSG00000171631	P2RY6	57.072180327913	1.26398192333628	0.331340117256036	3.81475667300366	0.000136317185969873	0.00886913691216485
ENSG00000171700	RGS19	170.674071623079	1.21860165356355	0.301042209418296	4.04794283139981	5.16697620345735e-05	0.00463300480097051
ENSG00000171759	PAH	34.3204637141022	-2.47467716305429	0.723770730743626	-3.41914512142779	0.000628182138657351	0.0240713229328455
ENSG00000171860	C3AR1	90.1949669431594	1.17073204394152	0.333383143797123	3.51167137789382	0.000445298251649634	0.0194355464303021
ENSG00000171889	MIR31HG	6.09294468187241	2.43377156324142	0.779877622921204	3.12070957251625	0.00180415870852151	0.0494940662776087
ENSG00000171954	CYP4F22	2.20161256125739	1.85093552452305	0.540939146155655	3.42170748350765	0.000622292242727378	0.0240254594200237
ENSG00000172016	REG3A	1939.09592129335	4.87469611111457	1.0592125650215	4.60218871272136	4.18074133341782e-06	0.00106421277563708
ENSG00000172175	MALT1	538.395279170251	0.599588554918298	0.164829220499351	3.63763508133959	0.000275152892653095	0.0139440565315313
ENSG00000172243	CLEC7A	107.164532421936	1.35351350538439	0.362273265072973	3.73616724135509	0.000186846424010111	0.0108562117615549
ENSG00000172315	TP53RK	683.888203722326	-0.803050957703782	0.229147486561019	-3.50451567135099	0.000457438644154153	0.0197461965251802
ENSG00000172322	CLEC12A	21.8147356074834	1.84404128305175	0.462099864093165	3.99056876304333	6.59150346165989e-05	0.00534682215863996
ENSG00000172349	IL16	112.616385242149	1.14511425459181	0.332824879524583	3.44059090843064	0.000580445409555859	0.0231085235678311
ENSG00000172456	FGGY	500.460266301037	-1.74131905890955	0.439275570647438	-3.96406988065158	7.36826678344424e-05	0.00568175238634478
ENSG00000172543	CTSW	52.2523286191892	1.52657991048254	0.447221401391814	3.413476872376	0.000641396022181051	0.0243590827217931
ENSG00000172575	RASGRP1	58.6622592562472	1.09178000275911	0.340313404211071	3.20816044636892	0.00133586950057471	0.041615166586482
ENSG00000172840	PDP2	372.864288497623	-0.552110678902613	0.177140018536388	-3.11680377739826	0.00182823215079261	0.0497024505499483
ENSG00000172889	EGFL7	427.601453756462	1.32529226535593	0.301248050018146	4.39933890120152	1.08581147001927e-05	0.00184543631067765
ENSG00000172943	PHF8	583.358841315682	-0.391153873901319	0.125573469336132	-3.1149404087442	0.00183982074886625	0.0498547522664581
ENSG00000173369	C1QB	619.798034542285	1.40497803355679	0.338664897012569	4.14857886350307	3.34545600196387e-05	0.00366591547162568
ENSG00000173372	C1QA	974.135866256296	1.55942711317547	0.34006465074084	4.58567836962242	4.52514757093989e-06	0.00109414212713492
ENSG00000173406	DAB1	26.1040956283835	-1.80115421719855	0.552453649661116	-3.26028114449675	0.00111301827138643	0.0359271944345201
ENSG00000173432	SAA1	487.570862863499	2.69204279713947	0.607953795405143	4.42803847510398	9.50939412381714e-06	0.0017469316381577
ENSG00000173762	CD7	173.565349541465	1.46044030254267	0.364271113968296	4.00921249734278	6.09215761771389e-05	0.00508149103813216
ENSG00000174015	SPERT	10.8512891499117	-1.77448015152999	0.542175711188906	-3.27288758037285	0.00106454809300424	0.0348580203874416
ENSG00000174564	IL20RB	25.7724411071868	1.28843240000271	0.333147520786034	3.86745306392422	0.000109977987480111	0.00761132974848503
ENSG00000174600	CMKLR1	85.0679485909391	1.57895169663385	0.312994931686283	5.04465579722691	4.54338504451984e-07	0.000246765069461487
ENSG00000174837	ADGRE1	6.66067060112903	1.5368903955324	0.448235606427556	3.42875571126854	0.000606355033205696	0.0237076277771692
ENSG00000174992	ZG16	431.92575261703	-3.01538605629331	0.738286588712852	-4.08430290133052	4.42093324592506e-05	0.00425360577394859
ENSG00000175040	CHST2	102.522358091161	0.981919378661421	0.275938793204625	3.55846804741684	0.000373024159455456	0.017207531024209
ENSG00000175463	TBC1D10C	134.64428263278	1.52520621954737	0.384575617426624	3.9659462286071	7.3105343485207e-05	0.00565468700196412

ENSG00000175489	LRRC25	57.9347750291937	1.35023518344319	0.304720210820987	4.43106540194803	9.37686413003706e-06	0.00173534498833219
ENSG00000175779	C15orf53	21.7382263616008	3.68683518315638	0.583216187199842	6.32155839305103	2.58938491910843e-10	1.61732982047512e-06
ENSG00000175832	ETV4	2034.72970020104	-1.12114538350473	0.285883892098602	-3.92168084488666	8.79334146311894e-05	0.00646155420925187
ENSG00000175874	CREG2	42.4512443431556	1.68125570577748	0.488404799926806	3.44234066911185	0.000576703481704106	0.0230533756590326
ENSG00000176049	JAKMIP2	13.0604135568369	1.1169311981471	0.350286688458718	3.1886201644192	0.00142953580937062	0.0428757775045804
ENSG00000176083	ZNF683	22.3259840599369	1.95728002098682	0.614803182934357	3.18358797689537	0.00145461902468127	0.0433160926253122
ENSG00000176532	PRR15	2264.58812443353	-0.913399328304535	0.268577770008192	-3.40087464527192	0.000671706224134256	0.0251955480076645
ENSG00000176563	CNTD1	71.6796869431487	-1.31573398585366	0.377016351108507	-3.48985921163665	0.000483275076803555	0.0205692410883476
ENSG00000176809	LRRC37A3	174.628729672016	-0.805752165550933	0.244571663264141	-3.29454424440295	0.000985814240531899	0.0330136987358669
ENSG00000176956	LY6H	13.4454651569805	1.7128485090064	0.529114922712999	3.23719561758699	0.00120710635652786	0.038237513052727
ENSG00000177182	CLVS1	6.98016023347428	2.01070791941767	0.456797067343982	4.40175312663194	1.07379696748157e-05	0.00183751667366846
ENSG00000177359	AC024940.1	28.9468957026003	1.73639932837166	0.483047316913191	3.59467751413617	0.00032479363248605	0.0157260544845571
ENSG00000177494	ZBED2	27.1877124215968	2.44353971430345	0.55609185940305	4.39412962622132	1.11217441521565e-05	0.00186413292008799
ENSG00000177675	CD163L1	107.35554779236	1.42502562167832	0.445691599635051	3.1973356079522	0.00138703446498356	0.0424574558316833
ENSG00000177728	TMEM94	1320.51759292487	-0.64541877268722	0.196558257788173	-3.28360039384749	0.00102490133179487	0.0338705487745542
ENSG00000178093	TSSK6	35.4040598725348	-0.709284244074409	0.226005628553946	-3.13834769785438	0.0016990320445087	0.0477572664603769
ENSG00000178171	AMER3	4.87328137853893	3.61217972956026	1.03814517050423	3.47945531336992	0.000502434154432769	0.021167235897171
ENSG00000178460	MCMDC2	33.7919002745838	-1.63951774378022	0.448390577195859	-3.65645003968062	0.000255732224898634	0.0133854642936103
ENSG00000178934	LGALS7B	15.5905869642934	2.04314464592308	0.572370288261027	3.56962038006996	0.000357498910334826	0.0167349680380091
ENSG00000179044	EXOC3L1	106.195933777153	1.13087868152184	0.25942944571793	4.35909917007423	1.30598924304934e-05	0.00203810834464363
ENSG00000179144	GIMAP7	88.6769800807554	0.996973525760091	0.313479969643122	3.1803420387436	0.00147101312465632	0.0436624722910505
ENSG00000179344	HLA-DQB1	1366.83256958197	1.6031308424408	0.33264814640779	4.81929888908967	1.44063591106888e-06	0.000499900661140903
ENSG00000179583	CIITA	553.181924906398	1.52013676856022	0.418381297079204	3.63337648975365	0.000279736381621598	0.0140905922549073
ENSG00000179673	RPRML	14.8997156676082	-3.78805473027906	1.18284541238599	-3.20249348783282	0.00136243380600231	0.0420823418223686
ENSG00000180061	TMEM150B	196.784343131735	-1.42074324969754	0.324649321136092	-4.37623970604876	1.2074422611969e-05	0.00193381050183383
ENSG00000180096	01-sep	180.407580400536	1.2353101397638	0.321245142991405	3.84538153094145	0.000120365007675608	0.00805450767331783
ENSG00000180353	HCLS1	333.420879416339	1.18337127781472	0.292552994060593	4.04498091572982	5.23273866154762e-05	0.00463598378440091
ENSG00000180720	CHRM4	3.69201705979607	1.67065523907196	0.514831317215797	3.24505363835837	0.00117428502293834	0.0374350363080741
ENSG00000180785	OR51E1	58.4019656608571	1.39082256282267	0.421480927497278	3.29984697310331	0.000967375616660779	0.0327000997942811
ENSG00000181036	FCRL6	7.16213437848542	2.03484385355943	0.492076971070076	4.13521455623992	3.54623156299141e-05	0.00378628416110159
ENSG00000181264	TMEM136	42.8471984605719	0.874950421039007	0.269431057097836	3.24740002308381	0.00116464573225889	0.0373044986855848
ENSG00000181631	P2RY13	24.2668830829594	1.55321889800261	0.388900580273433	3.99387138201376	6.50031338081671e-05	0.00529002701974999
ENSG00000181778	TMEM252	15.9300366636992	-3.25776760309958	0.791546607074694	-4.11569903020526	3.86007736387653e-05	0.00400166692361374
ENSG00000182022	CHST15	230.286111401349	1.36781134435659	0.320170540310445	4.27213366673375	1.93611456849621e-05	0.00257297267975049
ENSG00000182162	P2RY8	53.4700569783239	1.12944715601597	0.309286617043151	3.65178153136315	0.000260427391794061	0.0134989999099229
ENSG00000182247	UBE2E2	130.497270995156	1.22137716101686	0.370839398453757	3.29354746585579	0.00098931636100116	0.0330136987358669
ENSG00000182287	AP1S2	413.662077755245	0.811281299921755	0.242386173200842	3.34706096972589	0.00081673244939822	0.0290672984555059
ENSG00000182487	NCF1B	31.5364232102644	1.48789254876483	0.41454546744768	3.5892143699596	0.000331676018407245	0.0159357570074742
ENSG00000182566	CLEC4G	2.72897285818274	2.69883607923828	0.776201151273601	3.47698025802976	0.000507095154501354	0.0212215499833531
ENSG00000182578	CSF1R	241.349787571201	1.32920852317883	0.305426253094149	4.35197861910416	1.34914421568127e-05	0.00205726877171705
ENSG00000182782	HCAR2	78.6322144929095	3.47175213908496	0.56904382676529	6.10102768150569	1.05388652773798e-09	3.29128762612572e-06
ENSG00000182985	CADM1	114.806813549283	1.32790507422099	0.392473815687691	3.38342335499309	0.000715881860017691	0.0263023417510029
ENSG00000183044	ABAT	349.97303543845	-1.1479563027664	0.36295841646056	-3.16277636970333	0.00156272325687213	0.0454284001785879
ENSG00000183307	TMEM121B	11.1224305663619	1.84285756123967	0.328023981079326	5.61805742121646	1.93116326155677e-08	2.6804546070408e-05
ENSG00000183484	GPR132	46.828154566648	1.10957455407711	0.300229895373935	3.69574972770495	0.00021923886123838	0.0121182825424329
ENSG00000183696	UPP1	513.53255860019	0.786329092807591	0.21471486988686	3.66220138000658	0.000250057162230835	0.0131802281459392
ENSG00000183760	ACP7	3.79124699027545	5.10331451272627	1.14081154872324	4.473407127091	7.69830026588259e-06	0.00153867467074249
ENSG00000183779	ZNF703	5110.48109722058	-0.755759370724218	0.236574728469644	-3.19459046032972	0.00140029393232897	0.0424574558316833
ENSG00000183844	FAM3B	330.106356006641	-2.86264212112127	0.666017960165149	-4.29814553411057	1.72233066472841e-05	0.00241745558020082
ENSG00000183856	IQGAP3	1013.04230104995	-0.773791821469443	0.216351694127028	-3.57654616291159	0.000348163856887267	0.0164793224025849
ENSG00000183918	SH2D1A	32.4440426934373	1.53324728342251	0.425470274134648	3.60365312604022	0.000313775749680895	0.0153412393934002
ENSG00000184060	ADAP2	97.2075666480245	1.01690128997764	0.284075031974191	3.57969260061555	0.000343998630764719	0.0163704034114776
ENSG00000184182	UBE2F	722.754915408343	0.452996150099975	0.12164734252127	3.72384748167245	0.000196209487934809	0.0112415909599754
ENSG00000184371	CSF1	353.52808113435	1.09440183205273	0.255060405006008	4.29075548604633	1.78066293432445e-05	0.00245973610713429
ENSG00000184557	SOCS3	1396.47199636765	1.20021158227832	0.304609697202153	3.94016209366378	8.14265713868807e-05	0.0061501516760933985
ENSG00000184634	MED12	694.708742729189	-0.561744364649304	0.16980994750091	-3.30807689959561	0.00093939011003738	0.0320450642251265
ENSG00000184922	FMNL1	330.166559607598	1.10408461584325	0.302490059469347	3.64996644180282	0.000262254151153025	0.0135494479178965
ENSG00000185105	MYADML2	46.4721482303947	-2.08526212265752	0.559388702883827	-3.72775158294641	0.000193195667145517	0.0111473453763593
ENSG00000185176	AQP12B	33.408814155563	-1.6426870800573	0.520877943500271	-3.15368907544545	0.001612207536836	0.0461252899488063
ENSG00000185215	TNFAIP2	1196.67337248368	1.19478347594904	0.337639084494887	3.53864090626963	0.00040219256496693	0.018170667347439
ENSG00000185291	IL3RA	124.943854911853	0.985629180250083	0.293818560243385	3.35455255345918	0.000794934517705474	0.028331874451289
ENSG00000185338	SOCS1	254.142417015063	0.952941913173212	0.273287685774688	3.48695518596789	0.000488553214860035	0.0207585263946651
ENSG00000185386	MAPK11	125.183958085614	1.18579595212997	0.290460852068014	4.08246393167059	4.45607295153908e-05	0.00426553741843879
ENSG00000185479	KRT6B	383.558910140679	2.57744288111664	0.687946558207069	3.74657428017954	0.000179265937476139	0.010538306310362
ENSG00000185669	SNAI3	24.5913875569223	1.65533749453066	0.391912569242736	4.22374178437081	2.40279332070117e-05	0.00298663623504467
ENSG00000185880	TRIM69	556.51725309034	0.764547600180966	0.240003050282176	3.18557451366585	0.00144466895377335	0.0430711326265791
ENSG00000186074	CD300LF	48.9900872724872	1.35445539013815	0.369459059448634	3.66605001420044	0.000246325770196666	0.0130932240415779
ENSG00000186198	SLC51B	121.488814871431	-3.3725357338176	0.57316178481853	-5.88409036182582	4.00249133146969e-09	9.99982434254387e-06
ENSG00000186469	GNG2	178.383177824371	0.910750277923369	0.229994821496362	3.95987297452162	7.49896405181885e-05	0.00576474208832745
ENSG00000186517	ARHGAP30	200.493028166149	1.20217072853336	0.297189503576311	4.04513185717098	5.22936826563857e-05	0.00463598378440091
ENSG00000186603	HPDL	366.708474120115	-1.44967005752378	0.363466153101475	-3.98845957224262	6.65037334241456e-05	0.00535977185764146
ENSG00000186638	KIF24	131.444805150393	-0.645160331036917	0.176477339587725	-3.65576867187348	0.000256412491412675	0.0133854642936103
ENSG00000186818	LILRB4	184.171895641557	1.93410942802412	0.409205351415748	4.72650081757873	2.28421872786083e-06	0.000687577357793675
ENSG00000186827	TNFRSF4	100.305364629256	1.0858936485004	0.308606473230815	3.51870016572279	0.000433666529183388	0.0191765036550757
ENSG00000186891	TNFRSF18	83.4908621014247	1.48820020261424	0.317076280741813	4.69350844144676	2.68558903370586e-06	0.000789373604918908
ENSG00000187260	WDR86	27.7692740641207	1.01033511636418	0.251836780821177	4.01186480016833	6.02409986423381e-05	0.00505054063785294
ENSG00000187474	FPR3	133.192136613944	1.33276014872808	0.313551811333808	4.25052607114179	2.13269008413909e-05	0.00274655304443974
ENSG00000187555	USP7	2894.12136209226	-0.471644371666285	0.110359585087867	-4.27370555344844	1.92250883181154e-05	0.00256855404566736
ENSG00000187688	TRPV2	168.362928577786	1.2232680628164	0.295073243936255	4.14564210057846	3.38862770191904e-05	0.00368221921613172
ENSG00000187783	TMEM72	10.4672153922431	-2.93087860926213	0.654762776434517	-4.47624500772952	7.59673019214974e-06	0.00153383930916891
ENSG00000187957	DNER	18.2135462663654	3.27351864070488	0.644843613443492	5.0764535345619	3.84544781989405e-07	0.000223429461237751
ENSG00000188015	S100A3	101.49957542538	2.7254062854921	0.477987334004913	5.70183787644901	1.18522527676087e-08	2.11511916532811e-05
ENSG00000188064	WNT7B	93.1387765389531	2.38062733954162	0.418911057831538	5.6828944832938	1.32433983364279e-08	2.18023935879327e-05
ENSG00000188211	NCR3LG1	336.518149185816	-0.927544739798462	0.261006008566106	-3.55372945203114	0.000379809716727489	0.0174754437619145
ENSG00000188263	IL17REL	10.0625823146241	2.52008662598817	0.685025748721295	3.67882029353246	0.000234315293390404	0.012698770694286
ENSG00000188290	HES4	714.39338908411	0.898308545495873	0.287539968665141	3.12411714331795	0.00178339440712576	0.0491693731874959
ENSG00000188389	PDCD1	48.5117380309161	1.75990872083886	0.48802151688228	3.60621132461949	0.000310700142455246	0.0152505547330096
ENSG00000188536	HBA2	747.724418197453	3.05878974171264	0.467205880280719	6.54698468237337	5.87103826059134e-11	4.88940066342047e-07
ENSG00000188641	DPYD	135.955865223542	1.68491013408501	0.36457186897561	4.62161312341334	3.80767607495296e-06	0.0010046258862384
ENSG00000188820	CALHM6	63.1971495985424	2.04705782801391	0.38607521565124	5.30222543439077	1.14399447734632e-07	0.000102076992935788
ENSG00000188868	ZNF563	57.5305866351545	-0.811120622819233	0.233732970140545	-3.47028757787788	0.000519901396341453	0.0214343506372852
ENSG00000188883	KLRG2	10.7277900804805	-3.67130133809044	0.833878020278785	-4.40268390437134	1.06919890008851e-05	0.00183751667366846
ENSG00000189350	TOGARAM2	52.8204884599350	1.6723673471206	0.350207835493269	4.77535673856373	1.79389861017554e-06	0.000574596641290072
ENSG00000196126	HLA-DRB1	4179.24430520027	1.38618266024632	0.377960858055666	3.6675296679588	0.000244905141801293	0.0130741668007767
ENSG00000196154	S100A4	1614.59538902661	1.10927003020102	0.325655240479671	3.40627108769116	0.000658567517668017	0.0247423321224327
ENSG00000196187	TMEM63A	2950.12446317821	-0.588659774000658	0.186837381104648	-3.15065310014675	0.00162905851654798	0.0463030693713705
ENSG00000196209	SIRPB2	20.5131432743228	1.31178091625621	0.383379981827909	3.42162078990613	0.000622490675681082	0.0240254594200237
ENSG00000196227	FAM217B	359.533925770545	-0.798952477741992	0.235770160868217	-3.38869208384925	0.000702268226963381	0.0259932879740046

ENSG00000196329	GIMAP5	135.811544221358	1.24791665997777	0.313532755586777	3.98017954341737	6.88632380618559e-05	0.00549673846561472
ENSG00000196664	TLR7	27.1510065041052	1.2075948159506	0.298401618139156	4.04687757218345	5.19053709817056e-05	0.00463300480097051
ENSG00000196735	HLA-DQA1	728.232238112254	1.81373609209229	0.36144412379033	5.01802622511142	5.22050519990585e-07	0.000266181840641 73
ENSG00000196743	GM2A	1087.45574380328	0.605560455935045	0.179175183459501	3.37971165561508	0.000725619125675682	0.0265080437597578
ENSG00000196954	CASP4	681.449285588644	0.710879836132827	0.195452749761243	3.63709304167482	0.000275732351473148	0.0139451357676217
ENSG00000197044	ZNF441	98.9725346533437	-0.650859592805497	0.191792836499819	-3.39355527914157	0.000689916325065758	0.0256637191645867
ENSG00000197057	DTHD1	4.33870403242815	1.73408600169791	0.510822409565056	3.39469445589597	0.000687052298447065	0.0256581683473863
ENSG00000197122	SRC	2334.39303771701	-0.484154155926087	0.145114760362932	-3.33635361913025	0.00084885117298657	0.0299543752908142
ENSG00000197183	NOL4L	1222.30693938419	-0.623376219661287	0.190710671841459	-3.26870129312695	0.00108042295936467	0.0352139972002989
ENSG00000197191	CYSRT1	48.3872196577523	1.35531593029356	0.434736474211615	3.11755744155443	0.00182356406902837	0.0497024505499483
ENSG00000197273	GUCA2A	248.398450876288	-1.94474310744389	0.613721942501226	-3.16876906749999	0.00153085977236758	0.0448382187020299
ENSG00000197296	FITM2	522.005779309078	-0.901527863905569	0.22271427894849	-4.0479122764916	5.16765058814822e-05	0.00463300480097051
ENSG00000197415	VEPH1	24.2059013111383	1.72474709006447	0.519639913336451	3.31911973233616	0.000903017015852562	0.0310757260661989
ENSG00000197467	COL13A1	119.534290748306	1.37768865128836	0.332410679088496	4.14453788026944	3.40499669538994e-05	0.00368221921613172
ENSG00000197471	SPN	128.556681265941	1.07955063400586	0.313401215363874	3.44462810315669	0.000571845589604	0.0229694376377272
ENSG00000197506	SLC28A3	228.632883324342	1.75059682755235	0.459293490100184	3.81149932512759	0.000138126432854759	0.00889420308877137
ENSG00000197535	MYO5A	168.681754128125	0.872832367446549	0.250693241079649	3.48167490949321	0.000498288234452891	0.0210646924696633
ENSG00000197540	GZMM	26.0721879307574	1.60127248054593	0.418327187428541	3.82779921713662	0.000129294158374648	0.0085007506653479
ENSG00000197629	MPEG1	228.457255641818	1.26818845336133	0.315363630478719	4.02135291072161	5.7864836038092e-05	0.00491733014821663
ENSG00000197646	PDCD1LG2	25.5429266542971	1.79202380307446	0.405320726783583	4.42124886455978	9.81320721128626e-06	0.00177674143500766
ENSG00000197860	SGTB	144.263137120916	0.821860769710554	0.202222260063596	4.06414590288968	4.82086642663522e-05	0.00447749170271577
ENSG00000197872	FAM49A	60.8568714636291	0.957728031141982	0.29635542450671	3.23168719700724	0.00123061688205666	0.038874329643661
ENSG00000198010	DLGAP2	4.26016159993885	4.62326167008743	0.929695005530278	4.97287996879194	6.59655075198647e-07	0.000310958913184207
ENSG00000198019	FCGR1B	12.2489637573948	1.41086675138106	0.419396038067418	3.36404406174735	0.000768092794660877	0.0276115545061976
ENSG00000198216	CACNA1E	20.5081604933782	2.38532826186193	0.556414469976441	4.28696302948938	1.8113242457922e-05	0.00248649038224573
ENSG00000198821	CD247	79.3091495268358	1.43252817475423	0.312510696749049	4.58393325302581	4.56309952348417e-06	0.00109414212713492
ENSG00000198829	SUCNR1	22.4779338832099	1.49614988002609	0.361899346763161	4.13416021169338	3.56254899960954e-05	0.00378752017898914
ENSG00000198844	ARHGEF15	98.5981887975632	1.00142872585482	0.31919349687431	3.13737195670109	0.00170469714248813	0.0478541049527229
ENSG00000198851	CD3E	162.481113940948	1.34820207830279	0.377044053690205	3.57571499963378	0.000349271993944572	0.0164793224025849
ENSG00000198879	SFMBT2	65.2498473566718	1.1248801059603	0.30833967530699	3.64818476519555	0.000264099688354267	0.0136046734306041
ENSG00000198959	TGM2	2748.46736092639	1.10754687125407	0.329461401587608	3.36168930841982	0.000774672343089188	0.0277681690383648
ENSG00000203747	FCGR3A	498.120698590573	1.63834635766305	0.402785236051587	4.06754322408485	4.75113976172831e-05	0.00444578561074982
ENSG00000203814	HIST2H2BF	57.7431330310823	1.43971717206485	0.389712282822102	3.69430791772621	0.000220486490644938	0.0121603410204705
ENSG00000204136	GGTA1P	31.3752903377925	1.35364441709657	0.355848408115683	3.80399177353215	0.00014238291034093	0.00912126828707127
ENSG00000204160	ZDHHC18	537.720253406809	0.43128182558631	0.123449165766914	3.49359854242043	0.000476557067794425	0.0203928445450998
ENSG00000204161	TMEM273	70.6755580691706	1.41353842540046	0.34438995318818	4.10447056400648	4.05241863703043e-05	0.0041202874359633
ENSG00000204252	HLA-DOA	272.465683699873	2.8052925778964	0.457748462861522	6.12845876173933	8.87344206375369e-10	3.16705823601175e-06
ENSG00000204257	HLA-DMA	779.34510978711	1.40667719668118	0.368145685948397	3.82097971094615	0.00013292258822449	0.00867777280311625
ENSG00000204287	HLA-DRA	5839.89430695246	1.63760240031915	0.390002101237169	4.19895780954555	2.68146175077073e-05	0.00319017335148838
ENSG00000204397	CARD16	243.92871276576	0.967232021809549	0.24727576066179	3.91155210369558	9.1704866222675e-05	0.0065915977852098
ENSG00000204472	AIF1	192.349336091701	1.55954800794937	0.310339184108072	5.02530163063867	5.02641888937403e-07	0.000261625103191918
ENSG00000204475	NCR3	4.33048249830007	1.4841784996063	0.437370824928387	3.39340992817551	0.000690282551977357	0.0256637191645867
ENSG00000204482	LST1	99.2110467521796	1.46172359210679	0.347581300111276	4.20541493929284	2.60603589871081e-05	0.00314773102583305
ENSG00000204577	LILRB3	92.0001014111368	1.36734044939144	0.39300854246622	3.47916215971049	0.000502984125807258	0.021167235897171
ENSG00000204592	HLA-E	13500.298343276	0.63176727495097	0.197354780702463	3.20117542986424	0.00136868174758323	0.0421641735901594
ENSG00000204682	CASC10	15.0537818846888	1.18472837458614	0.354135759803999	3.34540735237198	0.000821618033172 72	0.0291995802856149
ENSG00000204832	ST8SIA6-AS1	21.2187674874167	3.54197259534482	0.878300905507215	4.03275525863127	5.51266837375973e-05	0.00476772190386881
ENSG00000205076	LGALS7	5.82312596875079	4.22326606383159	1.11860744062699	3.77546752367785	0.000159707832477825	0.00985219873240984
ENSG00000206172	HBA1	299.357451057837	2.95104248920861	0.469867690514792	6.28058185055334	3.37308238996754e-10	1.68546180861898e-06
ENSG00000206418	RAB12	379.655427164568	0.447542585548445	0.143414722080897	3.12061815847606	0.00180471879518498	0.0494940662776087
ENSG00000211644	IGLV1-51	320.332379802013	2.55023898924534	0.667268350310817	3.82190911356342	0.00013242249839028	0.00867778280311625
ENSG00000211651	IGLV1-44	353.13116300972	2.33520008619152	0.653125421259119	3.5754236631758	0.000349661194492614	0.0164793224025849
ENSG00000211662	IGLV3-21	786.391706730033	3.85881195617179	0.695384163736678	5.54918008980287	2.8701241513784e-08	3.5853590899019e-05
ENSG00000211669	IGLV3-10	129.712946446768	3.17409535265395	0.827496323275687	3.83578181965707	0.000125165487823718	0.0082893354857424
ENSG00000211673	IGLV3-1	541.268028008691	3.54693691077272	0.691772915594041	5.12731393614461	2.93905205943248e-07	0.000198457504467192
ENSG00000211677	IGLC2	5018.66771756167	2.28795183117601	0.558003019883819	4.10024990841874	4.12704221950988e-05	0.00415766221017076
ENSG00000211694	TRGV10	1.88760497402989	2.0138453449157	0.548849306102763	3.66921361029223	0.000243297725949974	0.0130658812996552
ENSG00000211745	TRBV4-2	1.71933789791379	1.91990081217755	0.558912683142626	3.43506395557608	0.000592413998296735	0.0233821032123943
ENSG00000211746	TRBV19	3.29069296384783	1.90256448883875	0.508269154731715	3.74322240711813	0.000181675288757963	0.0106548718646219
ENSG00000211751	TRBC1	110.409118070192	1.31709608776057	0.3985499710929	3.30472006847434	0.000950713047500671	0.03233490468913
ENSG00000211829	TRDC	15.7060837638256	1.85973453380217	0.533841209051419	3.48368485285489	0.000494561461 2518	0.0209425822846017
ENSG00000211893	IGHG2	5351.98469596965	2.5698953690514	0.60188492243441	4.26974538364757	1.95696241650122e-05	0.00257619780418335
ENSG00000211896	IGHG1	22940.1721929944	2.53538200646478	0.597151327546658	4.24579480863112	2.17819901242891e-05	0.00279077559623199
ENSG00000211897	IGHG3	4333.44868206423	2.67162872702321	0.572369294353624	4.66766605642832	3.04640512344176e-06	0.000855184107910886
ENSG00000211934	IGHV1-2	241.835976161676	2.11081138411234	0.674949448720226	3.12736218707143	0.00176382490421471	0.0490351685946927
ENSG00000211943	IGHV3-15	232.968684979014	2.6450857574272	0.674646754966522	3.92069736933435	8.82930876287371e-05	0.00646895748186618
ENSG00000211945	IGHV1-18	195.045231642715	2.23389266994312	0.670606568142103	3.33115238661032	0.000864872430022004	0.0302676034639377
ENSG00000211946	IGHV3-20	49.6297631505664	3.37958575209316	0.72745778657005	4.64574826812679	3.38845992259628e-06	0.000920187855501581
ENSG00000211947	IGHV3-21	297.09950547219	2.99574134265177	0.660475780691106	4.535732316357	5.74039505636703e-06	0.00125805289551118
ENSG00000211949	IGHV3-23	761.974410947307	2.02107525649501	0.648830166929561	3.11495266328211	0.00183974431588649	0.0498547522664581
ENSG00000211950	IGHV1-24	41.2301853830438	2.33038843536458	0.656848332569181	3.54783337311607	0.000388413860831489	0.0177731353461794
ENSG00000211962	IGHV1-46	117.360882001033	2.82308369983492	0.693284063872786	4.07204470280878	4.66022323943773e-05	0.00437974021920927
ENSG00000211965	IGHV3-49	90.5399522054009	3.43997163150063	0.713680519331435	4.82004417708241	1.43526435139447e-06	0.000499900661140903
ENSG00000211970	IGHV4-61	167.461594376983	2.1673324838721	0.645210765409669	3.35910775217146	0.000781945715447867	0.0279486863444199
ENSG00000211976	IGHV3-73	31.8103905178276	2.1627882096061	0.602888035187196	3.58737955204992	0.000334017943538568	0.0160174746667324
ENSG00000211979	IGHV7-81	1.62915313447894	-2.46477616331774	0.740179567025009	-3.32997055461065	0.000868551685572155	0.0302676034639377
ENSG00000213203	GIMAP1	74.6511780396175	0.965852779033064	0.272471066966958	3.54479024060999	0.00039292564235562	0.0179467170906998
ENSG00000213402	PTPRCAP	222.152947177842	1.64947549381286	0.40352846730738	4.0876310531926	4.35800498010237e-05	0.00422498284918415
ENSG00000213654	GPSM3	277.874411203212	1.01229635701864	0.259809910394644	3.89629616314861	9.76749393294635e-05	0.00687076781919746
ENSG00000213742	ZNF337-AS1	70.5924992324598	-0.897458916645644	0.288233717807464	-3.11365000414398	0.00184788550139255	0.0499649040766142
ENSG00000213809	KLRK1	54.9611439545626	1.44432790915854	0.440012370619106	3.28247114308704	0.00102901517174323	0.0339615786668863
ENSG00000213886	UBD	2377.07337390338	1.63509703385602	0.511745217391318	3.19513886654598	0.00139763574366675	0.0424574558316833
ENSG00000214039	LINC02418	194.359651114632	-1.82372162271062	0.540671110609062	-3.3730702212963	0.000743349923318364	0.0269157311365014
ENSG00000214078	CPNE1	3105.1089537191	-0.886592028963362	0.223015504499557	-3.97547260650267	7.02396290239538e-05	0.0055533762390331
ENSG00000214212	C19orf38	21.0738725396298	1.10508105759704	0.338456187957679	3.26506383075857	0.00109439411243614	0.035463479254351
ENSG00000214357	NEURL1B	838.637129771947	-0.753221121664416	0.217885405598517	-3.45695995376729	0.000546306026984799	0.0221933492328264
ENSG00000215458	AATBC	36.741583202896	1.45848404340515	0.411618969826037	3.54328675381882	0.0003951727581355	0.017956308943826
ENSG00000215808	LINC01139	26.0526852974745	2.31410852026148	0.684977697978263	3.37837060548344	0.000729167388765902	0.0265381642260818
ENSG00000215861	AC245297.1	9.36578390691746	1.75540696442506	0.463054593051001	3.79092873878852	0.000150085014925062	0.0094214171178084
ENSG00000216490	IFI30	2059.95772151546	1.31127254079897	0.259761034517035	5.04799557880198	4.4646959357382e-07	0.000246765069461487
ENSG00000216588	IGSF23	13.3479933895561	-2.60297314578927	0.682730611974766	-3.81259181899036	0.00013751711675096	0.00887784921164335
ENSG00000220842	RPL21P16	14.1340722826736	-22.7256311608651	3.40810117295778	-6.66812104675352	2.59099052641767e-11	3.23666536560095e-07
ENSG00000222001	AC106876.1	102.350301423495	-1.08066006867675	0.276649807584624	-3.90623828012673	9.37440743536166e-05	0.00669171986757359
ENSG00000222041	CYTOR	362.450038296764	0.719981211302698	0.218659600090161	3.29270341208813	0.000992290895511113	0.0330136987358669
ENSG00000223813	AC007255.1	48.3324066616293	-0.949841657591071	0.264415694762794	-3.59222873832497	0.000327861867396141	0.0158133221911683
ENSG00000223865	HLA-DPB1	1638.30996070374	1.75684262336122	0.361747882668655	4.85653878718183	1.19455390862007e-06	0.000464420001763836
ENSG00000223891	OSER1-DT	125.717823395261	-0.996055580871391	0.279471426129181	-3.56406948168999	0.00036514922959662	0.0169946638690228
ENSG00000224041	IGKV3D-15	24.8806115799813	2.82819339880486	0.840059850583113	3.36665702668889	0.000760852511685991	0.0274302152264975

ENSG00000224557	HLA-DPB2	3.40403502334082	1.82372854903586	0.583868366579506	3.12352689994127	0.0017869752983608	0.0491693731874959
ENSG00000224650	IGHV3-74	133.052907795603	2.78787166230686	0.66803104462119	4.17326662399011	3.00263210386195e-05	0.00344118167352692
ENSG00000225217	HSPA7	66.2049719154138	1.92755365512114	0.476946331586355	4.04144770064584	5.3122229497522e-05	0.00467325979495102
ENSG00000225492	GBP1P1	16.0976509627751	1.65108978330267	0.449736487708641	3.67123822155236	0.00024137822615029	0.0130532329050624
ENSG00000225783	MIAT	226.630141156525	1.76665461306831	0.444872867481875	3.97114488700592	7.15280597618179e-05	0.00559177930742689
ENSG00000226025	LGALS17A	22.4132172625709	3.05105467540938	0.802737562543432	3.80081214306488	0.000144222613540796	0.00921549303504665
ENSG00000226147	TUBBP10	2.20830260082238	-3.71318755413541	1.18288742954666	-3.13908784672645	0.00169474633736222	0.0477572664603769
ENSG00000226789	AC110926.1	38.7984202280457	-1.14411169466323	0.332289566907584	-3.44311651223595	0.000575051510597336	0.0230241777896857
ENSG00000226812	AL117382.1	134.123576493577	-1.49151549740517	0.411872217993325	-3.6213063961244	0.000293119117987181	0.0145015604827559
ENSG00000226979	LTA	6.81852399713833	1.64818172368443	0.411899444212427	4.00141769269886	6.29640974101284e-05	0.00515768855637589
ENSG00000227039	ITGB2-AS1	38.7942605431083	1.82506480170048	0.409426661474908	4.45761102886147	8.28781128530678e-06	0.00158063112329851
ENSG00000227507	LTB	178.121593317161	1.31854426991267	0.359374533318256	3.66899751559464	0.000243503445299903	0.0130658812996552
ENSG00000228495	LINC01013	2.8449389385656	1.90332739606767	0.519314255003313	3.66507827915397	0.000247262942674951	0.0130932240415779
ENSG00000229671	LINC01150	1.15970627380413	2.08768754434906	0.657570324583245	3.1748506073053	0.00149913661318324	0.0441159353872439
ENSG00000230006	ANKRD36BP2	41.2201798762455	1.4646446673961	0.415560340269417	3.52450540984382	0.000424274151205714	0.0189286882030778
ENSG00000230873	STMND1	9.44164243298128	-2.14904656048289	0.632529763227252	-3.39754219551372	0.000679941011678289	0.0254306081373808
ENSG00000231233	CFAP58-DT	2.99347863317207	2.04880525481622	0.499878394055444	4.09860733966626	4.15643472410968e-05	0.0041704564316127
ENSG00000231389	HLA-DPA1	2339.25175611412	1.74907124193429	0.401068248152146	4.36103144537828	1.29450758978712e-05	0.00203408664297116
ENSG00000231473	RB1-DT	4.02131339803876	-3.12429559593586	0.786189868728626	-3.97397081825573	7.06842271618361e-05	0.00557089820634483
ENSG00000231475	IGHV4-31	84.0305473892919	3.30874954591563	0.666653867306952	4.9632196079231	6.93341096128934e-07	0.000314953344467005
ENSG00000231764	DLX6-AS1	34.0183397167356	-3.060225723324	0.918102167011085	-3.33320825642605	0.000858506553803789	0.0302038463157562
ENSG00000231999	LRRC8C-DT	3.41512920472441	1.30361962704514	0.398793694310026	3.26890732136727	0.00107963658230879	0.0352139972002989
ENSG00000232070	TMEM253	81.2132884064466	-1.32057936526402	0.422456246260245	-3.12595535503221	0.00177228455175342	0.0490351685946927
ENSG00000232629	HLA-DQB2	136.003107946498	1.69213108839035	0.502219321441463	3.36930702612878	0.000753574374733338	0.0272222827047922
ENSG00000232810	TNF	18.1425375637783	1.53072696438189	0.376573430728972	4.0648830731863	4.80565481429627e-05	0.00447749170271577
ENSG00000233532	LINC00460	65.3212796095473	2.20069030369955	0.645076749578957	3.41151700962087	0.000646024705242965	0.0244179746381093
ENSG00000234336	JAZF1-AS1	1.50727269097948	3.15352658374454	0.729664291931158	4.32188695351707	1.54700450285812e-05	0.00220859202853756
ENSG00000234663	LINC01934	32.7827620013525	1.7630596353324	0.405325033270434	4.34974277583315	1.36297313361311e-05	0.00206378913758727
ENSG00000234883	MIR155HG	41.0189593276851	1.34612675430895	0.342679499051147	3.92823836277416	8.55703769344843e-05	0.00630646105407421
ENSG00000235027	AC068580.3	31.5083209781536	0.924818474921192	0.259567234657003	3.56292455842226	0.000366746101537944	0.0169996003725862
ENSG00000235049	LINC00940	2.14855726999094	-3.66435810632959	0.896147690650842	-4.08901138122478	4.33215599477874e-05	0.00422498284918415
ENSG00000235296	AC145207.1	1.26516829790422	-3.26592874264485	0.851038788609705	-3.83757918717221	0.000124253172652998	0.00825622677011305
ENSG00000235505	CASP17P	40.0274294050498	1.24719949986307	0.342521877486688	3.6412258072816	0.000271343011787895	0.0138351710336914
ENSG00000235568	NFAM1	102.318669835649	1.1898825595917	0.284332861993859	4.18482250432735	2.85389008139041e-05	0.00331635301364921
ENSG00000235641	LINC00484	2.51685336893892	1.90817154989127	0.597152073791616	3.19545327501828	0.00139611386855153	0.0424574558316833
ENSG00000235831	BHLHE40-AS1	23.0870487438646	1.11328789348594	0.268702129688175	4.14320457667342	3.42486172143394e-05	0.00368221921613172
ENSG00000236283	AC019197.1	7.6131657673256	2.53339826070384	0.797464321603951	3.17681705886024	0.00148900930252596	0.0440253354015487
ENSG00000236391	AC092573.2	2.97877730891151	-2.24412752893954	0.591231849125447	-3.79568105517161	0.000147238692841641	0.00933657741613086
ENSG00000236432	AC097662.1	11.4755582657599	-1.37057054298934	0.419739681256029	-3.26528704383642	0.00109353198297222	0.035463479254351
ENSG00000236481	LINC02195	1.67518420464011	2.09953805800601	0.610260103570125	3.4403986852874	0.000580857861054992	0.0231085235678311
ENSG00000236935	AP003774.4	66.0094551088634	-2.15763856368224	0.605494831277519	-3.56343019333442	0.000366040068214778	0.016998410900145
ENSG00000237181	AC147651.3	12.0945099489135	1.20873828561312	0.366027320795962	3.30231711388264	0.000958895957478097	0.0324726826188625
ENSG00000237517	DGCR5	13.5505987943749	1.40341636422361	0.384881468616657	3.64635992807807	0.000265981356284456	0.0136734119452898
ENSG00000237541	HLA-DQA2	339.147657334761	2.59043165037803	0.6160001169282	4.20524538744522	2.60799040324785e-05	0.00314773102583305
ENSG00000237721	AF064858.3	5.4481014129346	-3.38271940236661	0.860960183185353	-3.92900794767457	8.52970207598683e-05	0.00630491351084187
ENSG00000237976	AL391069.2	3.76522940447741	1.43417669534568	0.385682465075475	3.71854264897684	0.00020037546512563	0.0114036005027306
ENSG00000238266	LINC00707	6.063890221855	0.864499631052715	0.25014850468003	3.45594562781223	0.000548365923520919	0.022240867261764
ENSG00000238271	IFNWP19	3.42294982719805	2.53232123365449	0.806909806788133	3.1382952745788	0.00169933597035203	0.0477572664603769
ENSG00000239571	IGKV2D-30	1.22781468090279	3.6271228931463	1.13501433777774	3.19566262065718	0.0013951013913266	0.0424574558316833
ENSG00000239713	APOBEC3G	159.136739920212	1.53853882938478	0.268453185835075	5.731125241069	9.97665664815604e-09	1.91735992075023e-05
ENSG00000239975	IGKV1D-33	21.4031326059289	2.49308121916816	0.72560604365051	3.43586060367623	0.000590674804890033	0.0233821032123943
ENSG00000240350	AC017002.3	7.91985238771532	1.47318091678062	0.397260448764599	3.70835033127994	0.000208613906210127	0.0116339505195398
ENSG00000240476	LINC00973	2.17287092191864	4.17721992649814	0.966268341172489	4.32304334987259	1.53891480661299e-05	0.00220859202853756
ENSG00000240864	IGKV1-16	167.753614192218	3.41029550900081	0.786394353709912	4.33662257735234	1.44688753616564e-05	0.00210168826764897
ENSG00000241127	YAE1	475.143563174702	-0.775890843035289	0.234376703303519	-3.31044353853935	0.000931482422191364	0.0318360558632408
ENSG00000241186	TDGF1	431.058810399312	-1.58559475087418	0.495869663223157	-3.19760386341807	0.00138574498106264	0.0424574558316833
ENSG00000241794	SPRR2A	18.1144954772952	4.5108685956982	0.993553322109719	4.54013739908774	5.62175814064614e-06	0.00124295579987525
ENSG00000241978	AKAP2	852.343425987231	0.858560213162529	0.27192786798891	3.15730866244847	0.00159232698037598	0.0458043893927425
ENSG00000242076	IGKV1-33	398.529277290252	2.28911426560028	0.676719428126919	3.38266373101816	0.00071786472078231	0.0263364642937228
ENSG00000242372	EIF6	4847.04327678908	-0.620730163388298	0.17615492060264	-3.52377419412828	0.0004254466469471796	0.0189401392207324
ENSG00000242574	HLA-DMB	666.160166888422	2.28530422380307	0.402778510902394	5.67384843268582	1.39624678757174e-08	2.18023935879327e-05
ENSG00000242600	MBL1P	2.55908547513854	2.13162367687961	0.673368840416669	3.16561080486082	0.0015475770656995	0.045248920567613
ENSG00000243064	ABCC13	22.4001774351102	-2.23871215978205	0.648437237946111	-3.45247315973563	0.000555472735465652	0.0224925945265379
ENSG00000243264	IGKV2D-29	17.5580989073617	2.36299633305739	0.724578039089766	3.26120335640598	0.00110940445635386	0.035856870553098
ENSG00000243290	IGKV1-12	229.545480907594	3.41164132597406	0.699456774208753	4.87755848791858	1.07407005528927e-06	0.000425945496211859
ENSG00000243709	LEFTY1	1434.88969979322	-2.61418682018582	0.658344056203572	-3.97085201203285	7.16160561210231e-05	0.00559177930742689
ENSG00000243811	APOBEC3D	72.5553037986575	0.960915343707143	0.255528454054898	3.760502317682	0.000169572503611309	0.0101354053354664
ENSG00000244005	NFS1	662.572541133867	-0.786510019221352	0.150316981835446	-5.23234307672724	1.67374726697938e-07	0.000126717885508766
ENSG00000244437	IGKV3-15	593.999810690162	2.23925314923261	0.622020095536595	3.59996914135207	0.000318254947345271	0.0155298468837388
ENSG00000244682	FCGR2C	36.2085490846299	1.65621276918991	0.427267141903474	3.87629332274766	0.000106059817795403	0.00736055135500097
ENSG00000244734	HBB	629.548153244019	3.51217558523866	0.479962279532019	7.3176075183724	2.52430868590545e-13	6.30673282086617e-09
ENSG00000246627	CACNA1C-AS1	6.28116289546462	1.76402309653161	0.555411993646801	3.17606230427279	0.00149288884405664	0.044035814498124
ENSG00000247134	AC090204.1	7.67243210727754	2.31568833084467	0.541843946105627	4.27371819411867	1.92239978830625e-05	0.00256855404566736
ENSG00000247774	PCED1B-AS1	45.1357957149357	1.31784883750109	0.368863834684804	3.57272444078774	0.000353286463749143	0.0165911823502041
ENSG00000248323	LUCAT1	55.0630091138154	1.68627119292994	0.379489068781883	4.44353034553191	8.84946681766442e-06	0.00164996327591439
ENSG00000248409	AP000344.2	10.1579418781041	-2.15380238813333	0.63848824684287	-3.37328431460286	0.000742772131609957	0.0269157311365014
ENSG00000249138	SLED1	2.14352329774648	1.94104882068824	0.526401649563556	3.68739121979877	0.000226564895457048	0.0124406535123053
ENSG00000250378	AC114296.1	5.67301829807604	-2.34823276653171	0.73254787057505	-3.20556902948656	0.0013479571060335	0.0418872641009218
ENSG00000251003	ZFPM2-AS1	6.92663324252202	1.93285201263245	0.432875672446462	4.46514354043309	8.00150883059166e-06	0.00156179450487111
ENSG00000251442	LINC01094	18.23635645317	1.35336570990176	0.36182341012081	3.74040394304471	0.000183724750907667	0.0107247177025167
ENSG00000253755	IGHGP	83.5838926402497	3.27554044621413	0.655166184748773	4.99955663534459	5.74622923804922e-07	0.000281497629967494
ENSG00000253882	AC099548.2	62.085796299107	0.907180917685101	0.283241012768747	3.2028586143553	0.00136070766948335	0.0420823418223686
ENSG00000254777	AC022182.1	2.39510882613473	1.72744891748184	0.528852720200146	3.26640830518575	0.00108921076123205	0.0354333875763302
ENSG00000254838	GVINP1	44.2491113636752	1.18380119108233	0.369655273706747	3.20244637446039	0.0013626566816481	0.0420823418223686
ENSG00000255112	CHMP1B	1417.78303055228	0.481613976036869	0.153140569587445	3.14491435766707	0.00166135457052501	0.047007115050959
ENSG00000255177	MUC5B-AS1	15.424908241761	-8.60319913389838	2.60084587426334	-3.30784658138773	0.000940162987392639	0.0320450642251265
ENSG00000255221	CARD17	1.36756147855303	2.49062349623305	0.716590215230448	3.47565937030287	0.000509599103381928	0.0212906755834349
ENSG00000255398	HCAR3	77.2104895754852	3.35922515572659	0.610029019013639	5.50666452090779	3.65696200584688e-08	4.35073994067041e-05
ENSG00000255833	TIFAB	12.6021446633454	1.77537507174572	0.548447677437143	3.23709105678398	0.00120754874313967	0.038237513052727
ENSG00000255986	MT1JP	6.9745507617921	2.70311367654212	0.777394852817723	3.47714377930918	0.000506785973481822	0.0212215499833531
ENSG00000256262	USP30-AS1	41.0381689003307	1.39478658449474	0.347457706100227	4.01426291605229	5.96318480953165e-05	0.00502725989345869
ENSG00000256633	AP005019.1	1.17919053716056	2.12450824933572	0.640856150441008	3.31510940149943	0.000916072650906383	0.0313952799866188
ENSG00000256643	LINC02441	18.187526555584	-3.03084845547679	0.594029204745158	-5.10218762186456	3.35749643288663e-07	0.000200067637467126
ENSG00000258017	AC011603.2	74.7095929839225	-2.88159257537845	0.658224576293067	-4.37782586546184	1.19869059904196e-05	0.00193381050183383
ENSG00000258227	CLEC5A	58.9131650973363	1.55949843715998	0.42791903326445	3.64437736097666	0.000268033990696585	0.0137348924739792
ENSG00000259347	AC087482.1	10.3064552379215	-1.67867553327995	0.434588385813595	-3.8626792341385	0.000112150225250102	0.00770269252078768
ENSG00000259768	AC004943.2	92.4792892148293	-0.738276119105127	0.213548894424622	-3.45717603031526	0.000545868150386995	0.0221933492328264
ENSG00000260032	NORAD	10562.4929186543	-0.71312357534168	0.177922511145572	-4.00805705107332	6.12203331123031e-05	0.00508149103813216

ENSG00000260196	AC124798.1	250.692301153143	-0.789071235993622	0.247649158853005	-3.18624638035612	0.00144131796293575	0.043031911545288
ENSG00000260314	MRC1	200.835131308374	1.59199868342292	0.412207087980136	3.86213320887786	0.00011240124670894	0.00770269252078768
ENSG00000261269	AC093278.2	22.0509919119768	0.988707682129736	0.285989719266081	3.45714414024043	0.00054593275467761	0.0221933492328264
ENSG00000261308	FIGNL2	9.78289606558232	1.57556591979085	0.494065038790696	3.18898484225336	0.00142773363789492	0.0428731937610176
ENSG00000261329	AC016597.1	1.92474172251259	2.24177996117752	0.617946218837283	3.62779137219355	0.000285856095846139	0.0142836573972399
ENSG00000261465	AC099518.4	3.05158722835704	-1.44232150288766	0.456573548395011	-3.15901240437129	0.00158304752369744	0.0456399319314087
ENSG00000261469	AC020978.4	4.20397158908117	-1.96665828441113	0.616463947224376	-3.19022433228414	0.00142162394914045	0.0427957962569227
ENSG00000262406	MMP12	1311.22574975211	1.69120570831199	0.438260903798253	3.8589016123841	0.000113897776451705	0.00773266860562338
ENSG00000262943	ALOX12P2	39.7901590239929	2.10044978498805	0.621800460975798	3.378012588945	0.000730117381114357	0.0265381642260818
ENSG00000266053	NDUFV2-AS1	13.8420729042766	0.834929225485935	0.233547469454763	3.57498724963773	0.000350244964608253	0.0164793224025849
ENSG00000266835	GAPLINC	16.8450999644616	1.23444555057619	0.385178548096524	3.20486578672821	0.00135125472826137	0.0419375753178659
ENSG00000267120	AD000671.2	10.6640761402597	1.35983990556557	0.414882621010295	3.27764971753741	0.0010467520290252	0.0344555558195861
ENSG00000267607	AC011511.5	13.9314951198194	1.48656475391621	0.405061325610174	3.6699745444147	0.00024257462367749	0.0130658812996552
ENSG00000267934	AC010300.1	14.1072055306417	-1.43239502567198	0.458165227612438	-3.12637218921303	0.00176977413885593	0.0490351685946927
ENSG00000269220	LINC00528	5.60513155487551	1.41473174637105	0.425034850350395	3.32850763932596	0.000873126103941684	0.0303544309385232
ENSG00000269416	LINC01224	91.6011436403842	-1.03452991061962	0.298059180779542	-3.47088758653204	0.000518741113494624	0.0214343506372852
ENSG00000270550	IGHV3-30	407.04721435242	2.47539401226385	0.632619239261939	3.91292875498353	9.11834431803255e-05	0.00658418249831576
ENSG00000270640	AC104695.3	4.29596655503811	1.71742510638652	0.489315598598287	3.50985153816131	0.00044835695449617	0.0194813046106649
ENSG00000271503	CCL5	509.803633167522	1.22796109186775	0.359110161593829	3.41945515108157	0.00062746675054599	0.0240713229328455
ENSG00000271605	MILR1	27.7694698154737	1.05766059183889	0.272713575513962	3.87828361622849	0.00010519603556454	0.00732094081488706
ENSG00000272449	AL139246.5	69.0736707194638	0.738156623582138	0.212173871157432	3.47901756024628	0.000503255608506227	0.021167235897171
ENSG00000272510	AL121992.3	6.39595574240399	-1.45631838569876	0.354937065951251	-4.10303269340369	4.07769601976811e-05	0.00412458126955006
ENSG00000272769	AC097532.2	43.4701195288218	-2.22172029166604	0.50698643636074	-4.38220854114764	1.17482274008683e-05	0.00193103758804798
ENSG00000272908	AC006033.2	2.6602002897837	2.13208329982448	0.507363033270946	4.20228349329876	2.64235928137643e-05	0.00317015020957119
ENSG00000273443	AL645608.8	2.08991520122684	2.21999656648009	0.640626240605013	3.4653537831724	0.000529534383611867	0.0217239524468947
ENSG00000273820	USP27X	73.9070646824949	-0.70514956277512	0.191558767411179	-3.68111348963488	0.000232217606964718	0.0126124449834924
ENSG00000274576	IGHV2-70	43.1327694554664	2.77548845187319	0.811974807438955	3.41819527705219	0.000630378614617037	0.0241184981739541
ENSG00000274825	AL023803.2	10.2149759690662	-0.908043227645409	0.259338747863577	-3.50137893055256	0.000462857219604402	0.0199379737493041
ENSG00000275302	CCL4	140.510583999306	1.7290250415073	0.383287685543425	4.51103728797312	6.45113781203132e-06	0.00135441367307387
ENSG00000275385	CCL18	390.466559627423	1.96988273481726	0.58217046680814	3.38368716231436	0.000715194430121275	0.0263023417510029
ENSG00000276070	CCL4L2	78.9240706206915	1.59883796228684	0.437591050258897	3.65372637612424	0.00025846170380091	0.0134249630099001
ENSG00000276231	PIK3R6	18.8086744846605	1.36750851458541	0.361854962786407	3.77916197156762	0.000157357047646909	0.0097553560258322
ENSG00000276980	AC008760.2	8.07080963332903	2.33455420525374	0.708387647281743	3.2955885301106	0.000982157521725293	0.0330114926882151
ENSG00000277150	F8A3	102.36818931951	-1.91733715440128	0.590868103297308	-3.24494949668409	0.001174714555937	0.0374350363080741
ENSG00000277632	CCL3	159.650001327731	2.11769463318158	0.415082737941704	5.10186148352669	3.36328881428887e-07	0.000200067637467126
ENSG00000277734	TRAC	168.694727705695	1.12683980344359	0.360240683403484	3.12801928088028	0.0017598863609169	0.0490178381729629
ENSG00000278535	DHRS11	575.416046282742	-1.21979116125835	0.264227857095887	-4.61643664171147	3.90385132938307e-06	0.00101597730847194
ENSG00000279249	AC007614.1	10.0688438319346	-2.3911804783275	0.657296426126725	-3.63790275327997	0.000274867163386386	0.0139440565315313
ENSG00000279369	AC046185.3	18.4759790533997	1.02173422096767	0.327748719393416	3.11743161913386	0.00182434263163771	0.0497024505499483
ENSG00000279519	AC007382.1	31.4671470287117	-0.696328973791539	0.204773023990186	-3.40049172602399	0.000672647715382334	0.0251955480076645
ENSG00000279611	AC012313.10	1.36541982770582	3.15654398794707	0.736540592614243	4.28563478998949	1.82218125193057e-05	0.00248696585590559
ENSG00000279901	AC092117.2	24.2620745493744	-1.44568414748247	0.366046908898806	-3.94945050029676	7.83308067401029e-05	0.00598476108744566
ENSG00000280721	LINC01943	17.0704495288395	1.43111396839192	0.298512523720328	4.79415051186497	1.63365591099018e-06	0.000537042885265508
ENSG00000281103	TRG-AS1	21.4154667436523	1.50674546204955	0.412834105985419	3.64976013416578	0.000262485302283937	0.0135494479178965
ENSG00000284648	AC097493.4	12.6275097793888	-1.29014884743825	0.407645276565862	-3.16488113957038	0.0015514631565821	0.0452824246542606
ENSG00000285219	AL591485.1	1373.99260355719	-4.67138702778051	0.796282451473775	-5.86649500957181	4.45103657346837e-09	1.01095179774122e-05

Supplementary Table 5. The correlation between levels of *P. micra* or *F. nucleatum* in tumour tissue and immune markers in blood.

		P. micra		*F. nucleatum*	
		r_s	*P*-value	r_s	*P*-value
T helper cells	**CD4**	0.024	0.882	-0.072	0.654
	CD28	0.051	0.746	-0.207	0.195
	CD69	-0.267	0.087	-0.232	0.144
	PD-1	-0.133	0.401	0.021	0.897
	CTLA-4	-0.106	0.502	0.116	0.470
	Treg	0.014	0.928	-0.152	0.319
Cytotoxic T cells	**CD8**	-0.056	0.716	0.247	0.111
	CD28	0.203	0.192	-0.002	0.990
	CD69	-0.181	0.246	-0.342	0.027*
	PD-1	-0.106	0.500	-0.153	0.334
	CTLA-4	0.055	0.725	0.254	0.105
	NKG2D	0.028	0.859	0.021	0.891
NK cells	**CD56/CD16**	0.306	0.036*	-0.014	0.927
	NKG2D	0.192	0.195	0.136	0.367
	CD69	-0.132	0.378	-0.177	0.240
B cells	**CD19**	-0.078	0.621	0.056	0.725
	CD86	-0.213	0.170	-0.271	0.082
	CD80	-0.002	0.989	-0.133	0.400
	HLA-DR	0.074	0.639	-0.133	0.401
	CD69	-0.154	0.324	-0.017	0.914
Macrophages	**CD14**	-0.112	0.460	-0.217	0.152
	HLA-DR	0.085	0.573	-0.158	0.301
	CD163	0.055	0.717	-0.005	0.972
	PD-L1	0.080	0.598	-0.017	0.909

Correlations were calculated using the relative levels of *P. micra* and *F. nucleatum*. Immune markers in bold are presented as the percentage of positive cells within isolated peripheral blood mononuclear cells. Remaining immune markers (not in bold) are defined as the percentage of cells (in bold) expressing a specific marker. Abbreviations: r_s, Spearman´s rank correlation coefficient. *P value < 0.05.

Supplementary Table 6. The correlation between levels of *P. micra* or *F. nucleatum* in tumour tissue and systemic plasma markers.

	P. micra (n=61)		*F. nucleatum* (n=60)	
	r_s	*P*-value	r_s	*P*-value
IL8	0.327	0.010*	0.128	0.330
TNFRSF9	0.040	0.759	0.010	0.940
TIE2	-0.086	0.512	-0.110	0.403
MC3	0.159	0.220	0.159	0.226
CD40-L	-0.096	0.462	0.173	0.187
IL-1alpha	-	-	-	-
CD244	-0.147	0.259	-0.116	0.377
EGF	-0.006	0.966	0.120	0.363
ANGPT1	0.116	0.375	0.139	0.289
IL7	0.095	0.467	-0.014	0.914
PGF	-0.028	0.834	-0.098	0.461
IL6	0.199	0.124	0.120	0.361
ADGRG1	0.112	0.391	0.043	0.741
MC1	0.101	0.439	0.143	0.277
CRTAM	-0.307	0.016*	0.108	0.412
CXCL11	0.094	0.471	0.106	0.420
MC4	-0.092	0.482	-0.062	0.638
TRAIL	-0.041	0.755	-0.028	0.831
FGF2	-0.118	0.364	0.031	0.813
CXCL9	0.053	0.683	-0.037	0.782
CD8A	-0.224	0.083	-0.030	0.820
CAIX	0.311	0.015*	0.173	0.187
MUC-16	-0.133	0.307	-0.016	0.901
ADA	-0.124	0.342	-0.235	0.071
CD4	0.024	0.853	-0.027	0.840
NOS3	0.084	0.521	-0.074	0.576
IL2	-0.123	0.346	0.038	0.775
Gal-9	0.025	0.845	-0.030	0.822
VEGFR-2	-0.109	0.404	-0.032	0.809
CD40	-0.030	0.818	-0.026	0.842
IL18	-0.055	0.673	-0.157	0.230
GZMH	-0.041	0.753	0.163	0.212
KIR3DL1	-0.070	0.591	-0.117	0.373
LAPTGF-β1	0.057	0.664	0.084	0.525
CXCL1	0.027	0.836	0.003	0.981
TNFSF14	0.293	0.022*	0.167	0.203
IL33	-0.123	0.346	0.162	0.217
TWEAK	-0.096	0.462	-0.073	0.581
PDGF-B	0.081	0.537	0.109	0.408
PDCD1 (PD-1)	-0.105	0.422	-0.046	0.726
FASLG	-0.288	0.025*	0.048	0.713
CD28	-0.134	0.304	-0.115	0.381
CCL19	0.042	0.748	-0.173	0.185
MC2	-0.105	0.421	-0.188	0.151
CCL4	-0.059	0.650	-0.221	0.090
IL15	-0.040	0.757	0.070	0.595

Gal-1	-0.045	0.731	-0.118	0.369
PD-L1	-0.103	0.430	-0.084	0.521
CD27	-0.074	0.571	-0.044	0.738
CXCL5	-0.065	0.618	0.072	0.584
IL5	0.125	0.337	-0.056	0.669
HGF	0.171	0.188	0.047	0.720
GZMA	-0.156	0.231	0.039	0.770
HO-1	-0.183	0.159	-0.238	0.067
CX3CL1	-0.182	0.159	-0.137	0.297
CXCL10	0.047	0.721	-0.036	0.786
CD70	-0.110	0.399	-0.110	0.404
IL10	-0.056	0.668	0.084	0.524
TNFRSF12A	-0.079	0.547	0.047	0.723
CCL23	-0.161	0.215	-0.049	0.710
CD5	-0.073	0.574	0.081	0.540
CCL3	0.027	0.839	-0.037	0.780
MMP7	-0.004	0.977	-0.122	0.354
ARG1	-0.021	0.871	-0.054	0.681
NCR1	-0.211	0.102	-0.149	0.255
DCN	0.095	0.465	-0.070	0.594
TNFRSF21	-0.264	0.040*	-0.007	0.960
TNFRSF4	0.010	0.938	-0.041	0.755
MIC-AB	-0.219	0.090	0.100	0.446
CCL17	-0.075	0.566	-0.086	0.512
ANGPT2	-0.076	0.559	0.027	0.841
PTN	0.211	0.103	0.113	0.392
CXCL12	-0.076	0.562	0.017	0.900
IFN-γ	0.089	0.493	0.114	0.384
LAMP3	-0.077	0.554	0.018	0.892
CAS8	0.022	0.864	0.065	0.622
ICOSLG	-0.091	0.484	-0.250	0.054
MMP12	0.090	0.488	0.126	0.336
CXCL13	0.022	0.866	-0.073	0.577
PD_L2	-0.157	0.227	-0.004	0.976
VEGFA	0.064	0.624	0.125	0.342
IL4	-0.070	0.590	0.017	0.899
LAG3	-0.084	0.521	-0.023	0.863
IL12RB1	-0.145	0.267	0.037	0.777
IL13	0.146	0.262	-0.022	0.869
CCL20	0.132	0.309	-0.042	0.748
TNF	-0.032	0.806	-0.027	0.839
KLRD1	-0.104	0.423	0.162	0.215
GZMB	-0.001	0.997	0.012	0.925
CD83	-0.062	0.636	-0.003	0.983
IL12	-0.006	0.961	-0.060	0.646
CSF-1	0.127	0.329	0.135	0.304

Correlations were calculated using the relative levels of *P. micra* and *F. nucleatum.* Abbreviations: r_s, Spearman´s rank correlation coefficient. **P* value <0.05.

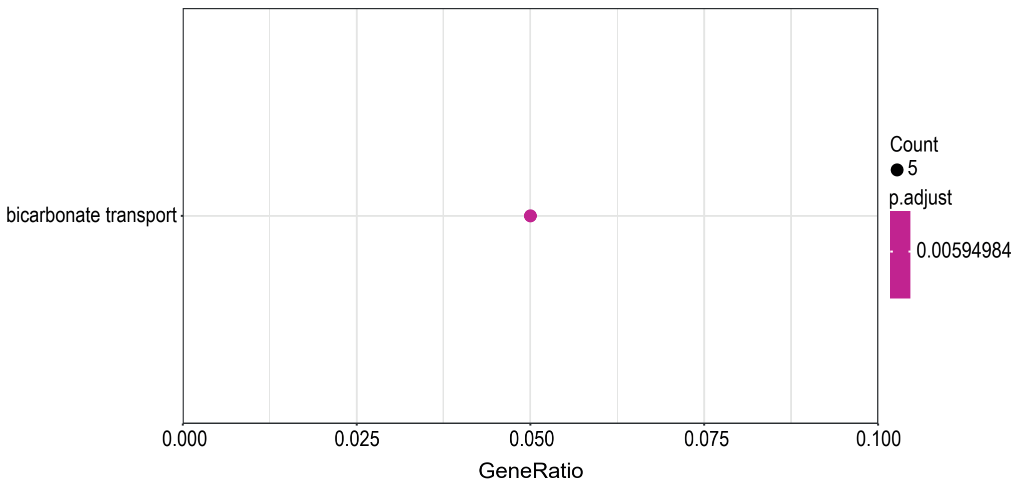

Supplementary Figure 1. GO enrichment analysis for biological processes of differentially expressed genes between *F. nucleatum* positive and *F. nucleatum* negative tumour tissues based on clusterProfiler and the DESeq2 model 2.
